# Bacterial collagenase harnesses collagen geometry for processive cleavage

**DOI:** 10.1038/s41467-026-71099-3

**Published:** 2026-04-02

**Authors:** Hiroya Oki, Katsuki Takebe, Adjoa Bonsu, Kazunori Fujii, Ryo Masuda, Nicholas Henderson, Takehiko Mima, Takaki Koide, Mahmoud Moradi, Osamu Matsushita, Joshua Sakon, Kazuki Kawahara

**Affiliations:** 1 Department of Infection Metagenomics, Genome Information Research Center, Research Institute for Microbial Diseases, The University of Osaka, Osaka, Japan; 2 Department of Dental Pharmacology, Graduate School of Medicine, Dentistry and Pharmaceutical Sciences, Okayama University, Okayama, Japan; 3 Graduate School of Pharmaceutical Sciences, The University of Osaka, Osaka, Japan; 4 Department of Chemistry and Biochemistry, University of Arkansas, Fayetteville, AR USA; 5 Department of Chemistry and Biochemistry, School of Advanced Science and Engineering, Waseda University, Shinjuku-ku, Tokyo, Japan; 6 Waseda Research Institute for Science and Engineering, Waseda University, Shinjuku-ku, Tokyo, Japan; 7 Department of Medical Technology, Faculty of Health Sciences, Ehime Prefectural University of Health Sciences, Ehime, Japan; 8 Department of Bacteriology, Graduate School of Medicine, Dentistry and Pharmaceutical Sciences, Okayama University, Okayama, Japan; 9 Center for Infectious Disease Education and Research, The University of Osaka, Osaka, Japan

**Keywords:** Cryoelectron microscopy, Enzyme mechanisms, Enzymes

## Abstract

Collagen, the major structural protein in the animal extracellular matrix, forms a triple helix that resists proteolysis and requires specialised enzymes for degradation. Flesh-eating bacteria secrete collagenases that unwind the collagen triple helix and processively trim Gly–X–Y triplet repeats, yet the molecular basis of this process has remained obscure. Here, cryo-electron microscopy reveals how *Hathewaya* *histolytica* collagenase ColH engages its substrate and exploits the helix’s architecture for catalysis. ColH encircles a single collagen triple helix in a closed-ring conformation and, through dynamic domain motions, dehydrates and destabilises it. The enzyme undergoes substrate-assisted twisting to adopt a rigid ratcheted conformation, in which one chain is bent into a tripeptide-long ‘bight’ and threaded into the active site for cleavage, while two uncut strands are partitioned to non-catalytic sites. Release of the bight appears to reset the enzyme, with the uncut strands serving as guiding tracks. Repeated cycling between dynamic and rigid states likely enables triplet-by-triplet translocation, allowing ColH to harness collagen’s geometry for processive degradation. These findings reveal a bacterial strategy for collagen unwinding and cleavage distinct from that of mammalian collagenases, highlighting divergent evolutionary solutions for degrading one of nature’s most intractable substrates.

## Abstract (web summary)

Collagen forms a tough triple helix that resists enzymes. Structural analysis suggests a bacterial collagenase grips the helix, loosens one strand for cutting, and uses intact strands as tracks to ratchet forward step by step.

## Introduction

Collagens are the primary structural components of the extracellular matrix (ECM) in animals, where they assemble into fibrillar and laminar networks that confer mechanical strength and maintain tissue organisation^1,2^. Collagens possess a rod-shaped triple-helical domain, which forms a right-handed supercoil of three polypeptide chains—designated leading, middle, and trailing—staggered by one residue^3^. The triple-helical conformation is stabilised by repeating Gly–X–Y motifs, where, most often, X is proline, and Y is 4-hydroxyproline. This densely packed tertiary structure sterically shields peptide bonds, making collagen highly resistant to proteolysis.

To degrade collagen-based structures, bacteria and metazoa produce collagenases, i.e. zinc-dependent metallopeptidases belonging to clan MA in the MEROPS database^4^. Bacterial collagenases belong to the M9 family (gluzincins) based on the sequence similarity^5,6^. M9A collagenases are produced mainly by marine bacteria such as species of *Vibrio* and *Grimontia*, whereas M9B collagenases are produced by terrestrial bacteria such as species of *Clostridium* and *Bacillus*. *Hathewaya histolytica* (formerly *Clostridium histolyticum*) produces two classes of M9B collagenases (ColG and ColH)^7–9^ that act as virulence factors in soft-tissue infections, which have also been adapted for clinical applications, including wound debridement, fibrosis treatment, and islet isolation^10^. Bacterial collagenases contain multiple domains, including an activator domain (AD) and thermolysin-like peptidase domain (PD), which cooperate to unwind and cleave fibrillar collagens^11^. PD-mediated cleavage depends on prior AD-mediated unwinding^12,13^. However, how the enzyme unwinds the triple helix and maintains the unwound state while engaging all three strands remains unclear.

Mammalian matrix metalloproteinases [MMPs, M10 family (metzincins)] have been widely studied and play a tightly regulated role in ECM remodelling. They rely on a hemopexin-like domain to assist unwinding and cleave tropocollagen at a single site via the catalytic domain^14–16^, with activity enhanced under tensile force^17^. Conversely, tensile force inhibits M9 collagenase activity^18^. These opposing responses suggest that M9 and M10 enzymes use fundamentally different unwinding mechanisms^11–13,15,16^, though structural insights into their molecular basis remain limited.

Unlike MMPs, which act exclusively as endopeptidases, bacterial collagenases exhibit both endopeptidase and carboxytripeptidase activities, preferentially cleaving Y–Gly bonds^12,19–22^. The carboxytripeptidase processively trims Gly–X–Y triplets from the initial site of engagement towards the N-terminus. High-speed atomic force microscopy has demonstrated catalysis-coupled movement of a clostridial collagenase in the N-terminal direction^23^, yet how strand engagement supports this processive advancement remains unclear.

Although previous studies reported only the ‘wide-open’ conformation of M9 collagenases^11,12,21,24^, in which the catalytically important AD and PD are spatially separated, this study presents the atomic structures of *H. histolytica* ColH in the ‘closed’ and ‘ratcheted’ states bound to triple-helical peptides. These structures reveal how the AD and PD cooperate to unlay, cleave, and translocate along the triple helix, defining a distinct bacterial mechanism for processive collagenolysis.

## Results

### ColH adopts ‘closed’ conformation

**Fig. 1:**
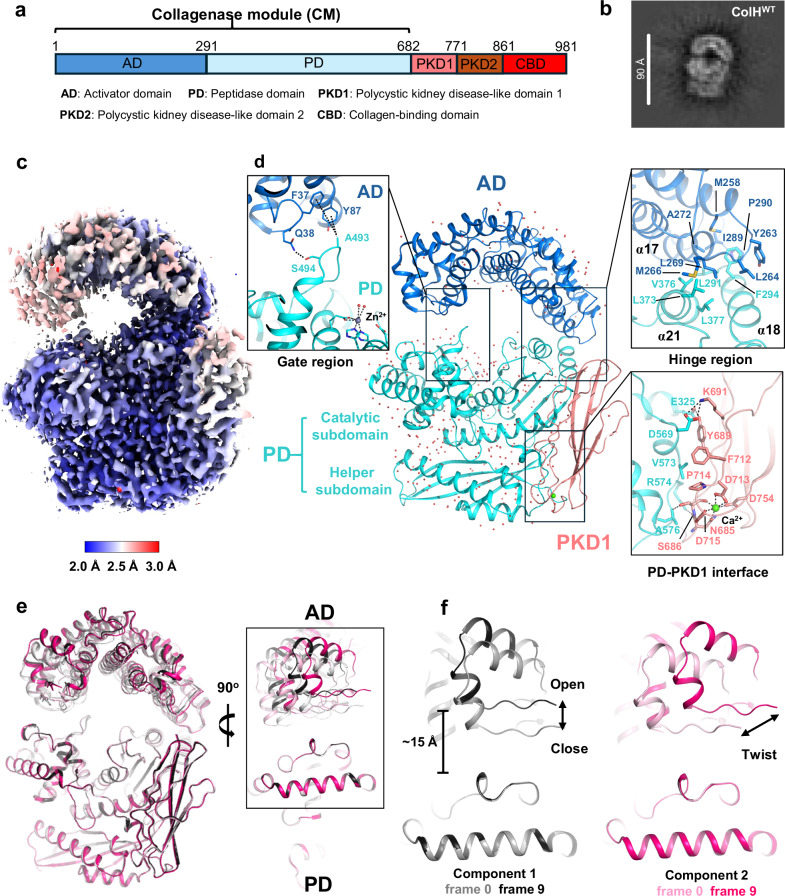
Conformational dynamics of ColH. **a** Domain organisation of ColH. **b** Representative 2D class average of ColH^WT^. **c** Consensus cryo-EM density map of ColH^WT^, coloured according to local resolution. **d** Atomic model of ColH^WT^ composed of the AD, PD, and PKD1, coloured by domain. PKD2 and CBD were not modelled due to the absence of observable EM density. Water molecules are depicted as red spheres. Calcium and zinc ions are shown as green and grey spheres, respectively. Magnified views of the gate region (upper left panel), hinge region (upper right panel), and the interface between PD and PKD1 (lower right panel) are shown. Interacting residues are represented as stick models. **e** Superimposition of 3DVA structures for first and second variability components. The initial (frame 0) and final (frame 9) conformational states are shown for the first (grey and black) and second (light pink and pink) principal components, respectively. **f** Superimposition of initial (frame 0) and final (frame 9) states illustrating first (left panel) and second (right panel) principal components—representing ‘close–open’ and ‘twist’ motions of ColH^WT^, respectively.

To elucidate the structural mechanisms underlying bacterial collagenase function, we performed cryo-electron microscopy (cryo-EM) on *H. histolytica* wild-type ColH (ColH^WT^; Fig. 1a–d and Supplementary Figs. 1 and 2, and Supplementary Table 1). The 2D class averages showed that ColH^WT^ adopts a ring-like conformation of its collagenase module (CM), comprising AD and PD (Fig. 1b). The consensus cryo-EM density map, resolved at an average resolution of 2.3 Å, was well-defined for the PD but showed conformational heterogeneity in the AD (Fig. 1c). Focused refinement improved AD map quality, revealing a rigid structure with orientational flexibility relative to the other domains (Supplementary Fig. 1). Our cryo-EM analysis revealed a clear density for polycystic kidney disease-like domain 1 (PKD1) but not PKD2 or the collagen-binding domain (CBD), likely owing to linker flexibility that averages out their densities in the EM reconstruction.

The composite EM map of the AD and PD-PKD1 regions revealed a ‘closed’ CM conformation (Fig. 1d and Supplementary Figs. 1 and 2). At the junction between AD and PD, three helices (α17, α18, and α21) formed a hinge via hydrophobic interactions, whereas gate-region interactions on the opposite side reinforced the ring (Fig. 1d). PKD1 interacts with the PD in a calcium-dependent manner (Fig. 1d). A previous small-angle X-ray scattering experiment showed that calcium depletion produces an elongated apo ColH envelope^25^. Thus, the rigid PKD1 conformation may stabilise the overall structure and restrict PKD2-CBD orientation. We also performed X-ray crystallography of ColH^WT^ CM at room temperature, which yielded a 2.70 Å structure essentially identical to the cryo-EM model, with a root-mean-square deviation of 1.9 Å for Cα atoms (Supplementary Fig. 3 and Supplementary Table 2). Notably, crystallographic B-factors increase progressively from the C-terminus to the N-terminus of the AD, suggesting hinge-mediated flexibility between the AD and PD (Supplementary Fig. 4).

These structural analyses suggest that ColH^WT^ CM can accommodate a single triple-helical molecule ( ~ 15 Å diameter) in its central cavity, but gate interactions require domain opening for substrate access. To assess CM flexibility, the cryo-EM data were used for 3D variability analysis (3DVA)^26^, classifying particles along principal component axes. ColH^WT^ adopts a dynamic ‘closed’ state in which CM domains undergo partial opening and twisting (Fig. 1e, f, Supplementary Fig. 5 and Supplementary Movies 1 and 2). Although low in amplitude, these motions generate an entrance of ~15 Å, sufficient to capture a tropocollagen molecule in the central cavity (Supplementary Fig. 6).

At the catalytic centre, a Zn²⁺ ion was coordinated by the conserved residues, His415, His419, and the bidentate Glu447, as well as two water molecules (w1 and w2), as observed consistently in both the cryo-EM and X-ray crystal structures, adopting hexacoordinate geometry (Supplementary Fig. 7). Neither water molecule was positioned to be deprotonated by Glu416.

### ColH targets triple helix at C-terminus

**Fig. 2:**
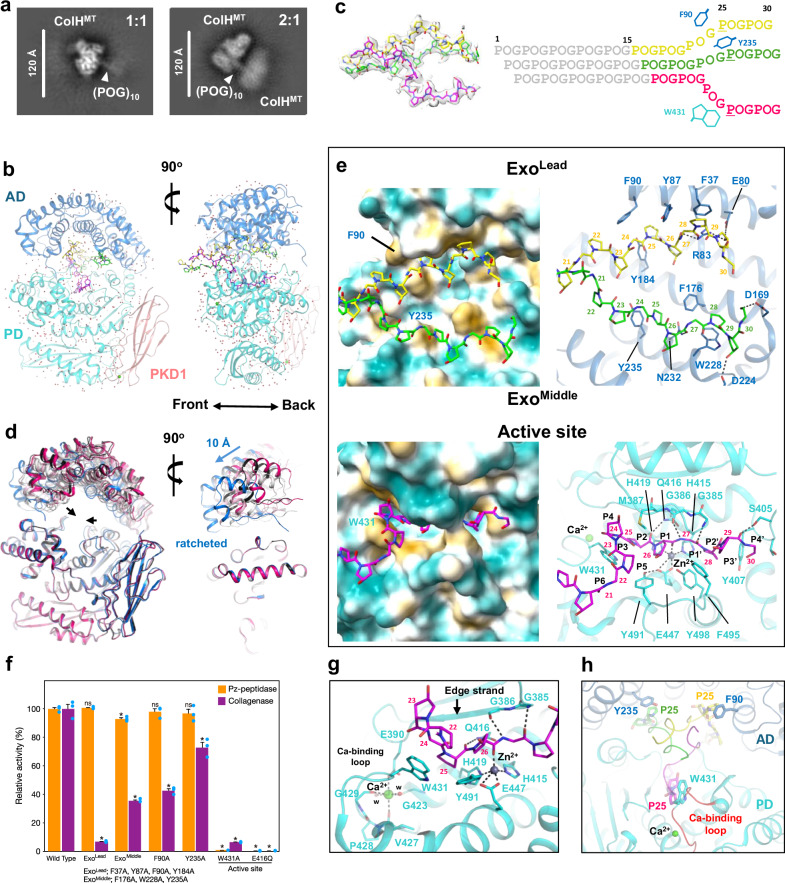
ColH binds to unwound C-terminus of (POG)_10_. **a** Representative 2D class averages of ColH^MT^–(POG)_10_ complex. **b** Atomic model of 1:1 ColH^MT^–(POG)_10_ complex. The leading, middle, and trailing strands of (POG)_10_ are coloured yellow, green, and magenta, respectively. Front and back faces of ColH^MT^ are indicated by a double-headed arrow. **c** EM map (left) and sequence of (POG)_10_ (right). Three aromatic residues (F90, Y235, and W431) interacting with Pro25 (underlined) of each strand are shown. **d** Comparison of ‘ratcheted’ ColH^MT^ in the complex (blue) and dynamic ‘closed’ ColH^WT^ conformations. A magnified view of the lumen showing contraction of the active-site loops (arrows). **e** Interfaces between the leading/middle strands and AD (top) and between the trailing strand and PD (bottom). Surface hydrophobicity of AD (top left), and PD (bottom left), calculated using Chimera^43^ with the Kyte–Doolittle scale: blue, hydrophilic; white, neutral; orange, hydrophobic. **f** Relative Pz-peptidase (orange) and collagenase (purple) activities of wild-type and mutant ColH. All assays were performed in triplicate across three independent experiments. Data are presented as mean values ± SEM. Blue dots represent the mean values of each triplicate. The statistical differences between wild-type and mutant ColH were examined by a two-tailed *t*-test. The exact *p* values for Pz-peptidase and collagenase activities were 0.574 and 1.28 × 10^−3^ for Exo^Lead^, 9.26 × 10^−3^ and 2.05 × 10^−3^ for Exo^Middle^, 0.488 and 1.07 × 10^−5^ for F90A, 0.408 and 9.34 × 10^−3^ for Y235A, 4.83 × 10^−5^ and 1.15 × 10^−6^ for W431A, and 1.09 × 10^−4^ and 1.19 × 10^−3^ for E416Q, respectively; *, *p* < 0.01; ns, non-significant (*p* ≥ 0.01). Source data are provided. **g** Magnified view of active-site cleft around Trp431. The Trp431 residue, located on a Ca-binding loop, is shown as a stick model. **h** Magnified views showing the three aromatic residues (F90, Y235, and W431) stabilising unwound strands, viewed from the front face of the ColH^MT^–(POG)_10_ complex. The calcium-binding loop is shown in red.

To determine the substrate recognition and unwinding mechanisms, cryo-EM was performed on ColH complexed with the collagenous triple-helical peptide [(Pro-Hyp-Gly)₁₀]₃ [or (POG)_10_] (Fig. 2, Supplementary Figs. 8, 9, 10 and Supplementary Table 1). Here, Hyp (or O) represents 4-hydroxyproline. (POG)_10_ adopts a stable triple helix in solution (*T*_M_ = 60 °C)^27^. Since ColH hydrolyses (POG)_10_ (Supplementary Fig. 11), we used a protease-null ColH mutant containing the Glu416Gln (E416Q) substitution (ColH^MT^).

Image analysis showed that ~80% of particles formed a 1:1 complex, with a rod-like (POG)_10_ density protruding from one ColH^MT^ face (Fig. 2a and Supplementary Fig. 8), whereas the remainder formed a 2:1 complex. The 1:1 complex was resolved at 2.7 Å without conformational heterogeneity except in the PKD2–CBD region. ColH^MT^ selectively interacted with the C-terminal region of (POG)_10_, unwinding its terminal three triplets (residues 22–30). Recognition was unidirectional, with N-to-C binding of the unwound region from the front to the back face of ColH^MT^ (Fig. 2b, c). Neither reverse-direction recognition nor recognition involving unwinding of the central region or N-terminal side of (POG)_10_ was detected in the cryo-EM analysis. Assuming that ColH^MT^ can topologically entrap any site of (POG)_10_ within its CM ring, this was not initially predicted. A previous study indicated that unwinding by M9B collagenase tends to be a local process^13^; therefore, ColH^MT^ likely recognises a readily or sufficiently unwound region during N-to-C translocation, adopts a stable conformation through unidirectional binding at that site, and subsequently becomes arrested.

The N-terminal region (residues 1–15) of (POG)_10_ remained triple-helical but was visible only at low contour levels (Supplementary Fig. 8). A triple-helix model was therefore built only for residues 16–21, where density was well defined (Fig. 2c). While ColH^MT^ retains a ring-like conformation, resulting in only localised unwinding, structural comparison with ColH^WT^ showed that the AD undergoes a pronounced twist of ~10 Å relative to the PD and is conformationally locked (Fig. 2d). Substrate binding thus shifts ColH toward a distorted rigid ‘ratcheted’ state, distinct from the dynamic ‘closed’ state.

Partial unfolding at the C-terminus allows the leading and middle strands of (POG)_10_ to bind to two AD exosites (Exo^Lead^ and Exo^Middle^; Fig. 2b, e). Both sites engage two contiguous triplets (Pro25–…–Gly30) via hydrophobic clefts. Proline residues at X positions (Pro25 and Pro28) form contacts with aromatic residues—Phe37, Tyr87, Phe90, and Tyr184 at Exo^Lead^; Phe176, Trp228, and Tyr235 at Exo^Middle^—through hydrophobic and CH–π interactions (Fig. 2e). Alanine substitution of these residues significantly reduced type I collagen hydrolysis but not 4-phenylazobenzyloxycarbonyl-Pro-Leu-Gly-Pro-_D_-Arg (Pz-peptide) hydrolysis, highlighting their role in triple-helix recognition and unwinding (Fig. 2f). Mutations in the Exo^Lead^ residues caused a substantially greater reduction in collagenolytic activity than those in Exo^Middle^ ( ~ 90% versus ~60%), suggesting that Exo^Lead^ plays a more critical role in collagen recognition and unwinding. Sequence and structural comparisons with M9A and M9B collagenases showed that Exo^Lead^ residues are strictly or highly conserved across these enzymes (Supplementary Figs. 12 and 13). Consistently, their importance for collagenolytic activity in both subfamilies has been confirmed in previous mutational studies^12,13^. In contrast, the aromatic residues comprising Exo^Middle^ in ColH are intriguingly not as well conserved within the M9 family. In the M9B subfamily, although Y235 and W228 are conserved, F176 is not, whereas in M9A, only the residue equivalent to W228 is conserved. This relatively low level of conservation may underlie the variability in substrate binding at this exosite.

Besides the aromatic interactions, several hydrogen-bonding interactions were observed (Fig. 2e). At Exo^Lead^, such interactions stabilise the leading-strand conformation: the main-chain carbonyls of Hyp26 and Gly27 hydrogen bond with Arg83, whereas the OD1 atom of Hyp29 forms hydrogen bonds with Glu80 and Arg83. Although hydrogen-bonding networks at Exo^Middle^ are less extensive, an interaction between the carbonyl group of Hyp26 and Asn232 as well as OD1 of Hyp29 and carbonyl group of Asp224 stabilises the middle strand. While these interactions further strengthen peptide binding, the residues are not strictly conserved within the M9 family, with the exception of Arg83 (Supplementary Figs. 12 and 13). The importance of arginine at this position for substrate recognition in M9A and M9B collagenases has been demonstrated in mutational studies^12,13^, consistent with our structural observation that Arg83 engages and stabilises the main chain of the leading strand.

Most Hyp residues remained solvent-exposed and contributed little to binding, consistent with the cryo-EM data of ColH^MT^ bound to [(Pro–Pro–Gly)₁₀]₃ [or (PPG)_10_] (Supplementary Fig. 14 and Supplementary Table 1), which revealed a binding mode similar to that of the ColH^MT^–(POG)_10_ complex (Supplementary Fig. 15). These results suggest that ColH has evolved to recognise Gly–Pro–Yaa–Gly–Pro–Yaa motifs in native collagen via its hydrophobic exosites, consistent with previous proteomics analyses demonstrating the substrate specificity of clostridial collagenases for Gly–Pro–Yaa triplets^28^.

Although the leading and middle strands followed a triple-helical axis with minor deviation, the trailing strand veered sharply, threading the three triplets at its C-terminus (Gly21–…–Gly30; P6 to P4′) into the active-site cleft (Fig. 2c, e). This distortion is critical for Zn²⁺ coordination of the scissile carbonyl oxygen. As observed in the inhibitor-bound structures of other M9B collagenases^11,24^, the active-site cleft embraced by the N- and C-terminal half-domains of the PD catalytic subdomain was contracted as much as 3 Å, holding the trailing strand tightly (Fig. 2d and Supplementary Fig. 16). In the cleft, the Hyp26 (P1)–Gly27 (P1′) scissile motif was stabilised by antiparallel interactions with conserved Gly385 and Gly386 on the β4 edge strand, known as the double-glycine S1′ recognition site^11,24^. Ser405 of the ‘wall motif’^24^ engaged the Hyp29 (P3′) carbonyl, acting as a molecular ruler for substrate recognition. On the opposite side, Trp431 interacted with Pro25 (P2) and the Cα–H of Gly24 (P3) through CH–π interactions and, together with Tyr491, interacted with Pro22 (P5) via nonpolar contacts and shape complementarity, thereby stabilising the three-residue bight (Fig. 2g). Notably, Trp431 lies on a calcium-binding loop located between the N- and C-terminal half-domains, where a conserved glutamate side-chain (Glu390 in ColH) anchors the edge strand and calcium, thereby bracing the two half-domains (Fig. 2g). Consequently, calcium binding not only stabilises zinc coordination in the active site but also ensures the proper positioning of Trp431. These observations support the essential role of Ca²⁺ coordination in ColH catalysis^24^.

When viewed from the front face of ColH^MT^, three aromatic residues, Phe90, Tyr235, and Trp431, engaged the interacting triplets as aromatic pivots marking the transition from a helical to unwound structure (Fig. 2h). These residues may promote local bending to promote strand unwinding or favour partially unfolded triple-helical structures, facilitating recognition of the frayed C-terminal region of (POG)_10_^29^. Substitution of Phe90 greatly reduced collagenolytic activity, whereas Tyr235 mutation led to a more modest decrease ( ~ 60% versus ~30%; Fig. 2f). Although Tyr235 is not conserved in the M9A family (Supplementary Fig. 12), a phenylalanine residue that may serve a similar function is positioned nearby (Supplementary Fig. 13). This suggests that an aromatic residue at this position supports the binding and stabilisation of the unwound collagen triple helix.

Strikingly, the substitution of Trp431 with alanine resulted in a dramatic decrease ( ~ 95%) in collagenolytic activity and almost complete loss of enzymatic activity toward Pz-peptide, comparable to that observed for the catalytically inactive E416Q mutant (Fig. 2f). Circular dichroism (CD) measurements confirmed comparable structures between the mutants and ColH^WT^ (Supplementary Fig. 17). Trp431 lies near the catalytic centre (within 10 Å from zinc to the indole ring) via the calcium-binding loop, and its indole ring stabilises Pro25 (P2) via a CH–π interaction (Fig. 2g). A structural model of ColH with Pz-peptide based on the ColH^MT^–(POG)_10_ complex indicated that this interaction is also responsible for the loss of Pz-peptidase activity, as the Pro1 residue of Pz-peptide is predicted to be in the S2 subsite and interacts with Trp431 (Supplementary Fig. 18). These results further substantiate the critical role of Trp431 in ensuring stable substrate binding and correct orientation within the active-site cleft for efficient hydrolysis.

At the catalytic centre, the scissile peptide carbonyl oxygen coordinated the Zn²⁺ ion, increasing polarisation of the carbonyl bond and priming it for nucleophilic attack. No ordered water molecules were observed near either the scissile bond or residue Gln416. The carbonyl oxygen lies too close (3.2 Å) to the amide oxygen of Gln416, resulting in a non-productive pentacoordinate geometry and complete loss of hydrolytic activity (Fig. 2f). Since the corresponding distance in a wild-type structure of *Grimontia* collagenase Ghcol in complex with (GPO)_2_ is 4.6 Å^30^, enough to evoke a substrate water molecule to the space, Glu416 in ColH^WT^ may deprotonate a water molecule to initiate a nucleophilic attack.

### ColH recognises triple-helical substrate

**Fig. 3:**
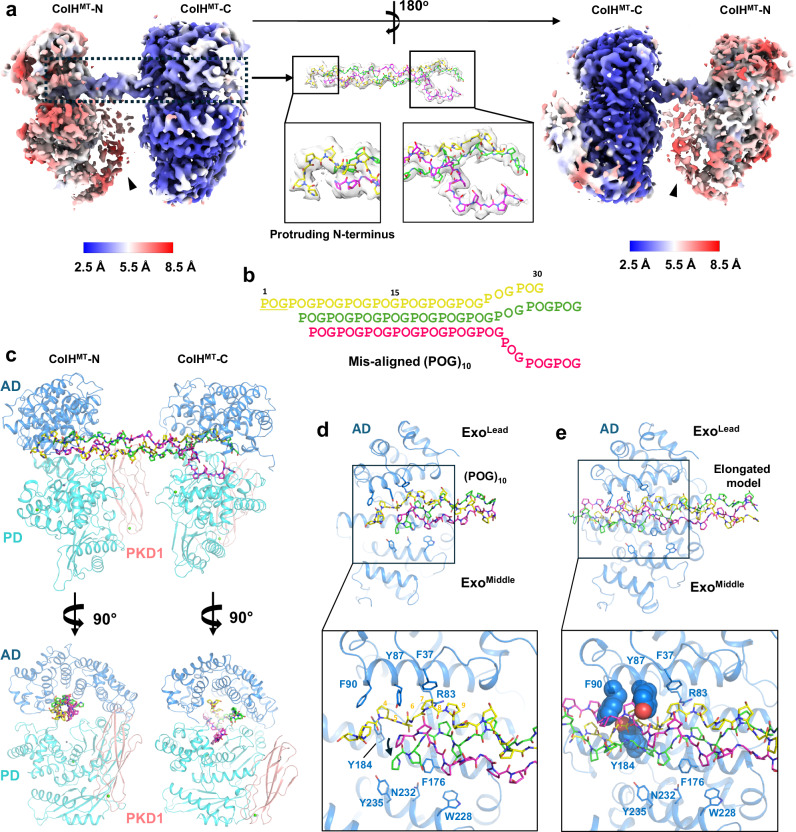
ColH binds to N-terminus of (POG)_10_ but does not unwind triple helix. **a** Consensus cryo-EM density map of 2:1 ColH^MT^–(POG)_10_ complex, coloured according to local resolution with the EM density map of (POG)_10_, showing a mis-staggered triple helix. Arrowheads show the EM density of PKD1, which is at lower resolution due to steric clashes with ColH^MT^-C. **b** Mis-staggered (POG)_10_ sequence. The N-terminal hanging triplet of the leading strand is underlined. **c** Atomic model of 2:1 ColH^MT^–(POG)_10_ complex derived from the composite cryo-EM density map. ColH^MT^-N and -C indicate ColH^MT^ molecules bound at the N- and C-termini, respectively. A back-side view of each ColH^MT^ molecule is shown (bottom). **d** Interface between N-terminal region of (POG)_10_ and AD of ColH^MT^-N (top). A magnified view of the interacting region is shown (bottom). The arrow indicates a deviation of the N-terminal proline residue of the middle strand (green) from the triple-helical axis. **e** Model of a complex in which the substrate triple helix is extended by two triplets toward the N-terminus. The magnified view of the interacting region shows clashes with aromatic residues F90, Y87 and Y184.

During image processing, we observed particles of a 2:1 complex (Fig. 2a). 3D reconstruction revealed one ColH^MT^ molecule bound to the (POG)_10_ C-terminus (ColH^MT^-C) and another to the N-terminus (Fig. 3a and Supplementary Fig. 9). Consensus maps and 3DVA revealed ColH^MT^-N to be more dynamic than ColH^MT^-C, resulting in a lower resolution for ColH^MT^-N (Supplementary Fig. 10 and Supplementary Movie 3). In contrast, (POG)_10_ density was well resolved and unexpectedly revealed a one-triplet mis-stagger in the leading strand (Fig. 3a, b). Although such mis-staggered helices are rare^31^, they may have facilitated additional ColH^MT^ recruitment under our experimental conditions.

Local refinement improved the resolution to 3.4 Å for ColH^MT^-N and 3.1 Å for ColH^MT^-C, enabling model building (Fig. 3c and Supplementary Fig. 9). In the 2:1 complex, ColH^MT^-N interacted with the leading strand via Exo^Lead^ of the AD without major unwinding, while ColH^MT^-C unwound the mis-staggered C-terminus similarly to the 1:1 complex (Fig. 3c, d). These results highlight the critical role of Exo^Lead^ in triple-helix recognition. As with ColH^MT^-C, the hydrophobic residues Phe37, Tyr87, and Phe90 mediated Exo^Lead^ binding. Although the protruding N-terminal triplet may aid initial recognition, ColH^MT^ interacted more specifically with the Pro4–Hyp5–Gly6–Pro7–Hyp8–Gly9 segment (Fig. 3d). The N-terminal triplet adopted a bent conformation, likely due to steric clashes with aromatic residues (Tyr87, Phe90, and Tyr184). We also observed a slight displacement of the middle chain (Pro1–Hyp2) toward Exo^Middle^. These results suggest that Exo^Lead^ aromatic residues promote local destabilisation of the triple helix during ColH translocation from the N- to the C-terminus of tropocollagen. This interpretation is supported by the extended (POG)n model, which shows steric clashes at Exo^Lead^ (Fig. 3e). Nevertheless, Exo^Lead^-driven unwinding of (POG)_10_ in the complex may be restricted by steric hindrance between the PKD1 of ColH^MT^-N and CM of ColH^MT^-C already bound at the C-terminus (Fig. 3a and Supplementary Fig. 19). This steric hindrance could impede C-terminal progression of ColH^MT^-N and thereby limit further N-terminal unravelling of (POG)_10_, potentially reducing stable engagement of the partially unwound middle and trailing strands by Exo^Middle^ and the active site, respectively.

### ColH unwinds triple-helical substrate

**Fig. 4:**
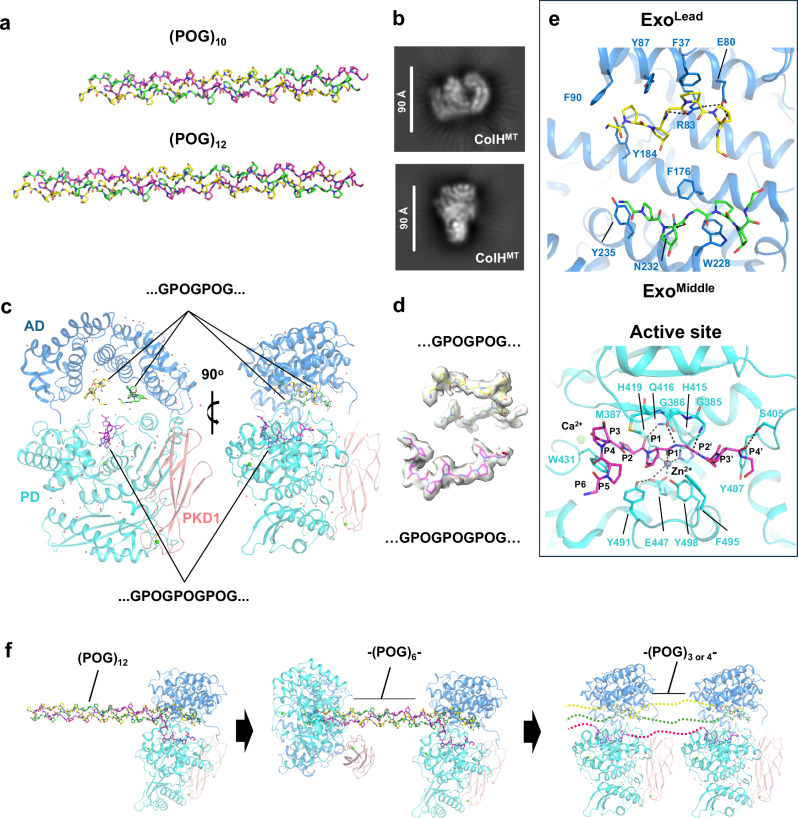
ColH unwinds triple helix of (POG)_12_. **a** Structural comparison of (POG)_10_ and (POG)_12_, generated based on (POG)_10_ structure (PDB ID: 3B0S). **b** Representative 2D class averages of ColH^MT^–(POG)_12_ complex. **c** Atomic model of ColH^MT^–(POG)_12_ complex. Resolved regions of (POG)_12_ were observed at the same exosites and active site as in the ColH^MT^–(POG)_10_ complex. Sequences corresponding to (POG)_12_ regions are indicated (top and bottom). **d** EM density map of (POG)_12_ regions. **e** Interfaces between two resolved (POG)_12_ strand segments and AD (top) and between trailing strand segment and PD (bottom). **f** ColH binding and (POG)_12_ unwinding. After a ColH^MT^ molecule binds to the unwound C-terminus of (POG)_12_ (left), an additional ColH^MT^ molecule binds to the (POG)_12_ N-terminus in a manner similar to ColH^MT^-N in the ColH^MT^–(POG)_10_ complex. This leaves approximately six triplets in the central region of (POG)_12_ in a triple-helical state (middle). The N-terminal ColH^MT^ destabilises the helix and translocates to the C-terminus, locally unwinding the structure. The intervening triple helix, with only 3–4 triplets, is insufficient to maintain a stable helical conformation.

To improve the resolution of the 2:1 complex, we employed a longer peptide, [(Pro–Hyp–Gly)₁₂]₃ [or (POG)_12_] (Fig. 4a, Supplementary Fig. 20, and Supplementary Table 1). Unlike for (POG)_10_, 2D class averages of the ColH^MT^–(POG)_12_ complex showed only a 1:1 assembly (Fig. 4b), yielding an unanticipated but informative structure. The 3D reconstruction (2.8 Å resolution) resembled ColH^WT^ in the ‘closed’ state, with the AD displaying conformational heterogeneity rather than the rigid ‘ratcheted’ conformation (Supplementary Fig. 20). Local refinement improved AD and PD–PKD1 resolutions to 2.9 and 2.8 Å, respectively. Residual EM density for (POG)_12_ was observed at three positions: Gly–Pro–Hyp–Gly–Pro–Hyp–Gly motifs at Exo^Lead^ and Exo^Middle^ and a longer Gly–Pro–Hyp–Gly–Pro–Hyp–Gly–Pro–Hyp–Gly stretch at the active site (Fig. 4c–e). These binding modes mirrored those in the 1:1 ColH^MT^-C–(POG)_10_ complex (Figs. 2e and 4e). Even at low contour levels, no triple-helical density was detected, indicating complete unwinding of (POG)_12_ by ColH^MT^.

We initially hypothesised that one ColH^MT^ molecule would unwind the three C-terminal triplets of (POG)_12_, allowing a second ColH^MT^ to bind to the N-terminus—either capping it or unwinding the first three triplets—leaving the central six triplets as a stable helix, since (Pro–Hyp–Gly)₆ forms a helix at room temperature (Fig. 4f)^32^. However, this was inconsistent with the 2D class averages (Fig. 4b and Supplementary Fig. 20). Instead, cryo-EM revealed complete unfolding of (POG)_12_, suggesting that the additional ColH^MT^ binding locally promotes progressive destabilisation or unwinding in an N-to-C direction, likely in a spiral manner, approaching the bound ColH^MT^, until a steric hindrance occurs due to its PKD1. The remaining central segment, now reduced to 3–4 triplets, appears insufficient to maintain a triple-helical structure (Fig. 4f). Here, we cannot exclude that a second ColH^MT^ binds adjacent to ColH^MT^-C and subsequently migrates toward the N-terminus, inducing global unwinding of (POG)_12_. Once fully unwound, the two molecules are difficult to analyse collectively owing to their orientational flexibility or possible dissociation of one molecule from the unwound peptides.

Since the CM in the ColH^MT^–(POG)_12_ complex shows a dynamic ‘closed’ state, these findings indicate that ColH can adopt the ‘ratcheted’ state only when the collagen substrate remains partially unwound, with the N-terminal side retaining its triple-helical structure. These results suggest that ColH exploits the intrinsic architecture of the triple helix to achieve the ratcheted state—a transient conformation that mediates strand unlaying, strand-specific cleavage, and N-terminal-directed translocation, as discussed in the following section.

## Discussion

**Fig. 5:**
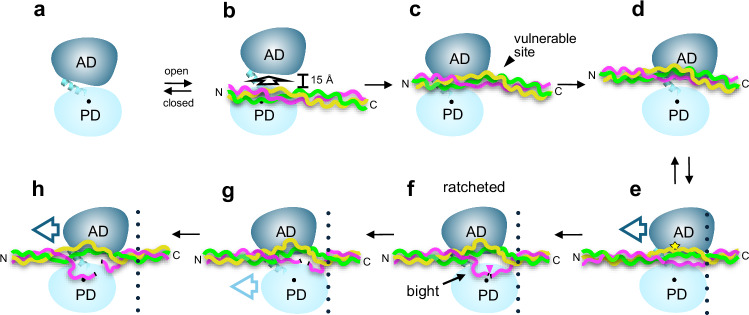
Proposed mechanism of collagen recognition, unwinding, and processing by bacterial collagenase. **a**, **b** Thermal fluctuations between AD and PD of ColH enable recruitment of tropocollagen molecule. **c** Following one-dimensional translocation along the collagen molecule, ColH engages vulnerable sites. **d**, **e** The hydrophobic surface within the lumen of the ring-like structure dehydrates and destabilises the triple-helical structure. Here, conserved aromatic residues may destabilise the triple helix through steric hindrance (indicated by a yellow star). **f** Upon stably binding to an unfolded triple-helical region in the rigid ‘ratcheted’ state, with a bight in the trailing strand, ColH cleaves the peptide bond. **g** Following cleavage, the two resulting fragments dissociate from the active site. The N-terminal fragment, previously in a bight conformation, can readily rebind at the S3–S3′ positions. During this process, the ‘ratcheted’ conformation relaxes and the PD shifts toward the N-terminal side of collagen, while the leading and middle strands remain in contact with the AD. **h** Once the trailing strand is firmly recognised by the active site, the leading and middle strands transiently dissociate from the AD and subsequently rebind, re-establishing the ‘ratcheted’ conformation seen in **f**.

The structural, biochemical, and biophysical insights gained from this and previous studies^11–13,17–19,21,23–25^ explain how bacterial collagenases recognise the collagen triple helix and exploit its geometry for processive strand-specific cleavage. Current evidence supports a model in which *H. histolytica* ColH alternates between a dynamic ‘closed’ and a rigid ‘ratcheted’ conformation, linking local helix unwinding to triplet-by-triplet catalysis (Fig. 5).

In contrast to mammalian collagenases such as MMP1, which bind collagen side-on to cleave it at a distinct site^15^, ColH encloses a single tropocollagen molecule within a ring-like CM, thereby topologically securing the substrate for processive cleavage (Fig. 5a, b). As shown in the ColH^MT^–N–(POG)_10_ complex, which represents an unproductive binding mode likely arising from a mis-staggered triple helix and/or steric hindrance by ColH^MT^-C, the CM appears to use the captured substrate as ‘rail tracks’ for one-dimensional diffusion. This directional movement may enable the enzyme to search for vulnerable regions, reminiscent of the behaviour of DNA-binding clamps and endonucleases (Figs. 3 and 5c, d)^33,34^. The lumen of the CM is predominantly hydrophobic, unlike the charged lumens of DNA-binding proteins.

Recognition relies on two AD exosites, primarily Exo^Lead^, which orient consecutive Gly–Pro–Yaa triplets via hydrophobic and CH–π interactions. Although less extensive, several hydrogen-bonding interactions, particularly those involving the conserved Arg83 at Exo^Lead,^ contribute to peptide strand positioning. This mode of binding likely disrupts water-mediated hydrogen bonds that stabilise the collagen triple helix, consistent with the ‘chew-and-digest’ mechanism proposed for ColG^11^. Conserved aromatic residues—Phe90, Tyr235, and Trp431—act as pivot points at the helical–unwound junction, bending and destabilising the helix (Fig. 2h). These residues may also help ColH recognise vulnerable sites in tropocollagen as it slides along the triple helix (Fig. 5c, d). Notably, the dynamic ‘closed’ conformation of ColH is in stark contrast to that of homologous enzymes such as ColG and some members of the M9A subfamily, which adopt more widely opened AD–PD arrangements (Supplementary Fig. 21)^11,12,21^. The closed-biased architecture of ColH may enhance its ability to capture rare, thermally populated local fluctuations of the collagen triple helix, thereby promoting active unwinding.

Once unwinding of the triple helix is achieved (Fig. 5e, f), ColH establishes asymmetric contacts: the leading and middle strands engage AD exosites, while the trailing strand inserts into the PD active site. Recognition of two triplets at each exosite and three triplets at the active site induces a conformational twist between AD and PD (Fig. 5f). In this ‘ratcheted’ state, the trailing strand adopts a three-residue bight (Pro22–Gly24), which is stabilised by Trp431 within the S3–S5 pocket. The aromatic residue at the S3–S5 pocket is highly conserved among bacterial M9 collagenases, suggesting a shared mechanism for substrate recognition (Supplementary Fig. 22). This captured bight is critical for translocation and reloading of the substrate, enabling next-step cleavage of a Gly–X–Y tripeptide. Mechanical strain on collagen fibrils, known to inhibit M9 collagenases^18^, likely does so by preventing bight formation, whereas MMP1 is activated by tensile force^17^, underscoring the mechanistic divergence between bacterial and mammalian enzymes.

The cleavage mode also differs between bacterial collagenases and MMPs. MMPs act exclusively as endopeptidases, whereas ColH advances toward the N-terminus by releasing trailing-strand tripeptides through carboxytripeptidase activity. Here, we present a refined model in which, after each cleavage event, the three-residue bight passively dissociates from the active-site cleft due to reduced post-cleavage affinity. The next triplet then moves into the S1′–S3′ subsites as the CM domains realign (Fig. 5g, h). This requires transient loosening of AD–PD interactions, allowing the AD to twist back by ~10 Å (one triplet) (Fig. 2d). A conserved ‘wall motif’ in the distal S3′ pocket, distinct to M9B collagenases, enforces this stepwise C-to-N progression. Intermittent AD engagement with the leading and middle strands further ensures triplet-spaced translocation. Through cycles of ‘closed’ and ‘ratcheted’ states, ColH trims one trailing-strand triplet at a time, consistent with the C-to-N directionality previously observed for ColG at the fibril level^23^. Nonetheless, this model is mainly inferred from structural data, lacking direct kinetic or biochemical evidence; it must therefore be interpreted with caution and validated in future experimental studies.

Additionally, the present study leaves the unresolved question of how ColH overcomes steric constraints within densely packed collagen fibrils. The PKD2–CBD region of the collagenase may act as an auxiliary tether that recognises locally distorted or partially unbundled collagen regions^35,36^. By transiently anchoring ColH to these hypersensitive sites, the PKD2–CBD region may help position the triple helix into the catalytic cleft, thereby facilitating localised unwinding and cleavage. This auxiliary targeting strategy may enhance catalytic efficiency by enabling surface diffusion and selective engagement of vulnerable sites, rather than relying solely on random encounters with an insoluble substrate. Nevertheless, the precise mechanism by which ColH achieves efficient fibril hydrolysis remains unclear, which will require further investigation.

In conclusion, ColH leverages the geometric constraints of the collagen triple helix to gain catalytic advantage. By coupling local unwinding to strand-selective binding and directional trimming, ColH achieves processive, strand-specific cleavage resembling a ‘burnt-bridge Brownian ratchet’^37^. This mechanism, likely conserved among M9 enzymes, explains how collagen stability is overcome and suggests strategies for engineering collagenases with enhanced activity or specificity. It also highlights structural features that may be targetable by inhibitors designed to disrupt domain motions or critical interfaces.

## Methods

### Plasmids

*Escherichia coli* DH5α was used for plasmid construction. To express ColH^WT^, the plasmid pGEX-ColH was generated by amplifying the coding sequence of mature ColH (Val1–Arg981) using the primer pair 5′-ggatccGTACAAAATGAAAGTAAGAGGTATAC-3′ and 5′-ctcgagTTATCTTCCTACTGAACCTTCTATAT-3′ (lowercase letters indicate *Bam*HI and *Xho*I recognition sites, respectively), followed by insertion into pGEX-4T-2 (Cytiva) at the *Bam*HI and *Xho*I sites. Mutant ColH plasmids were generated by site-directed mutagenesis using a PrimeSTAR Mutagenesis Basal Kit (TaKaRa Bio) with the primers listed in Supplementary Table 3. To express the ColH^WT^ collagenase module (CM; Val1–Ser686), pGEX-ColHCM was constructed by PCR amplification with primers 5′-cgcggatccGTACAAAATGAAAGTAAGAGGTATACA-3′ and 5′-ccgctcgagTTATGAATTTTTGGAATCACCTTCGTTTGG-3′ (lowercase letters indicate flanking trinucleotides followed by *Bam*HI and *Xho*I sites, respectively) and inserted into pGEX-4T-2. All constructs were verified by using Sanger sequencing.

### Purification of wild-type and mutant ColH

*E. coli* BL21-CodonPlus-RIL (Agilent Technologies) cells transformed with plasmids encoding ColH^WT^ or mutants were grown in 2YT medium (1.6% tryptone, 1.0% yeast extract, 0.5% NaCl) containing 2% glucose, 50 µg/mL ampicillin, and 30 µg/mL chloramphenicol at 37 °C until reaching an OD_600_ of 1.0. Cultures were cooled in an ice bath for 10 min, and expression was induced with 1 mM isopropyl-β-D-thiogalactopyranoside at 25 °C for 20 h. Cells were harvested by centrifugation at 7500 *g* and 4 °C for 10 min and resuspended in phosphate-buffered saline (PBS) with 1 mM phenylmethylsulfonyl fluoride. Cells were disrupted using a French press at 10 000 psi, treated with Triton X-100 (1% final concentration), and the lysate was rotated at 4 °C for 30 min before clarification by centrifugation at 25 000 *g* and 4 °C for 30 min, repeated twice. The cleared lysate was incubated with Glutathione Sepharose 4 Fast Flow beads (Cytiva) at 4 °C for 30 min, washed with PBS, and transferred to a column. GST-fusion proteins were eluted with 50 mM Tris-HCl (pH 8.0) containing 10 mM glutathione, and peak fractions were pooled. Thrombin protease (Cytiva) was added to remove the GST tag, and the sample was incubated at 25 °C for 14 h. The digest was dialysed four times against 50 mM Tris-HCl (pH 7.5) containing 1 mM CaCl_2_, applied to a Glutathione Sepharose column, and flow-through fractions containing ColH were collected. For structural studies, fractions were concentrated by ultrafiltration with Amicon Ultra 30 K centrifugal filters (Millipore) and loaded onto a HiPrep Sephacryl S-200HR 16/60 column (Cytiva) pre-equilibrated with 50 mM Tris-HCl (pH 7.5), 150 mM NaCl, and 1 mM CaCl_2_.

### Purification of ColH^WT^ collagenase module

Purification of the ColH^WT^ CM followed the same procedure as for ColH^WT^, except that protein expression was induced at 30 °C for 6 h. After removal of the GST tag by affinity chromatography, proteins were purified by ion-exchange chromatography with Q-Sepharose Fast Flow beads (Cytiva) equilibrated with 50 mM Tris-HCl (pH 7.5) containing 1 mM CaCl_2_. The flow-through fraction containing ColH^WT^ CM was further purified by size-exclusion chromatography as described above.

### Cryo-EM sample preparation and data collection

Purified ColH^WT^ or mutant was diluted to 1.0 mg/mL in cryo-EM buffer (50 mM Tris-HCl, pH 7.5; 100 mM NaCl; 1 mM CaCl₂). ColH^MT^ was mixed with the synthetic peptide (POG)_10_, (PPG)_10_, or (POG)_12_ at a 1:2 molar ratio, adjusted to a final protein concentration of 1.0 mg/mL, and incubated on ice for 30 min. (POG)_10_ and (PPG)_10_ were purchased from Peptide Institute (Osaka, Japan), and (POG)_12_ from Bio-Synthesis (Lewisville, TX, USA), and were dissolved in cryo-EM buffer. Peptide solutions were heated at 95 °C for 5 min, cooled to room temperature for 10 min, and incubated at 4 °C for ≥24 h for triple-helix formation. Each sample was applied to glow-discharged copper grids (R1.2/1.3 200 mesh; Quantifoil, Großlöbichau, Germany) for 10 s at 10 mA using a JEC-3000FC Auto Fine Coater (JEOL). Grids were blotted for 3 s at 4 °C (100% humidity) with a blot force of 0 and plunge-frozen in liquid ethane using a Vitrobot Mark IV (Thermo Fisher Scientific, Waltham, MA, USA). Screening and data collection were performed on CRYO ARM 300 and CRYO ARM 300 II microscopes (JEOL) operating at 300 kV and equipped with a cold field-emission gun and K3 direct electron detector (Gatan, Pleasanton, CA, USA). Cryo-EM movies were recorded using SerialEM (version 4.1.6)^38^ with 5 × 5 beam-image shift patterns (coma vs. image shift calibrated before acquisition). The data collection parameters are listed in Supplementary Table 1.

### Cryo-EM data processing

All data processing was performed in CryoSPARC (version 4.4.1-4.6.0)^39^. Imported movies were subjected to beam-induced motion correction using Patch Motion Correction, and the contrast transfer function (CTF) parameters were estimated using Patch CTF estimation.

For ColH^WT^, 16,098,214 particles were automatically picked using Template Picker with templates generated from the ColH^WT^ CM crystal structure (PDB ID: 9CME) and extracted from 8,150 micrographs with a box size of 128 pixels (1.86 Å/pixel). After 2D classification to remove junk particles, the selected particles were subjected to several rounds of ab initio reconstruction and heterogeneous refinement. A total of 3,488,844 good-quality particles were re-extracted with a box size of 256 pixels at 0.87 Å/pixel, further classified, and refined to obtain 1,533,374 particles. Further homogeneous refinement, non-uniform refinement, and reference-based motion correction yielded 1,401,659 high-quality particles and a 2.3 Å consensus map. 3DVA^26^ was performed at 3 Å (Supplementary Fig. 5 and Supplementary Movies 1 and 2). Focused refinement of the AD and PD-PKD1 regions improved map quality. A composite map was generated using *phenix.combine_focused_maps* in PHENIX (version 1.19.2-4158 or 1.20.1-4487)^40^.

For the 1:1 ColH^MT^–(POG)_10_ complex [ColH^MT^ bound to the C-terminal region of (POG)_10_], 13,017,170 particles were picked with templates generated from the ColH^WT^ map and extracted from 7,349 micrographs with a box size of 120 pixels (1.74 Å/pixel). The extracted particles were subjected to several rounds of 2D classification, ab initio reconstruction, and heterogeneous refinement to filter junk particles. A total of 3,551,012 good-quality particles were re-extracted with a box size of 240 pixels (0.87 Å/pixel) and subjected to further rounds of 2D classification, ab-initio reconstruction, and heterogeneous refinement. After homogeneous refinement and subsequent non-uniform refinement, 534,057 particles were motion-corrected using reference-based motion correction to yield a cryo-EM map of 2.6 Å resolution. To exclude particles in which two ColH^MT^ molecules were bound to (POG)_10_, 503,128 particles were re-extracted with a box size of 400 pixels (0.87 Å/pixel) and subjected to 2D classification. A further 3D classification yielded three classes with ColH^MT^ bound to (POG)_10_ with a 1:1 stoichiometry and one class with bound ColH^MT^ to (POG)_10_ with a 2:1 stoichiometry. From the classes with only one ColH^MT^ bound, a total of 396,749 particles were re-extracted with a box size of 240 pixels (0.87 Å/pixel) and subjected to homogeneous refinement, non-uniform refinement, and reference-based motion correction. After homogeneous and non-uniform refinement, local refinement using 369,742 particles yielded a cryo-EM map of ColH^MT^ bound to the C-terminal region of (POG)_10_ at a 2.7 Å resolution.

For the 2:1 ColH^MT^–(POG)_10_ complex, 6,141,403 particles were picked with templates generated from the ColH^WT^ map, extracted from 7,385 micrographs with a box size of 120 pixels (2.32 Å/pixel), and subjected to several rounds of ab initio reconstruction and heterogeneous refinement to filter junk particles. A total of 745,629 good-quality particles were re-extracted with a box size of 300 pixels (0.87 Å/pixel) and subjected to several rounds of ab initio reconstruction and heterogeneous refinement. After homogeneous refinement and subsequent non-uniform refinement, 58,438 particles were subjected to reference-based motion correction. After homogeneous refinement, non-uniform refinement yielded a consensus map of the 2:1 ColH^MT^–(POG)_10_ complex at a 3.3 Å resolution. Using these particles, 3DVA^26^ was performed with a filter resolution of 5 Å (Supplementary Fig. 10, and Supplementary Movie 3). The quality of the map was improved by focused refinement of ColH^MT^ bound to the C- and N-terminal regions of (POG)_10_. A composite map of the 2:1 ColH^MT^–(POG)_10_ complex was generated using *phenix.combine_focused_maps* in PHENIX^40^.

For the ColH^MT^–(PPG)_10_ complex, 14,102,948 particles were automatically picked with templates generated from the ColH^WT^ cryo-EM map and extracted from 8,743 micrographs with a box size of 120 pixels (1.74 Å/pixel). The extracted particles were subjected to several rounds of 2D classification, ab initio reconstruction, and heterogeneous refinement to filter junk particles. A total of 3,773,237 good-quality particles were re-extracted with a box size of 240 pixels (0.87 Å/pixel) and subjected to several rounds of ab initio reconstruction and heterogeneous refinement. After homogeneous refinement and subsequent non-uniform refinement, 570,503 particles were subjected to reference-based motion correction. After homogeneous refinement and non-uniform refinement, local refinement using 569,950 particles yielded a cryo-EM map of the 1:1 ColH^MT^–(PPG)_10_ complex with a 2.2 Å resolution.

For the ColH^MT^–(POG)_12_ complex, 10,114,389 particles were automatically picked using Template Picker with templates generated from the cryo-EM map of ColH^MT^ bound to the C-terminal of (POG)_10_ and extracted from 6,875 micrographs with a box size of 120 pixels (1.72 Å/pixel). The extracted particles were subjected to several rounds of ab initio reconstruction, heterogeneous refinement, and 2D classification to filter junk particles. A total of 2,069,705 good-quality particles were re-extracted with a box size of 240 pixels (0.86 Å/pixel) and subjected to several rounds of ab initio reconstruction and heterogeneous refinement. Further 3D classification yielded one class with a clear map for the AD Exo^Lead^ and Exo^Middle^ regions. After homogeneous and non-uniform refinement, 318,280 particles were subjected to reference-based motion correction. After homogeneous refinement, non-uniform refinement yielded a consensus map of the ColH^MT^–(POG)_12_ complex at a 2.8 Å resolution. The quality of the map was improved by focused refinement of the AD and PD-PKD1 regions. A composite map of the ColH^MT^–(POG)_12_ complex was generated using *phenix.combine_focused_maps* in PHENIX^40^.

The global resolution of each cryo‑EM map was estimated using the gold‑standard Fourier shell correlation (FSC) between independently refined half‑maps with the 0.143 criterion. The resolution of the atomic model was defined as that at the FSC = 0.5 cutoff in the model‑to‑map FSC curve. For composite maps, the composite half‑maps produced using *phenix.combine_focused_maps* were imported into CryoSPARC^39^ and processed with the Validation (FSC) job to generate gold‑standard map‑to‑map FSC curves. The global resolution of each composite map was defined at the FSC = 0.143 threshold. These model-to-map and map-to-map FSC curves and corresponding resolution values are summarised in Supplementary Table 1 and shown in Supplementary Figs. 1, 8, 9, 14, and 20.

### Cryo-EM model building and refinement

For ColH^WT^, an initial model was built with ModelAngelo^41^ based on the ColH amino acid sequence, manually refined in Coot (version 0.8.9.2 or 0.9.8.93 EL)^42^, and subjected to real-space refinement using *phenix.real_space_refine* in PHENIX^40^. Water molecules were automatically placed in Coot^42^ and manually verified. The final model comprised residues Gln2–Asp770.

For the 1:1 ColH^MT^–(POG)_10_ and 1:1 ColH^MT^–(PPG)_10_ complexes, initial models were built in ModelAngelo^41^ with sequences of ColH and the respective peptides. Manual refinement was performed in Coot^42^ and real-space refinement in PHENIX (using *phenix.real_space_refine*)^40^. Water molecules were automatically located using Coot^42^ and manually verified. Each final model contained three collagen peptide segments (Pro16–Gly30 of the leading, middle, and trailing chains) bound to ColH^MT^.

For the 2:1 ColH^MT^–(POG)_10_ complex, initial models were generated by superimposing the 1:1 ColH^MT^–(POG)_10_ and ColH^WT^ structures onto the composite map in ChimeraX (version 1.9-1.10.1)^43^. After manual and real-space refinements, the final model was generated with one ColH^MT^ bound to each (POG)_10_ terminus.

For the ColH^MT^–(POG)_12_ complex, the 1:1 ColH^MT^–(POG)_10_ structure was fit into the map in ChimeraX^43^. Non-visible peptide regions were removed, followed by refinement in Coot^42^ and PHENIX^40^ (using *phenix.real_space_refine*). The final model included two (POG)_12_ peptide segments (Gly-Pro-Hyp-Gly-Pro-Hyp-Gly) bound to Exo^Lead^ and Exo^Middle^, respectively, and one additional segment (Gly-Pro-Hyp-Gly-Pro-Hyp-Gly-Pro-Hyp-Gly) at the active site.

All structures were validated with MolProbity^44^ in PHENIX^40^. The refinement statistics are provided in Supplementary Table 1.

### Crystallisation

High-throughput screening using a Hampton Research HR2-144 index on an Oryx 8 (Douglas Instruments) identified several crystallisation conditions. ColH^WT^ CM at 7.7 mg/mL in 50 mM Tris-HCl (pH 7.5), 50 mM NaCl, and 1 mM CaCl₂ produced crystals within 24–48 h. Optimal conditions were 0.1 M Bis-Tris (pH 6.5), 0.2 M magnesium chloride hexahydrate, and 25% (w/v) polyethylene glycol (PEG) 3350, supplemented with Izit Crystal Dye (Hampton Research) for visualisation. Microseeds were prepared following the protocol of D’Arcy et al.^45^, adapted from Luft and DeTitta^46^. Briefly, 20–50 µL of reservoir solution was chilled on ice in a seed bead tube, and the crystals were crushed for 30 s and rinsed in cold reservoir solution; this process was repeated until no visible crystals remained. The seed stock was vortexed four times for 30 s and stored at –80 °C.

### X-ray crystallography and structure analysis

Tetragonal crystals grown using the sitting-drop method with microseeding were mounted in sealed quartz capillaries. Diffraction data were collected at room temperature on a Rigaku XtaLAB Synergy-S equipped with a copper sealed-tube X-ray source and HyPix600HE detector. A crystal from a reservoir containing 1.0 M MgCl₂·6H₂O, 1.0 M Tris-HCl (pH 8.5), and 50% (w/v) PEG 3350 was selected. Data were processed using *CrysAlisPro* (Rigaku Oxford Diffraction) and *Aimless* from the CCP4 suite (version 8.0.019)^47^. Phases were determined by molecular replacement using *Phaser* in PHENIX^40^ with the ColH PD segment (PDB ID: 4AR1) and ColG AD (PDB ID: 4ARE) as search models. Iterative rounds of manual rebuilding were performed in Coot^42^, real-space refinement in PHENIX^40^, and reciprocal-space refinement with twin correction (–h, l, k) in *REFMAC5* from the CCP4 suite^47^. The refined structure showed 95.11% of residues in favoured regions and 4.89% in allowed regions and no Ramachandran outliers. The data collection and refinement statistics are summarised in Supplementary Table 2.

### Enzyme assays

Protein concentrations were determined with a Pierce BCA Protein Assay Kit (Thermo Fisher Scientific, Rockford, IL, USA) using bovine serum albumin as a standard. Hydrolytic activity toward a non-helical collagen peptide was measured with Pz-peptide (Sigma-Aldrich, St. Louis, MO, USA) as described by Wünsch and Heidrich^48^. Collagenolytic activity against insoluble bovine Achilles tendon (code CL; Worthington Biochemical, Freehold, NJ, USA) was measured following the manufacturer’s instructions. Briefly, 25 mg of collagen was swollen in 5 mL of 50 mM Tris(hydroxymethyl)-methyl-2-aminoethane sulfonate and 0.36 mM CaCl_2_ (pH 7.5) for 30 min at 37 °C. Reactions were initiated by adding 0.1 mL enzyme solution containing 10 µg protein and incubated for 5 h at 37 °C. The supernatant was collected by centrifugation at 4 °C, and liberated amino acids were quantified using ninhydrin assays, expressed as leucine equivalents. All assays were performed in triplicate across three independent experiments. To compare the statistical differences between wild-type and mutant ColH, a two-tailed *t*-test was used (Fig. 2f).

### Digestion assays of triple-helical peptides

(POG)_10_ and (PPG)_10_ peptides were dissolved in water (1 mg/mL), heated at 95 °C for 5 min, cooled to room temperature for 10 min, and incubated at 4 °C for ≥24 h to allow triple-helix formation. Digestion reactions with ColH^WT^ were performed in 50 mM Tris-HCl (pH 7.5), 100 mM NaCl, and 1 mM CaCl₂. (POG)_10_ (0.1 mg/mL) was incubated with ColH^WT^ (0.01 mg/mL) at 4 °C for 1, 9, or 24 h, or at 37 °C for 1 or 3 h. At a higher enzyme concentration (0.1 mg/mL), reactions were conducted at 4 °C for 1, 3, or 24 h. For (PPG)_10_ (0.1 mg/mL), digestion using 0.01 mg/mL ColH^WT^ was conducted at 4 °C for 1 or 2 h.

At each time point, aliquots were analysed by reversed-phase high-performance liquid chromatography on a COSMOSIL 5C18-AR-II column (4.6 × 250 mm; Nacalai Tesque, Kyoto, Japan) at 60 °C, with UV detection at 220 nm. Separations were run at 1 mL/min with linear acetonitrile gradients (10–40% or 0–30% over 30 min) in water, both containing 0.05% trifluoroacetic acid. Peaks corresponding to intact peptides were integrated, and the percentage of residual substrate was calculated relative to the undigested controls.

### Sequence alignment

Amino-acid sequences from three previously determined structures and one newly determined structure—two M9A collagenases (Ghcol^21^ and VhaC^12^) and two M9B collagenases (ColG^11^ and ColH)—were aligned using Clustal Omega (version 1.2.4)^49^. Structural alignment of their AD and catalytic subdomain was performed in Coot^42^ to identify structurally equivalent residues and refine the sequence alignment. Secondary-structure elements for ColH were assigned using DSSP (version 4)^50^.

### Circular dichroism (CD) spectroscopy

Far-UV CD spectra were recorded using a Jasco J-720 spectropolarimeter (JASCO, Tokyo, Japan) to assess the secondary structure of the purified proteins. Protein samples were prepared in compatible buffer (50 mM Tris-HCl, pH 7.5, 100 mM NaCl, 1 mM CaCl₂) at a final concentration of ~0.5 mg/mL. CD measurements were performed at 25 °C using a synthetic quartz cuvette with a path length of 1 mm. Spectra were collected over a wavelength range of 200–250 nm with a bandwidth of 1 nm, a scanning speed of 200 nm/min, and a response time of 0.5 s. A buffer spectrum recorded under identical conditions was subtracted from each protein spectrum.

### Structure modelling

The structures of ColH^WT^ and Pz-peptide complexes were modelled using ColH^MT^-(POG)_10_ (PDB ID: 9LQJ) as the initial model. In ColH^MT^-(POG)_10_, three collagen-like peptides form a complex with ColH, and the two collagen-like peptides bound to the AD were removed. For the remaining collagen-like peptides bound to the PD, only the region around the active site was retained and residue Q416 was mutated back to E416. Based on the remaining collagen-like peptide, we modelled the Pz-peptide in Molecular Operating Environment (MOE) software (ver. 2020, Chemical Computing Group, Inc.)^51^. The protonation in ColH^WT^ and Pz-peptide complexes was performed using Protonate 3D in MOE and optimisation considered the ligand to surrounding 4.5 Å amino acid residues with a constraint (tether = 1.0) under the AMBER10: EHT force field^52,53^.

### Reporting summary

Further information on research design is available in the Nature Portfolio Reporting Summary linked to this article.

## Supplementary information

Supplementary Information

Description of Additional Supplementary Files

Supplementary Movie 1

Supplementary Movie 2

Supplementary Movie 3

Reporting Summary

Transparent Peer Review file

## Source data

Source Data

## Data Availability

The cryo-electron microscopy maps have been deposited to the Electron Microscopy Data Bank under accession codes EMD-63331 (consensus map of ColH^WT^), EMD-63333 (focused map for the AD region of ColH^WT^), EMD-63332 (focused map for the PD–PKD1 region of ColH^WT^), EMD-63337 (composite map of ColH^WT^), EMD-63297 (consensus map of 1:1 ColH^MT^–(POG)_10_ complex), EMD-63334 (consensus map of 2:1 ColH^MT^–(POG)_10_ complex), EMD-63336 (focused map for the ColH^MT^-N region of 2:1 ColH^MT^–(POG)_10_ complex), EMD-63335 (focused map for the ColH^MT^-C region of 2:1 ColH^MT^–(POG)_10_ complex), EMD-63339 (composite map of 2:1 ColH^MT^–(POG)_10_ complex), EMD-65889 (consensus map of 1:1 ColH^MT^–(PPG)_10_ complex), EMD-63509 (focused map for the AD region of ColH^MT^–(POG)_12_ complex), EMD-63510 (focused map for the PD-PKD1 region of ColH^MT^–(POG)_12_ complex), EMD-63508 (consensus map of ColH^MT^–(POG)_12_ complex), and EMD-63511 (composite map of ColH^MT^–(POG)_12_ complex). The corresponding atomic coordinates have been deposited to the Protein Data Bank under accession codes 9LRK (ColH^WT^), 9LQJ (1:1 ColH^MT^–(POG)_10_ complex), 9LRM (2:1 ColH^MT^–(POG)_10_ complex), 9WDC (1:1 ColH^MT^–(PPG)_10_ complex), and 9LYI (ColH^MT^–(POG)_12_ complex). The atomic coordinates and structure factors from the X-ray crystallographic analysis of ColH^WT^ CM have been deposited in the PDB with accession code 9CME. PDB codes of previously published structures used in this study are 4AR1, 4ARE and 3B0S. Source data are provided with this paper.
