# Supplementary material for: Bacterial collagenase harnesses collagen geometry for processive cleavage
Source: Nat Commun. 2026 Apr 2;17:5485. doi: 10.1038/s41467-026-71099-3 (PMC13284217; doi:10.1038/s41467-026-71099-3)
Supplement: Supplementary file 1 — Supplementary Information [file 41467_2026_71099_MOESM1_ESM.pdf]

Supplementary Information

Bacterial Collagenase Harnesses Collagen Geometry for Processive Cleavage

Hiroya Oki[1], Katsuki Takebe[2, 3], Adjoa Bonsu[4], Kazunori Fujii[5], Ryo Masuda[6], Nicholas Henderson[4], Takehiko Mima[7], Takaki Koide[5, 6], Mahmoud Moradi[4], Osamu Matsushita[8]*, Joshua Sakon[4]*, Kazuki Kawahara[3, 9]*

[1]Department of Infection Metagenomics, Genome Information Research Center, Research Institute for Microbial Diseases, The University of Osaka, Osaka 565-0871, Japan

[2]Department of Dental Pharmacology, Graduate School of Medicine, Dentistry and Pharmaceutical Sciences, Okayama University, Okayama 700-8558, Japan

[3]Graduate School of Pharmaceutical Sciences, The University of Osaka, Osaka 565-0871, Japan

[4]Department of Chemistry and Biochemistry, University of Arkansas, Fayetteville, AR 72701, USA

[5]Department of Chemistry and Biochemistry, School of Advanced Science and Engineering, Waseda University, Shinjuku-ku, Tokyo 169-8555, Japan

[6]Waseda Research Institute for Science and Engineering, Waseda University, Shinjuku, Tokyo 169-8555, Japan

[7]Department of Medical Technology, Faculty of Health Sciences, Ehime Prefectural University of Health Sciences, Ehime 791-2101, Japan

[8]Department of Bacteriology, Graduate School of Medicine, Dentistry, and Pharmaceutical Sciences, Okayama University, Okayama 700-8558, Japan

[9]Center for Infectious Disease Education and Research, The University of Osaka, Osaka 565-0871, Japan

*Corresponding authors: osamu@okayama-u.ac.jp (OM), jsakon@uark.edu (JS), and kkkazuki@phs.osaka-u.ac.jp (KK)

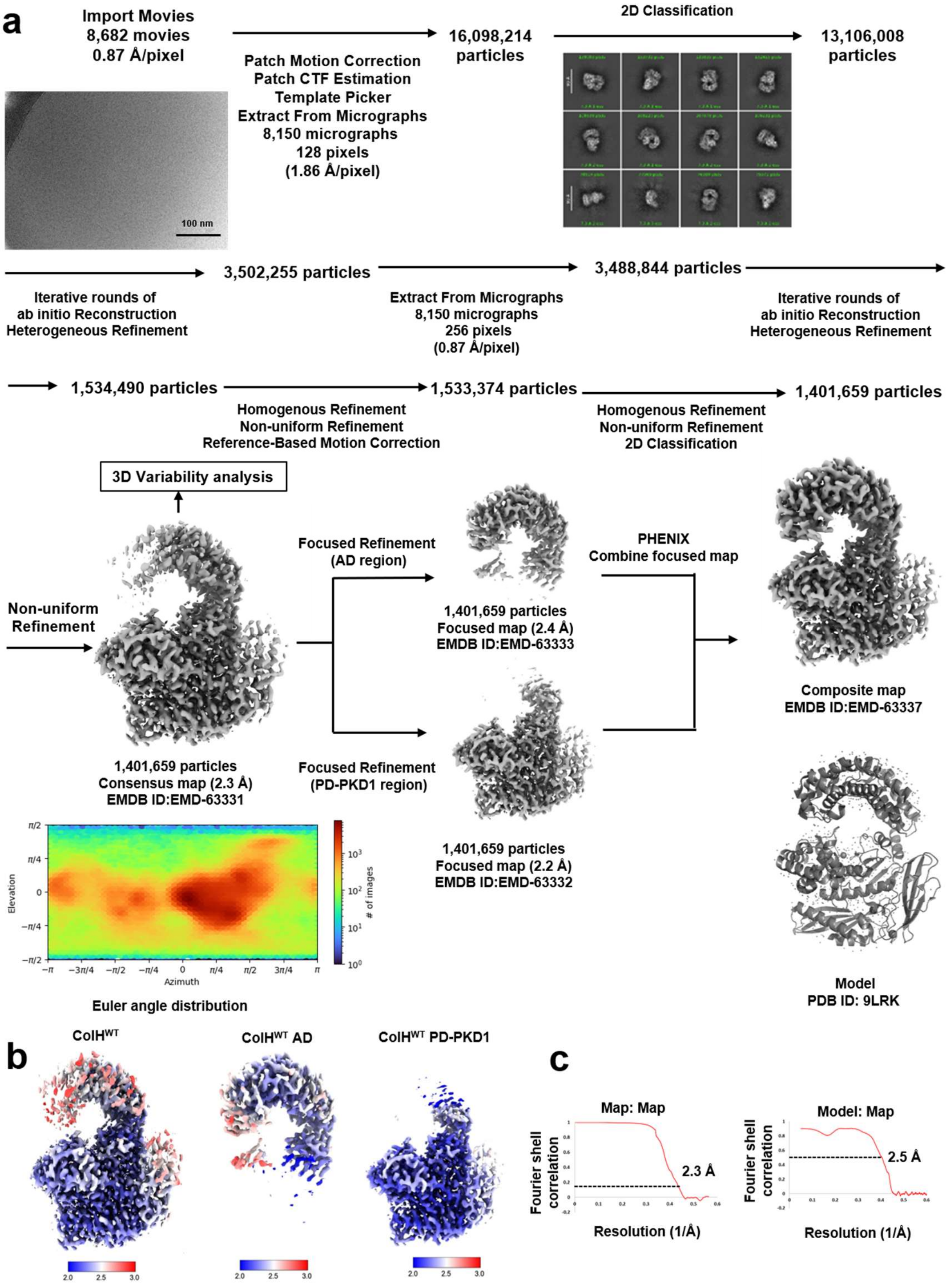

Supplementary Fig. 1. Cryo-electron microscopy (Cryo-EM) of ColH^{WT}.

a Cryo-EM data processing workflow for ColH^{WT}, performed using CryoSPARC[39]. A representative micrograph, 2D class averages, a 3D reconstruction, consensus cryo-EM density map, focused cryo-EM density maps, composite cryo-EM density map, and Euler angle distribution plot are shown, along with the atomic model of ColH^{WT}. The particle set used for calculating the consensus map was further subjected to 3DVA[26], as described in Supplementary Fig. 5, and illustrated in Supplementary Movies 1 and 2. **b** Cryo-EM density maps of ColH^{WT}. Consensus cryo-EM density map of ColH^{WT} (left panel). Focused cryo-EM density map of the activator domain (AD) region of ColH^{WT} (middle panel). Focused cryo-EM density map of the peptidase domain–polycystic kidney disease-like domain 1 (PD–PKD1) region of ColH^{WT} (right panel). The density maps are coloured according to local resolution. **c** Gold-standard map-to-map and model-to-map FSC curves. The intersections with FSC = 0.143 (map-to-map) and FSC = 0.5 (model-to-map) are indicated, along with the corresponding resolutions.

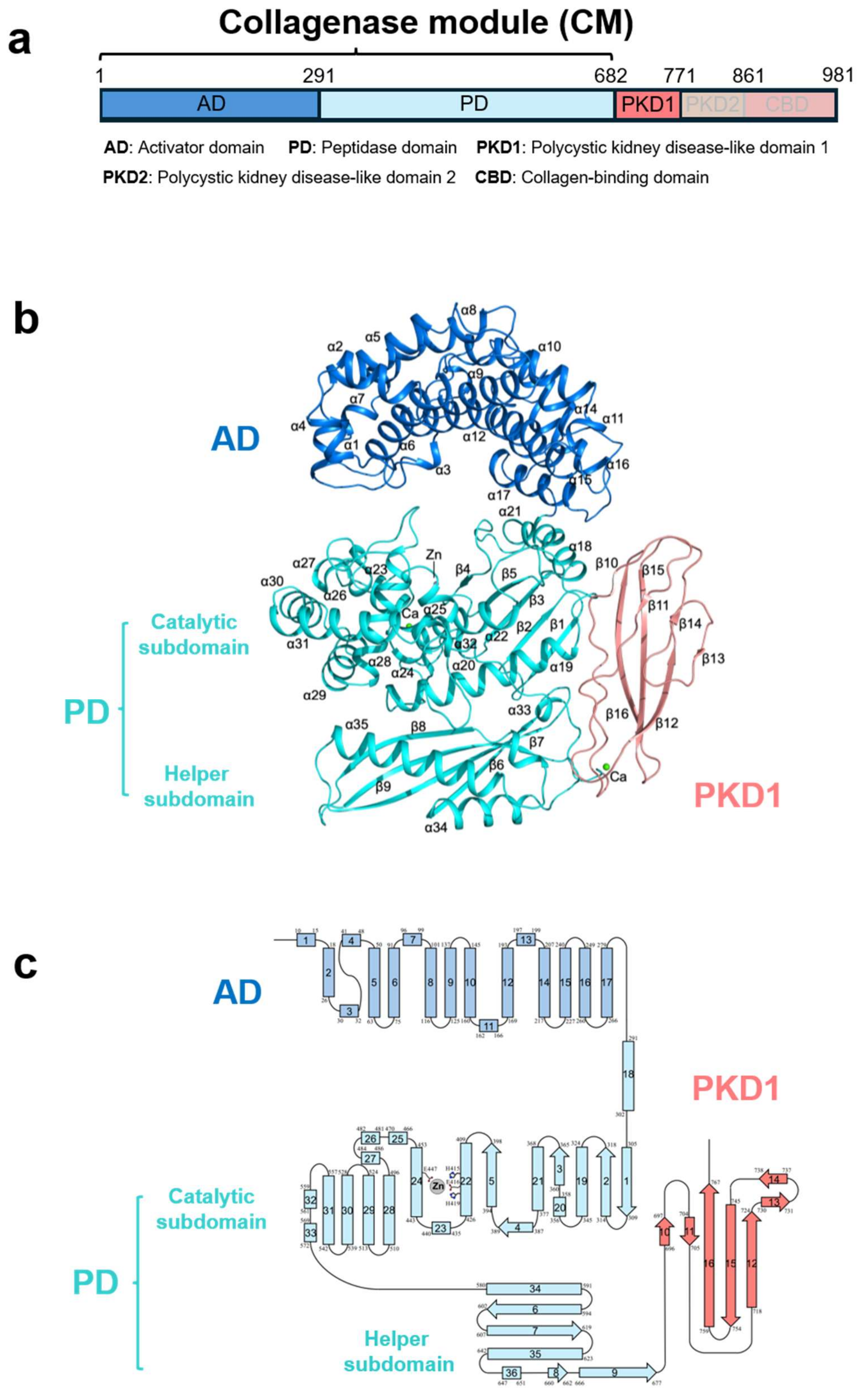

Supplementary Fig. 2. Cryo-EM structure of ColH^{WT}.

a Domain organisation of ColH^{WT}. Each domain is coloured using the same scheme as in Fig. 1a. The polycystic kidney disease-like domain 2 (PKD2) and collagen-binding domain (CBD), which were not resolved in the present analysis, are shaded. **b** Cryo-EM structure of ColH^{WT} with assigned secondary structural elements. Domains are coloured according to the scheme shown in Fig. 1d. **c** Topology diagram of cryo-EM structure for ColH^{WT}. Rectangles represent α-helices and arrows represent β-sheets.

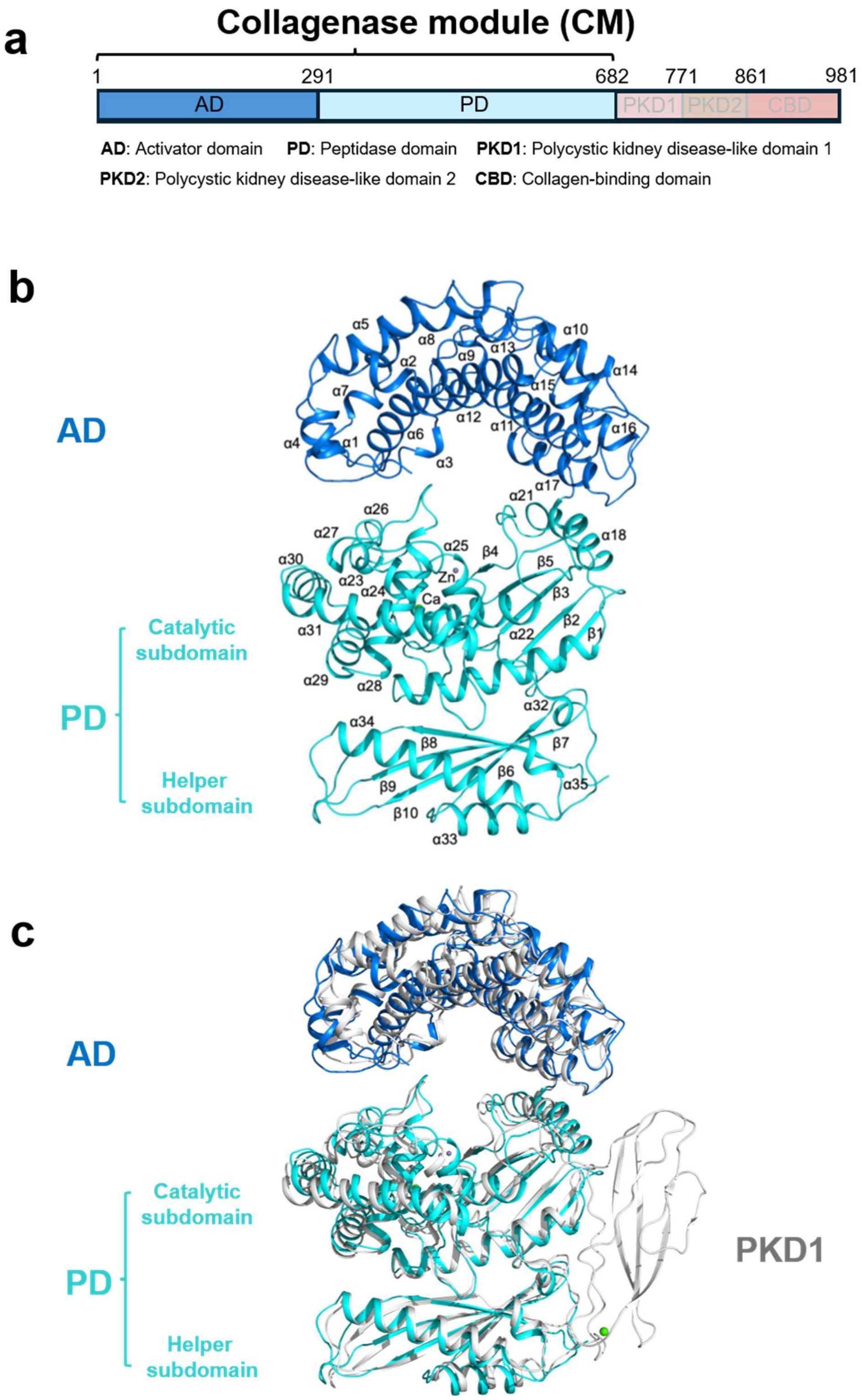

Supplementary Fig. 3. Room-temperature crystal structure of ColH^{WT} CM.
a Domain organisation of ColH^{WT}. Each domain is coloured using the same scheme as in Fig. 1a. PKD1, PKD2, and CBD, which were truncated in the present analysis, are shaded. **b** Crystal structure of the ColH^{WT} CM determined at room temperature, with assigned secondary structural elements. Domains are coloured according to the scheme shown in Fig. 1d. A zinc ion is shown as a grey sphere. **c** Structural superimposition of the ColH^{WT} CM crystal structure (coloured as in Supplementary Fig. 3b) and ColH^{WT} cryo-EM structure (light grey), based on all Cα atoms within the CM.

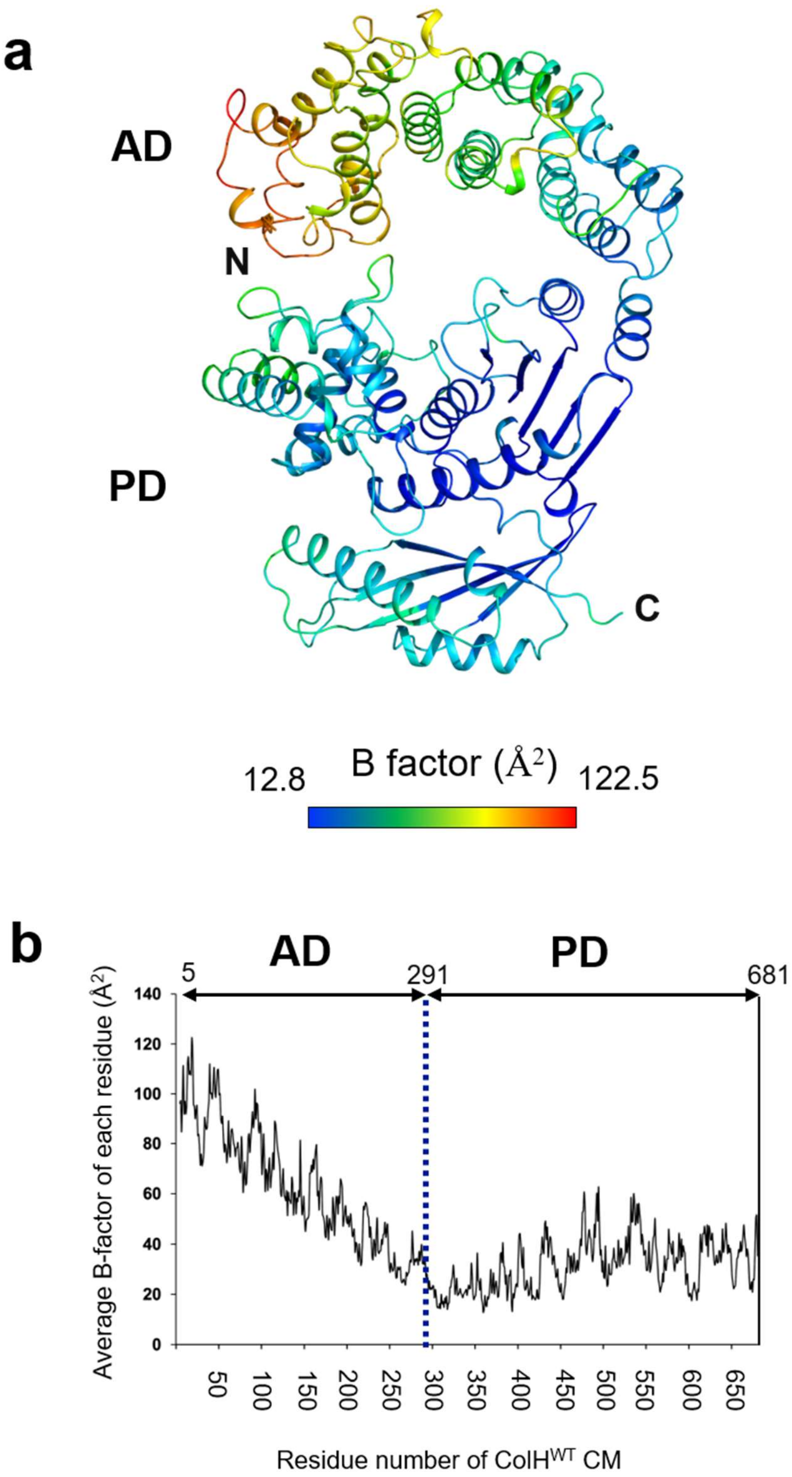

Supplementary Fig. 4. Graphical representation of ColH^{WT} CM flexibility.
a Colour mapping of B-factors on the crystal structure of ColH^{WT} CM. The data are shown using a colour scale from 12.75 Å^2 (blue) to 122.46 Å^2 (red). **b** Plotting of B-factors along the residue number. In the AD, B-factors increased linearly toward the N-terminus. Source data are provided as a Source Data file.

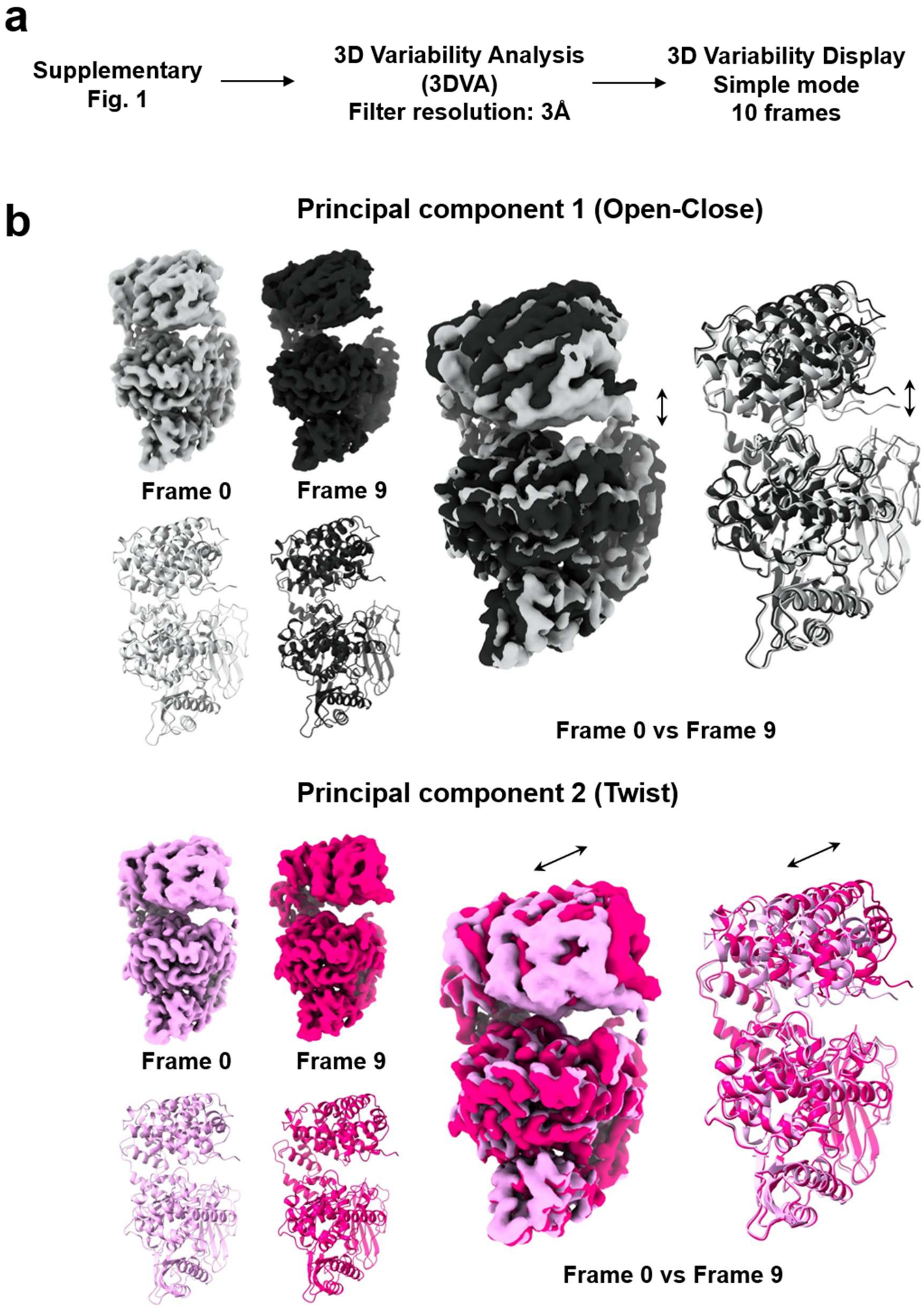

Supplementary Fig. 5. 3DVA of ColH^{WT}.

a 3DVA of ColH^{WT} was performed using the particle set in Supplementary Fig. 1 with a 3 Å filter resolution in CryoSPARC[39]. **b** EM maps for the first and last frames (0 and 9), fitted models, and their superimpositions for principal components 1 (top panel) and 2 (bottom panel). Arrows indicate the directions of the principal motions, corresponding to partial opening/closing of the AD relative to PD-PKD1 for component 1 and a twisting motion of the AD relative to PD-PKD1 for component 2. See also Supplementary Movies 1 and 2.

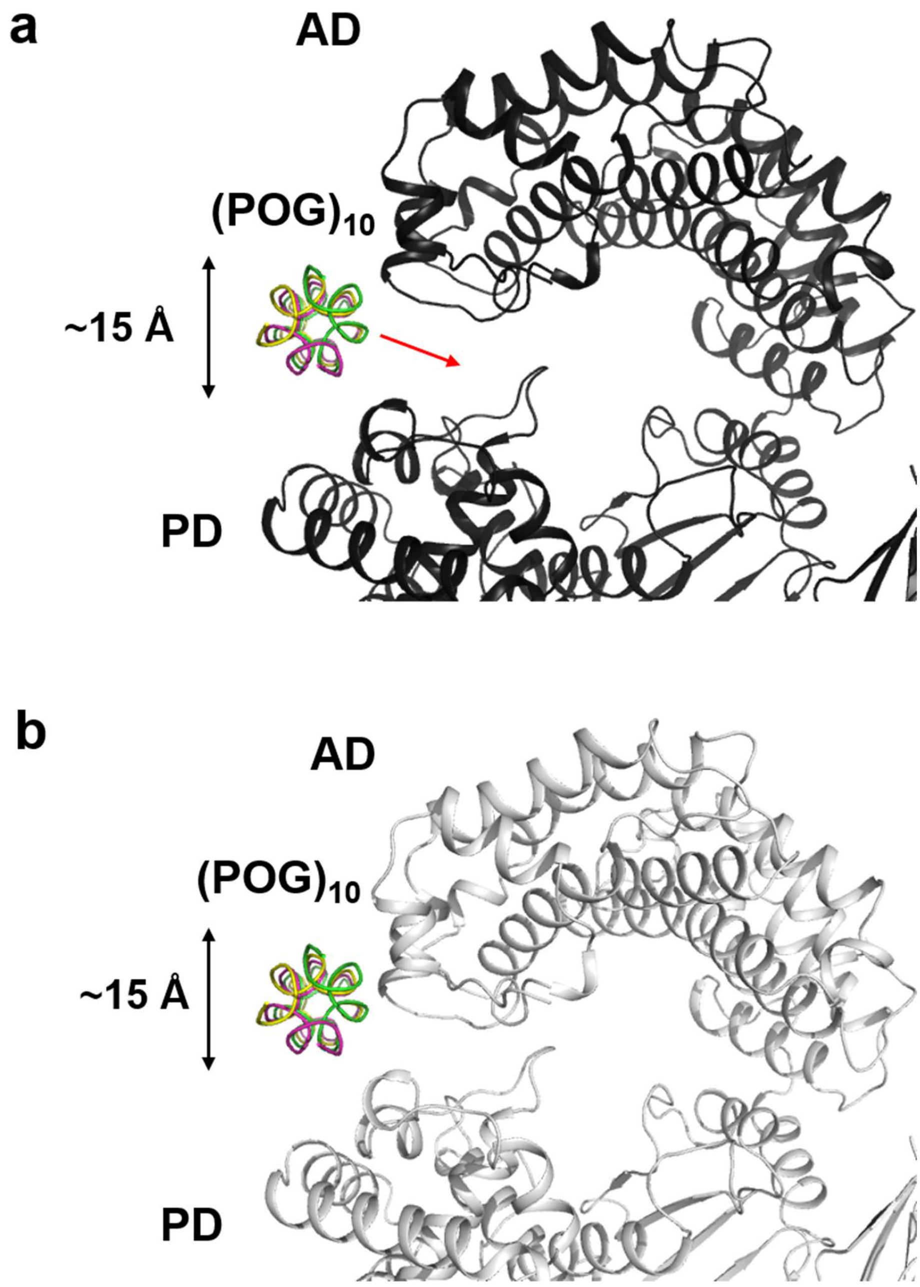

Supplementary Fig. 6. Partial opening of ColH^{WT} permits triple helix encirclement.
a An enlarged view of the gate region in the 'open' ColH^{WT} structure (frame 9 of 3DVA component 1). **b** An enlarged view of the gate region in the 'closed' ColH^{WT} structure (frame 0 of 3DVA component 1). In both cases, a triple-helical collagen model peptide, $(POG)_{10}$, is positioned next to the gate region to represent tropocollagen.

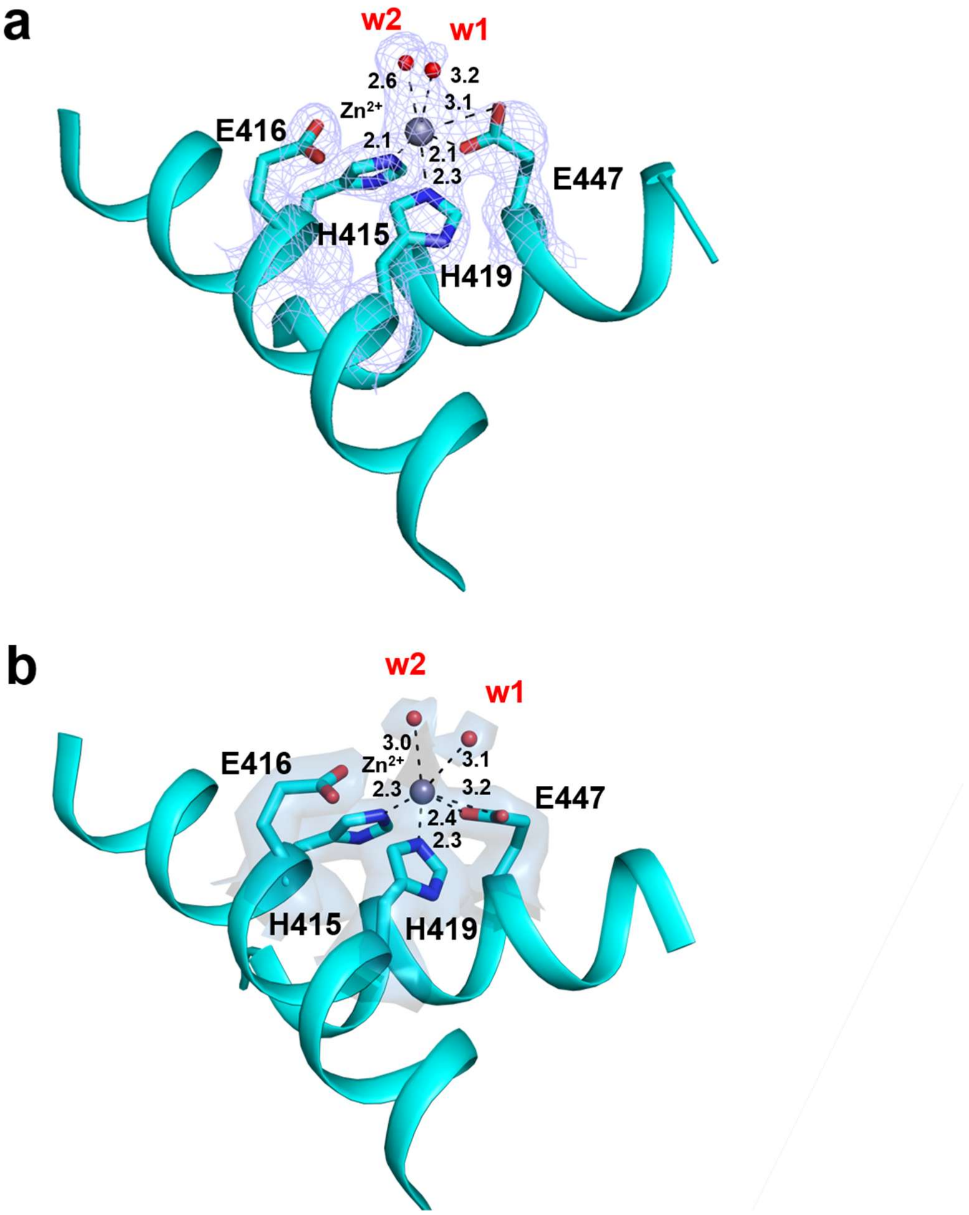

Supplementary Fig. 7. Comparison of the catalytic centre of ColH^{WT} CM in the crystal and cryo-EM structures.

a Amino acid residues (His415, His419, and Glu447) and water molecules (w1 and w2) coordinating with the Zn^{2+} ion in the catalytic centre of ColH^{WT} CM in the crystal structure. 2Fo-Fc electron density maps (contoured at 1.0 σ) are coloured light blue. **b** Amino acid residues (His415, His419, and Glu447) and water molecules (w1 and w2) coordinating with the Zn^{2+} ion in the catalytic centre of ColH^{WT} CM in the cryo-EM structure. The cryo-EM map is contoured at 5.0 σ and coloured light blue. The catalytic base Glu416 is also illustrated. All distances are shown in Å.

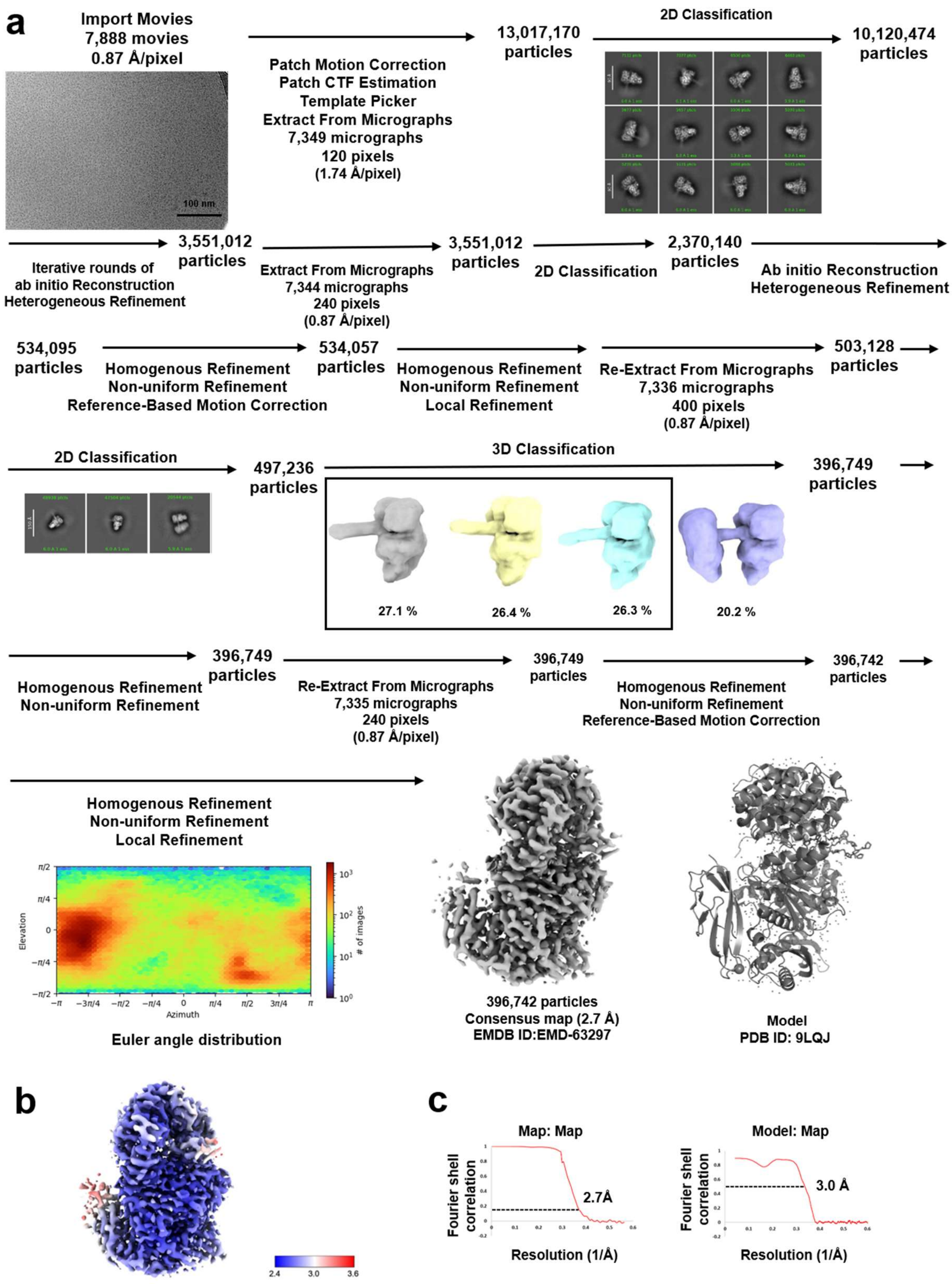

Supplementary Fig. 8. Cryo-EM of 1:1 ColH^{MT}–(POG)$_{10}$ complex.
a Cryo-EM data processing workflow for 1:1 ColH^{MT}–(POG)$_{10}$ complex. Cryo-EM data processing was performed using CryoSPARC[39]. A representative micrograph, 2D class averages, a 3D reconstruction, consensus cryo-EM density map, and Euler angle distribution plot are shown, along with the atomic model of the 1:1 ColH^{MT}–(POG)$_{10}$ complex. **b** Cryo-EM density map of 1:1 ColH^{MT}–(POG)$_{10}$ complex, coloured according to local resolution. **c** Gold-standard map-to-map and model-to-map FSC curves. The intersections with FSC = 0.143 (map-to-map) and FSC = 0.5 (model-to-map) are indicated, along with the corresponding resolutions.

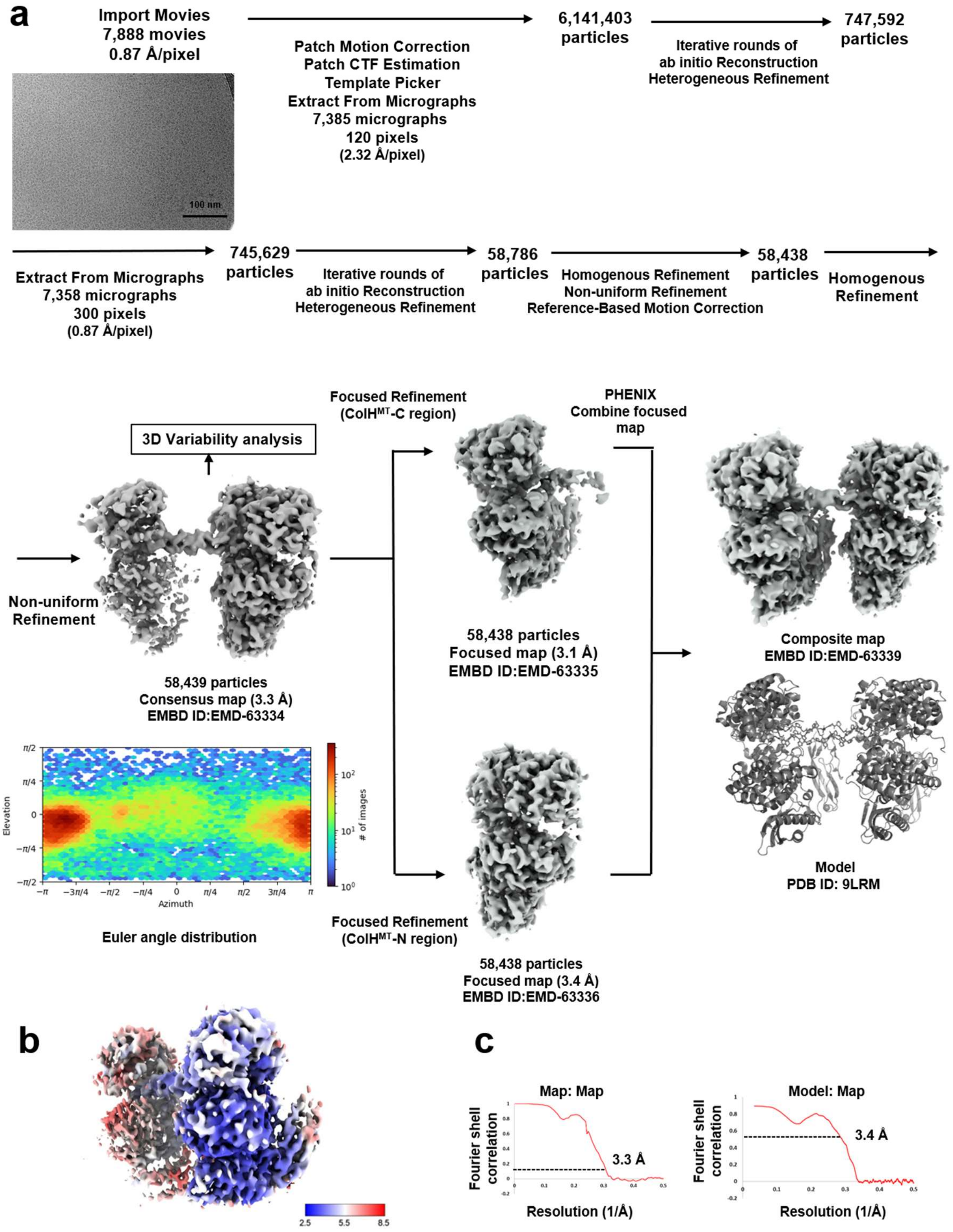

Supplementary Fig. 9. Cryo-EM of 2:1 ColH^{MT}–(POG)$_{10}$ complex.
a Cryo-EM data processing workflow for 2:1 ColH^{MT}–(POG)$_{10}$ complex. Cryo-EM data processing was performed using CryoSPARC[39]. A representative micrograph, 2D class averages, a 3D reconstruction, consensus cryo-EM density map, focused cryo-EM density maps, composite cryo-EM density map, and Euler angle distribution plot are shown, along with the atomic model of the 2:1 ColH^{MT}–(POG)$_{10}$ complex. The particle set used for calculating the consensus map was further subjected to 3DVA[26], as described in Supplementary Fig. 10 and illustrated in Supplementary Movie 3. **b** Consensus cryo-EM density map of 2:1 ColH^{MT}–(POG)$_{10}$ complex, coloured per local resolution. **c** Gold-standard map-to-map and model-to-map FSC curves. The intersections with FSC = 0.143 (map-to-map) and FSC = 0.5 (model-to-map) are indicated, along with the corresponding resolutions.

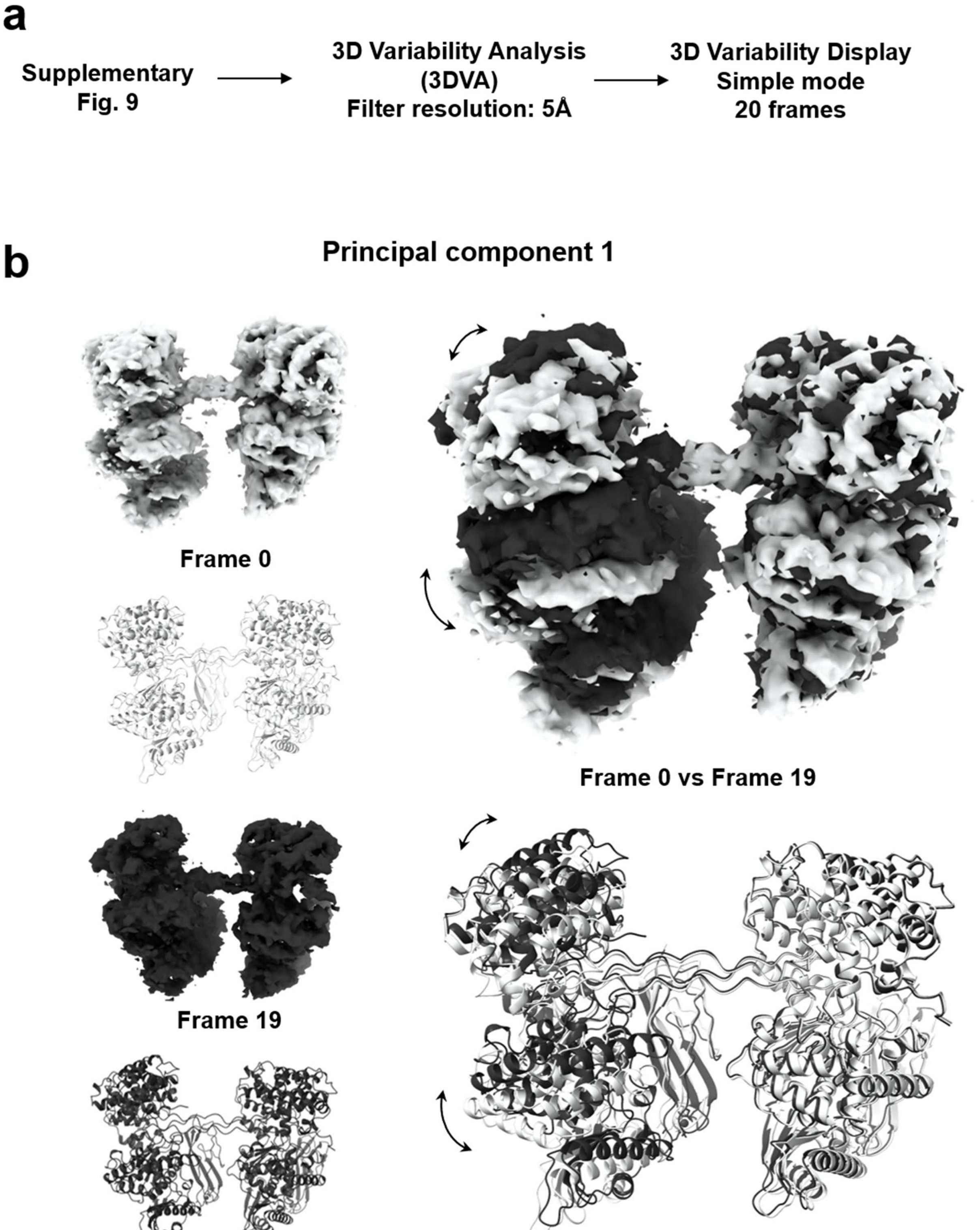

Supplementary Fig. 10. 3DVA of 2:1 ColH^{MT}–(POG)$_{10}$ complex.
a 3DVA was performed using the particle set described in Supplementary Fig. 9 with a 5 Å filter resolution in CryoSPARC[39]. **b** EM maps for first and last frames (frames 0 and 19), fitted models, and their superimpositions are displayed for principal component 1. Arrows indicate the directions of the principal motions. The ColH^{MT} molecule bound at the (POG)$_{10}$ N-terminus (ColH^{MT}-N) exhibits higher conformational flexibility than the one bound at the (POG)$_{10}$ C-terminus (ColH^{MT}-C). See also Supplementary Movie 3.

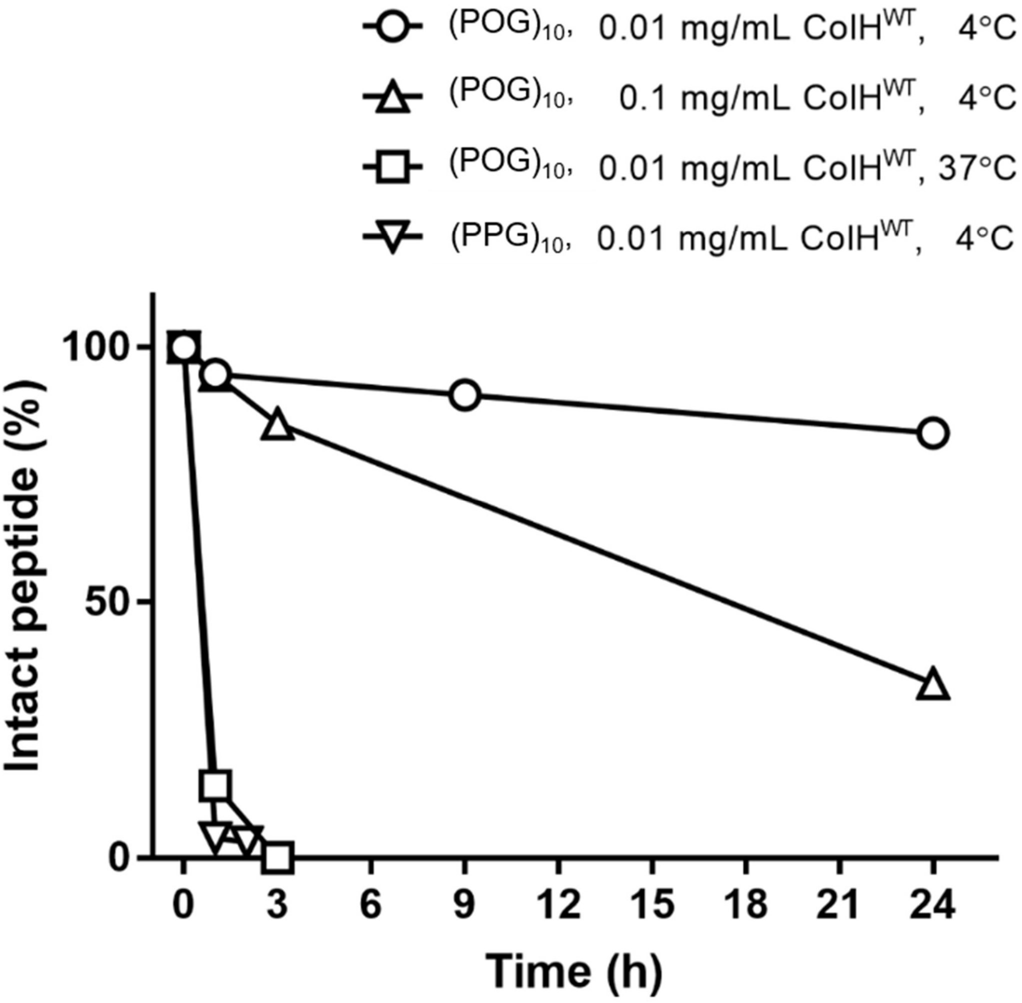

Supplementary Fig. 11. Digestion of triple-helical peptides by ColH^{WT}.

The percentages of intact $(POG)_{10}$ and $(PPG)_{10}$ remaining were determined under four reaction conditions varying in peptide substrate, ColH^{WT} concentration, and incubation temperature, with the initial peptide concentration fixed at 0.1 mg/mL. Reaction mixtures were analysed using reversed-phase high-performance liquid chromatography, and the percentage of intact peptide was calculated relative to the peak area of the undigested control. The experiment was performed once (n = 1) to demonstrate a qualitative observation supporting the experimental rationale for using the protease-null ColH mutant. Source data are provided as a Source Data file.

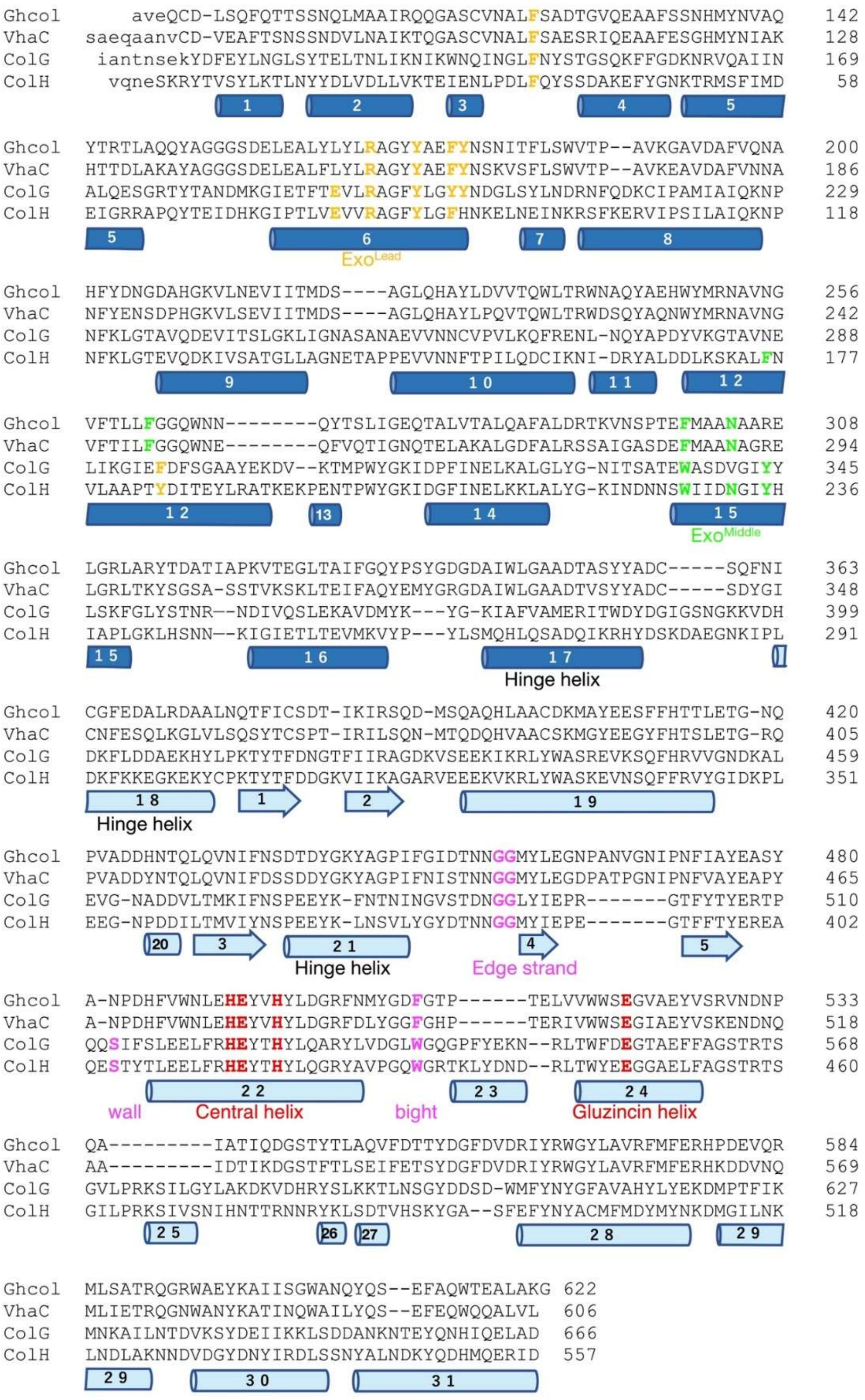

Supplementary Fig. 12. Amino acid sequence alignment among mature bacterial collagenases.
Sequences of AD and PD (or catalytic subdomain) from *Grimontia hollisae* (M9A, Ghcol, GenBank BAK39964), *Vibrio harveyi* (M9A, VhaC, GenBank WP_047516938), and *Hathewaya histolytica* (M9B, ColG, GenBank BAA77453; and M9B, ColH, GenBank BAA06251) were aligned, and the gaps ('-') were manually adjusted by structural comparison. N-terminal residues in lower case were not observed. Substrate-binding residues for the leading, middle, and trailing strands are coloured yellow, green, and magenta, respectively. Residues at the catalytic centre are coloured red. Secondary structures of ColH are illustrated by cylinders and arrows for helices and sheets, respectively. The colouring is the same as the structural illustration in Fig. 1d.

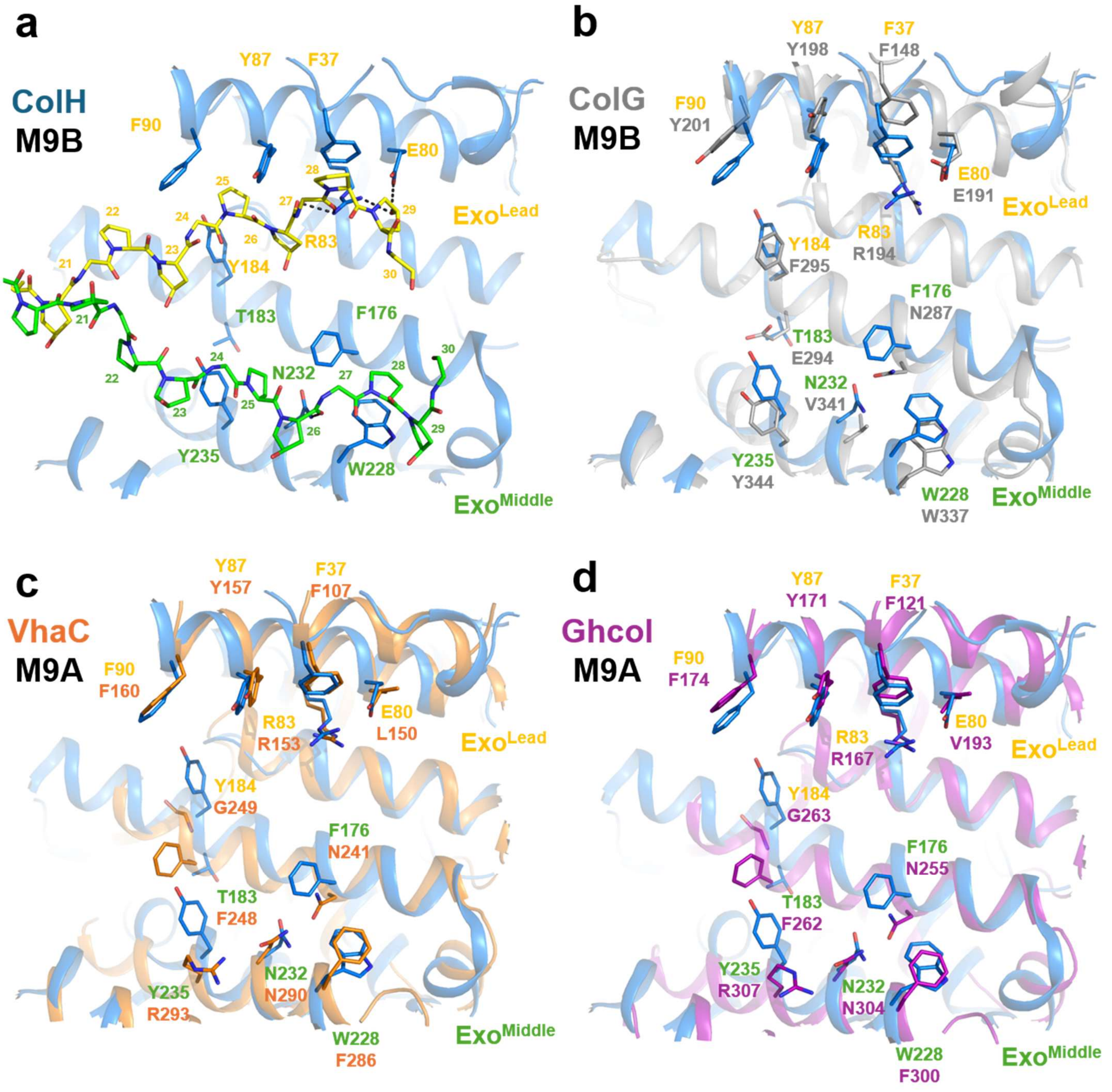

Supplementary Fig. 13. Structural comparison of the AD exosites of M9 family collagenases.
a Magnified view of Exo^{Lead} and Exo^{Middle} of ColH^{MT}–(POG)$_{10}$ complex from *H. histolytica* (PDB ID: 9LQJ, this study). **b** Magnified view of Exo^{Lead} and Exo^{Middle} of ColG from *H. histolytica* (PDB ID: 2Y3U). **c** Magnified view of Exo^{Lead} and Exo^{Middle} of VhaC from *V. harveyi* (PDB ID: 7ESI). **d** Magnified view of Exo^{Lead} and Exo^{Middle} of Ghcol from *G. hollisae* (PDB ID: 8JT1).

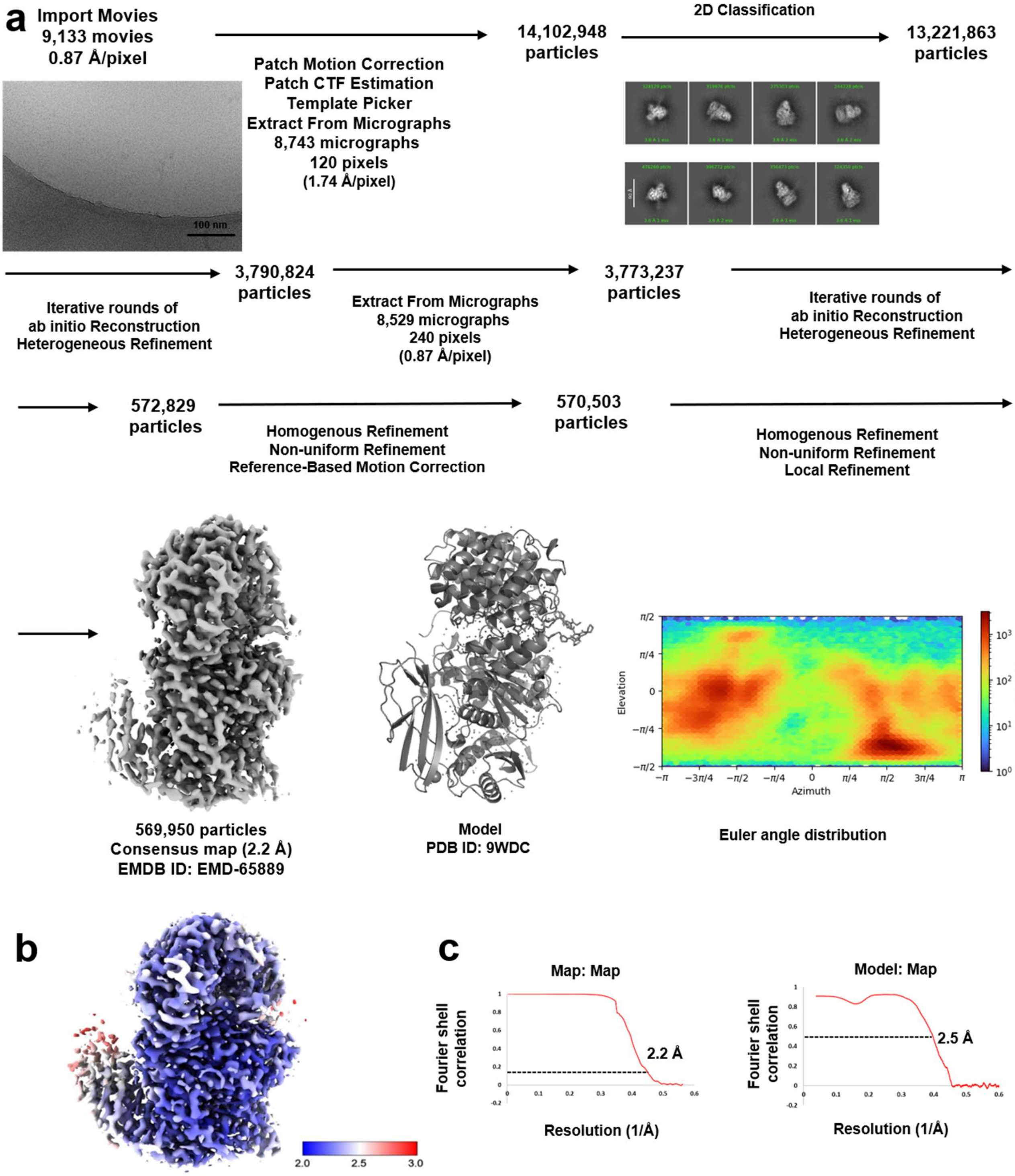

Supplementary Fig. 14. Cryo-EM of 1:1 ColH^{MT}–(PPG)$_{10}$ complex.

a Cryo-EM data processing workflow for 1:1 ColH^{MT}–(PPG)$_{10}$ complex, performed using CryoSPARC[39]. A representative micrograph, 2D class averages, a 3D reconstruction, consensus cryo-EM density map, and Euler angle distribution plot are shown, along with the atomic model of the 1:1 ColH^{MT}–(PPG)$_{10}$ complex. **b** Consensus cryo-EM density map of 1:1 ColH^{MT}–(PPG)$_{10}$ complex, coloured according to local resolution. **c** Gold-standard map-to-map and model-to-map FSC curves. The intersections with FSC = 0.143 (map-to-map) and FSC = 0.5 (model-to-map) are indicated, along with the corresponding resolutions.

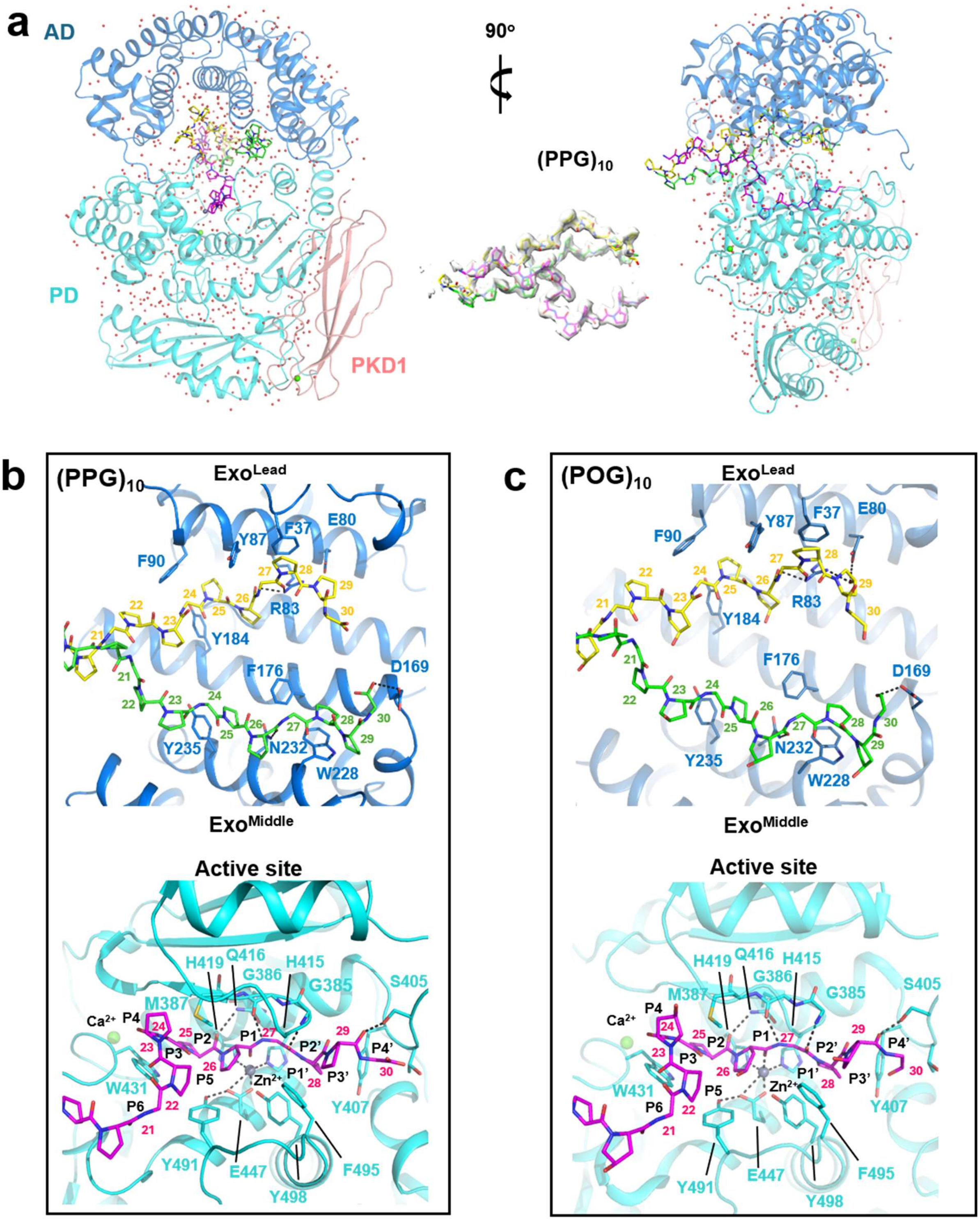

Supplementary Fig. 15. ColH^{MT} binds to a triple-helical collagen model peptide, (PPG)$_{10}$, at the unwound C-terminus. **a** Atomic model of ColH^{MT}–(PPG)$_{10}$ complex with 1:1 stoichiometry. ColH is coloured by domain, using the same colour scheme as in Fig. 1d. The bound (PPG)$_{10}$ is displayed as a stick model, with the leading, middle, and trailing strands coloured yellow, green, and magenta, respectively. The EM density map of (PPG)$_{10}$ regions is also shown. **b** Magnified views of interfaces between leading and middle strands of (PPG)$_{10}$ and the AD (top panel) at Exo^{Lead} and Exo^{Middle}, respectively, as well as between the trailing strand and PD (bottom panel) at the active site. **c** Magnified views of interfaces between leading and middle strands of (POG)$_{10}$ and the AD (top panel) at Exo^{Lead} and Exo^{Middle}, respectively, as well as between the trailing strand and PD (bottom panel) at the active site.

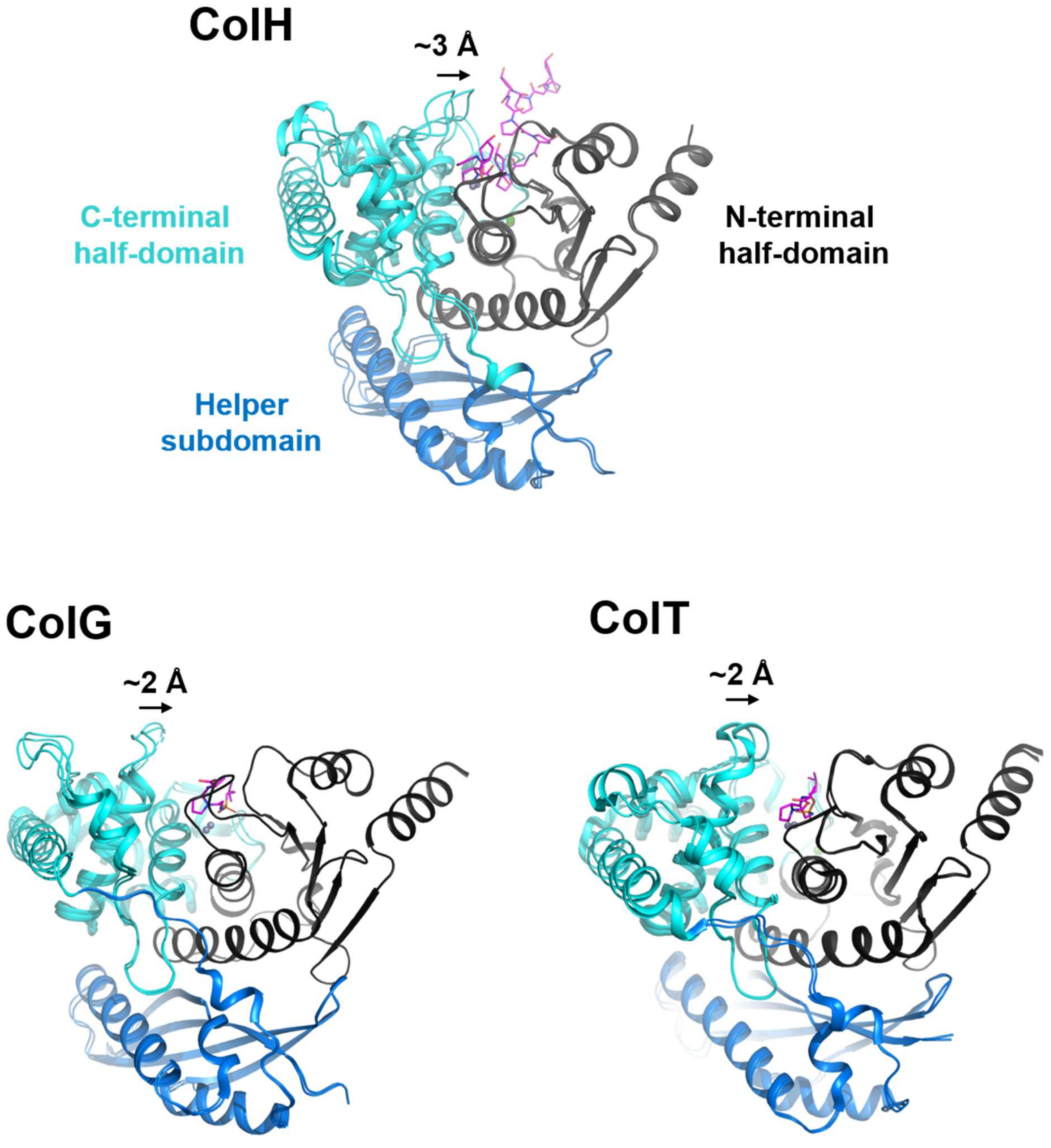

Supplementary Fig. 16. Ligand-induced contraction of PD catalytic subdomain in M9B collagenases.
Superposition of PD structures from $(POG)_{10}$-bound ColH^{MT} (PDB ID: 9LQJ, this study) and substrate-free ColH^{WT} (PDB ID: 9LRK, this study) (top); isoamylphosphonyl-Gly-Pro-Ala-bound (PDB ID: 2Y6I) and inhibitor-free (PDB ID: 2Y50) ColG (bottom left); and isoamylphosphonyl-Gly-Pro-Ala-bound (PDB ID: 4AR8) and inhibitor-free (PDB ID: 4AR9) ColT from *Clostridium tetani* (bottom right). In each comparison, superposition was performed using the N-terminal half-domain. Contraction of the C-terminal half-domain relative to the well-aligned N-terminal half-domain is indicated by arrows. The N-terminal and C-terminal half-domains are coloured grey and cyan, respectively, and the helper subdomain is coloured blue.

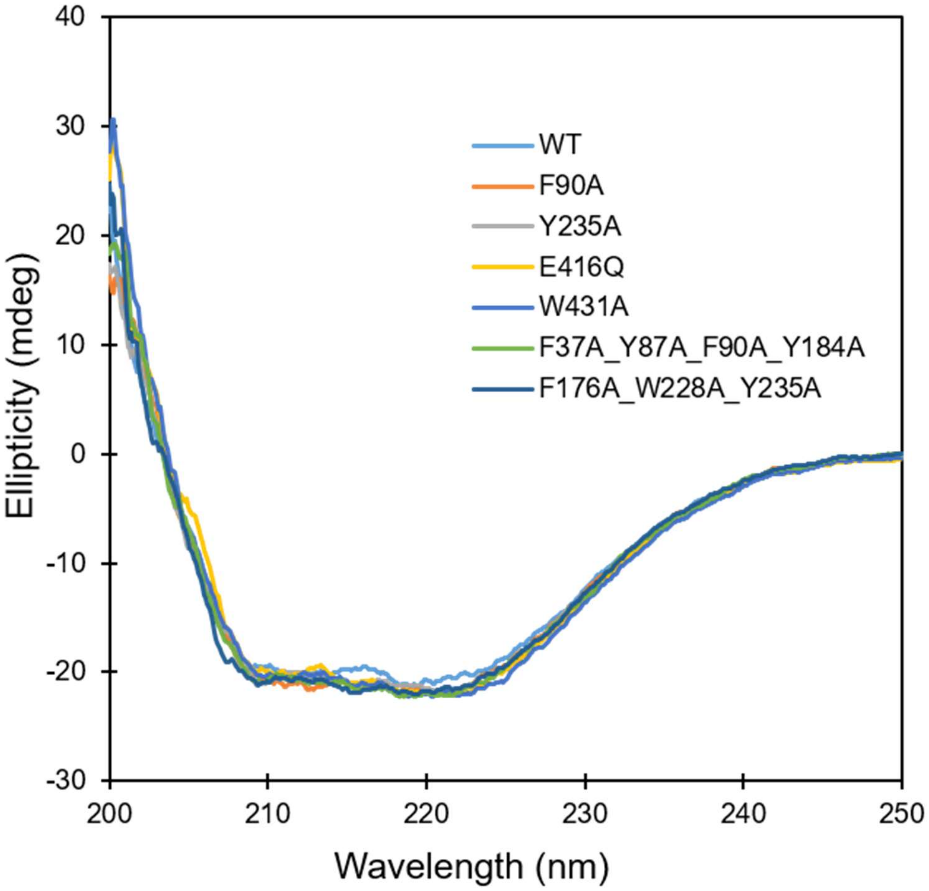

Supplementary Fig. 17. Circular dichroism (CD) spectra of ColH^{WT} and its variants.
Far-UV CD spectra of the wild-type protein and the indicated variants were measured using a Jasco J-720 spectropolarimeter at 200–250 nm, and ellipticity is shown in millidegrees (mdeg). Source data are provided as a Source Data file.

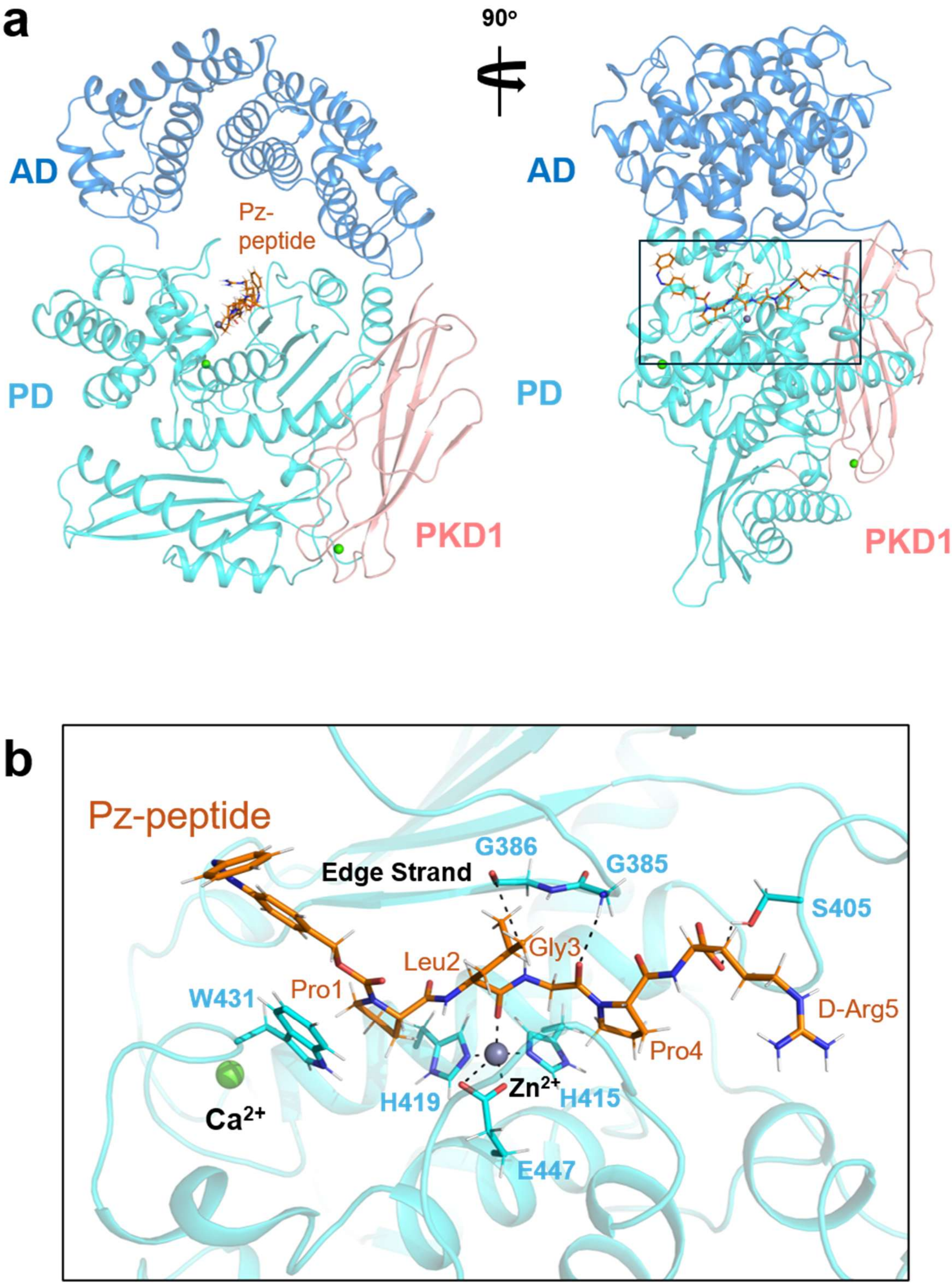

Supplementary Fig. 18. Model structure of ColH^{WT}–Pz-peptide complex.
The structural model of ColH^{WT}–Pz-peptide complex was generated *in silico* by reverting the single mutated residue to wild type and replacing the bound $(POG)_{10}$ peptide with the Pz-peptide in the ColH^{MT}–$(POG)_{10}$ structure (PDB ID: 9LQJ), followed by energy minimisation using the Molecular Operating Environment. **a** Overall structure. **b** Enlarged view of active site in the complex. Pz-peptide bound to PD coordinates the zinc ion via the carbonyl oxygen of Leu2 (P1), and the adjacent Pro1 (P2) forms the CH–π and hydrophobic interactions with Trp431. The orange columns represent the carbon atoms of Pz-peptide.

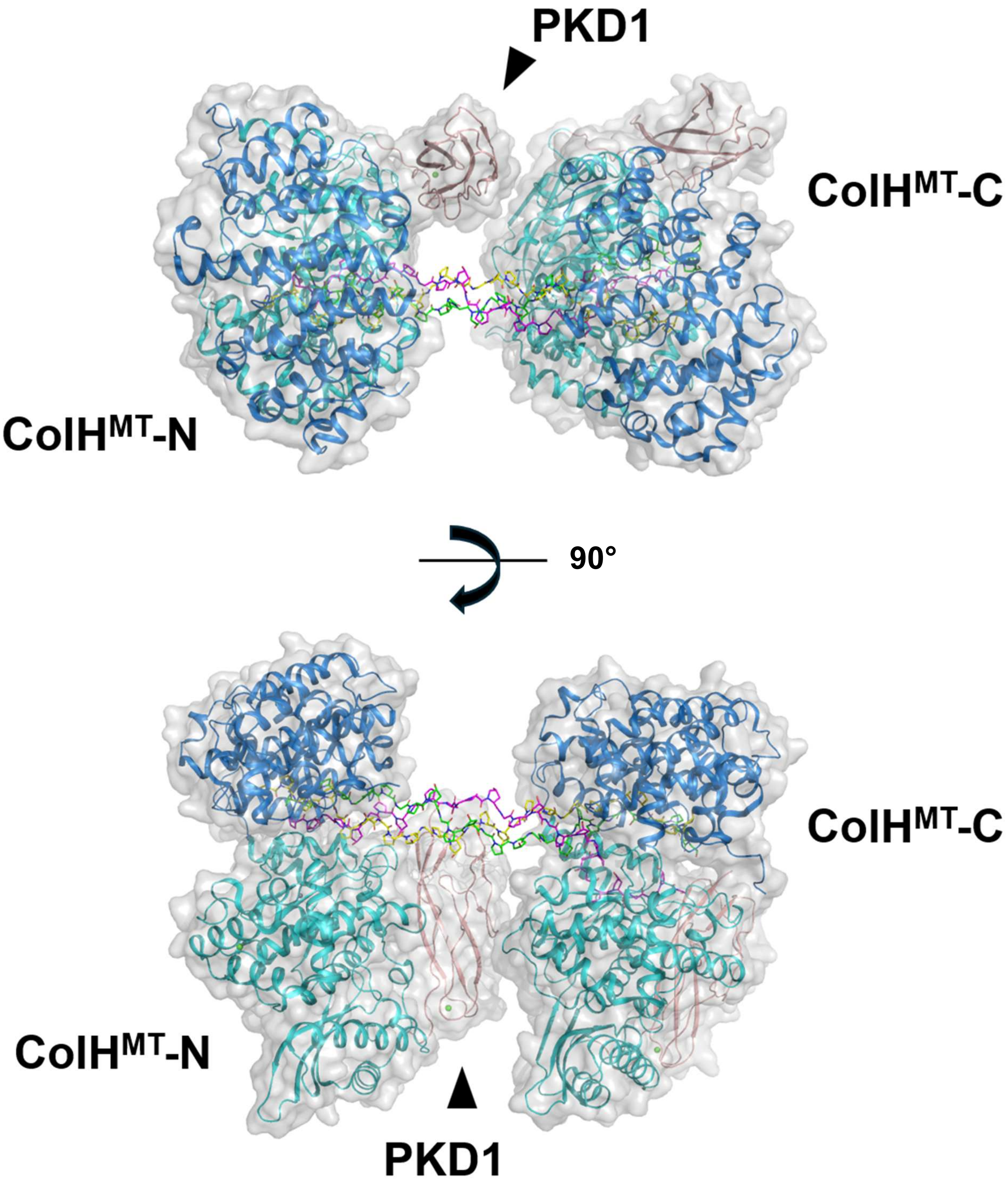

Supplementary Fig. 19. Steric hindrance between PKD1 of ColH^{MT}-N and CM of ColH^{MT}-C in the 2:1 ColH^{MT}–(POG)$_{10}$ complex.
In the complex, ColH^{MT} molecules are depicted as surface representations. The PKD1 of ColH^{MT}-N, which is likely involved in the steric hindrance, is indicated by an arrowhead.

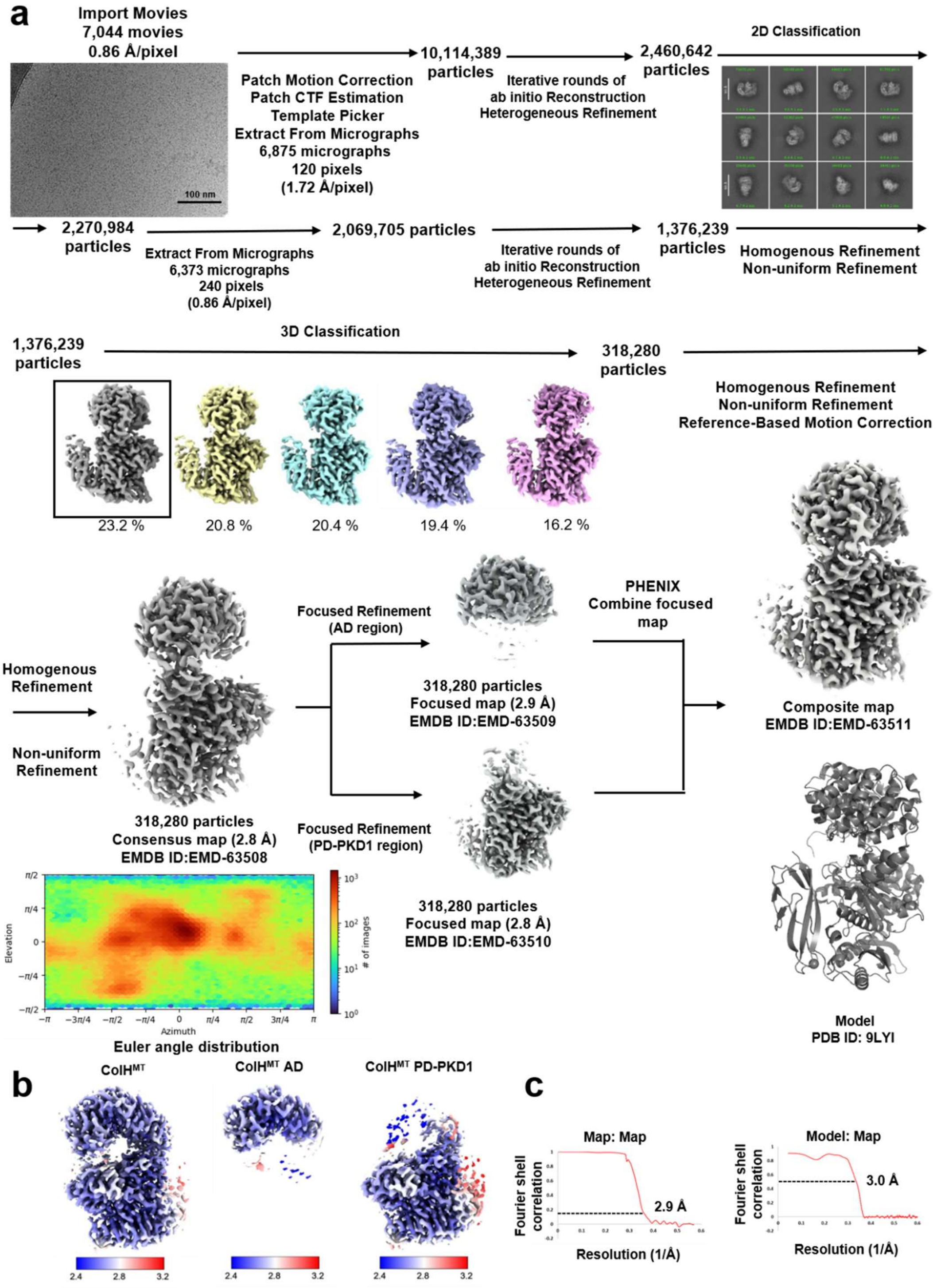

Supplementary Fig. 20. Cryo-EM of ColH^{MT}–(POG)$_{12}$ complex.

a Cryo-EM data processing workflow for ColH^{MT}–(POG)$_{12}$ complex, performed using CryoSPARC[39]. A representative micrograph, 2D class averages, a 3D reconstruction, consensus cryo-EM density map, focused cryo-EM density maps, composite cryo-EM density map, Euler angle distribution plot, and gold-standard model-to-map FSC curve are shown. The intersection with FSC = 0.5 (model-to-map) is indicated, along with the corresponding resolution and atomic model of the ColH^{MT}–(POG)$_{12}$ complex. **b** Cryo-EM density maps of ColH^{MT}–(POG)$_{12}$ complex: Focused cryo-EM density map of the AD region of ColH^{MT} (left panel). Focused cryo-EM density map of the PD–PKD1 region of ColH^{MT} (middle panel). Consensus cryo-EM density map of the ColH^{MT}–(POG)$_{12}$ complex (right panel). The density maps are coloured according to local resolution. **c** Gold-standard model-to-map FSC curve. The intersection with FSC = 0.5 (model-to-map) is indicated, along with the resolution.

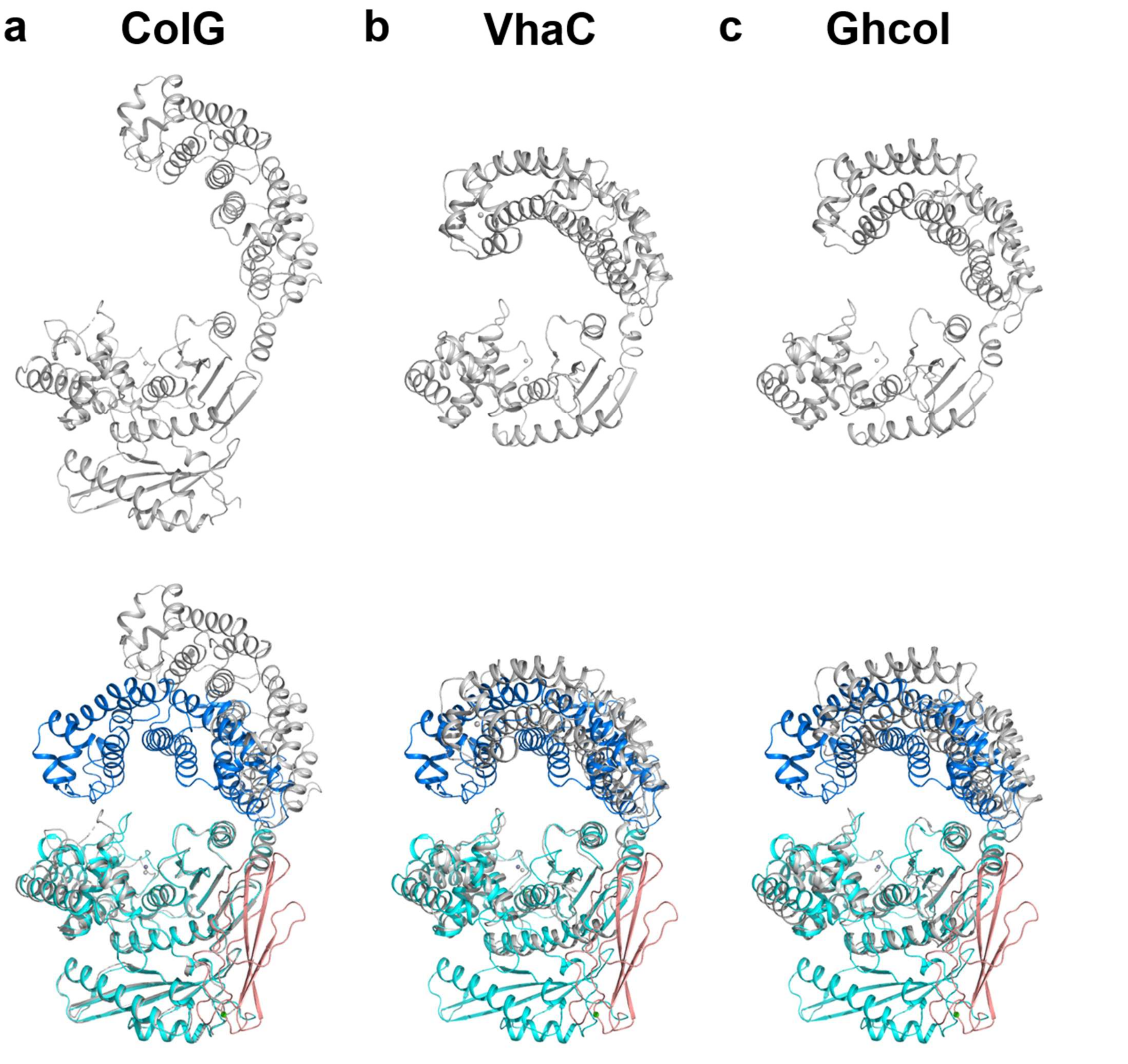

Supplementary Fig. 21. Structural comparison of the CM of M9 family collagenases.
a Overall structure of M9B collagenase ColG from *H. histolytica* (PDB ID: 2Y50). **b** Overall structure of M9A collagenase VhaC from *V. harveyi* (PDB ID: 7ESI). **c** Overall structure of M9A collagenase Ghcol from *G. hollisae* (PDB ID: 7WSS). In each case, the superposition with $ColH^{WT}$ from *H. histolytica* (this study) was performed using the PD catalytic subdomain, shown in the bottom panels.

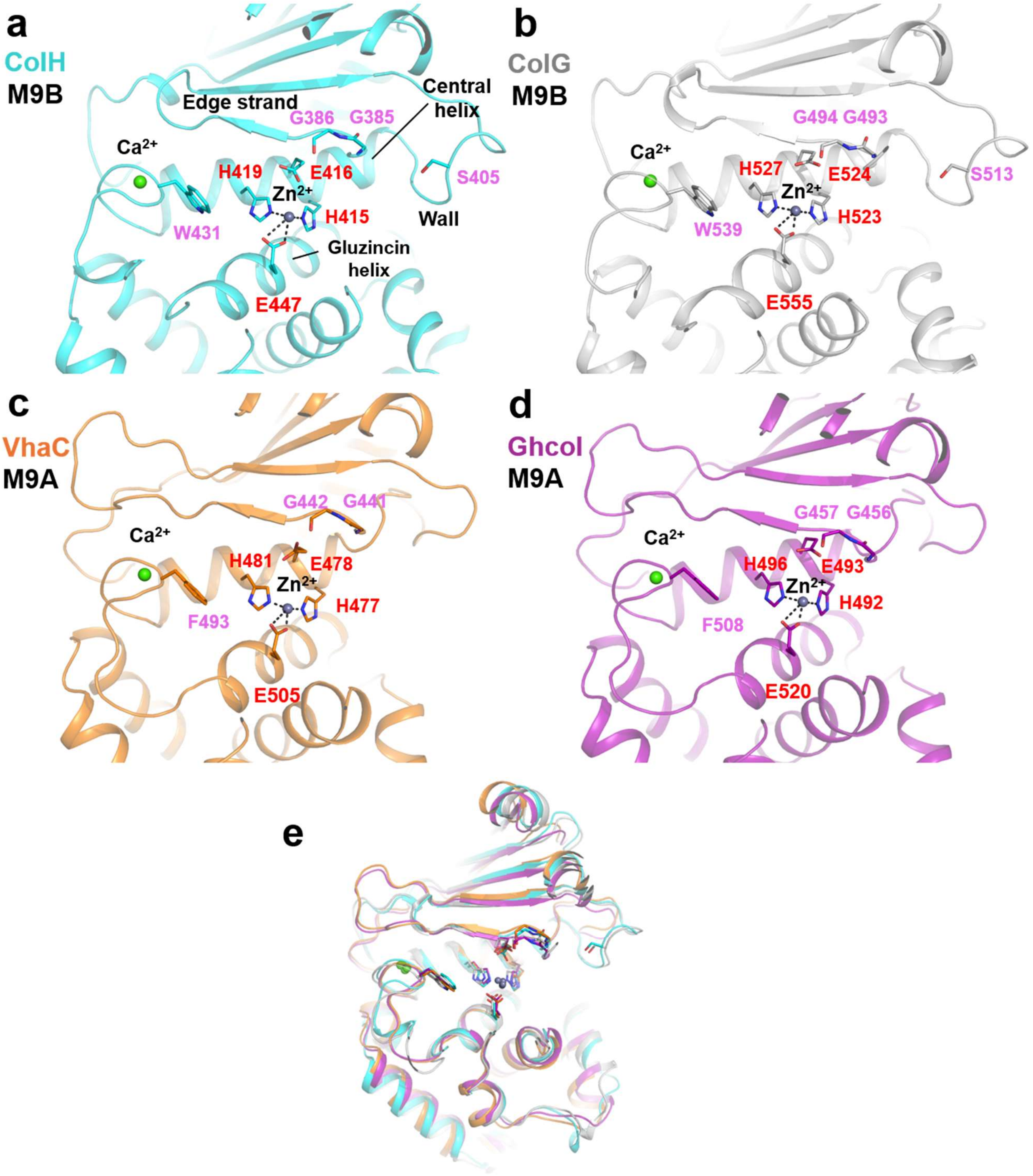

Supplementary Fig. 22. Comparison of active-site cleft structures of the PDs from four bacterial M9 collagenases.
a Active-site cleft structure of ColH from *H. histolytica* (PDB ID: 9LRK, this study). **b** Active-site cleft structure of ColG from *H. histolytica* (PDB ID: 7Z5U). **c** Active-site cleft structure of VhaC from *V. harveyi* (PDB ID: 7ESI). **d** Active-site cleft structure of Ghcol from *G. hollisae* (PDB ID: 7WSS). **e** Superimposition of structures from the four bacterial M9 collagenases listed above.

Supplementary Table 1. Cryo-EM data collection and refinement statistics.

	ColH^{WT} (EMDB-63337) (PDB 9LRK)	ColH^{MT}–(POG)$_{10}$ (1:1) (EMDB-63297) (PDB 9LQJ)	ColH^{MT}–(POG)$_{10}$ (2:1) (EMDB-63339) (PDB 9LRM)	ColH^{MT}–(PPG)$_{10}$ (EMDB-65889) (PDB 9WDC)	ColH^{MT}–(POG)$_{12}$ (EMDB-63511) (PDB 9LYI)
**Data collection and processing**					
Magnification	60,000×	60,000×	60,000×	60,000×	60,000×
Voltage (kV)	300	300	300	300	300
Electron exposure (e–/Å^2)	80	80	80	80	80
Defocus range (μm)	-0.7 to -2.2	-0.7 to -2.2	-0.7 to -2.2	-0.7 to -2.2	-0.7 to -2.2
Pixel size (Å)	0.87	0.87	0.87	0.87	0.87
Symmetry imposed	C1	C1	C1	C1	C1
Initial particle images (no.)	16,098,214	13,017,170	6,141,403	14,102,948	10,114,389
Final particle images (no.)	1,401,659	396,742	58,439	569,950	318,280
Map resolution (Å)	2.3	2.7	3.3	2.2	2.9
FSC threshold	0.143	0.143	0.143	0.143	0.143
**Refinement**					
Initial model used (PDB code)	De novo, ModelAngelo	De novo, ModelAngelo	9LRK, 9LQJ	De novo, ModelAngelo	9LQJ
Model resolution (Å)	2.5	3.0	3.4	2.5	3.0
FSC threshold	0.5	0.5	0.5	0.5	0.5
Map sharpening *B* factor (Å^2)	-100.7	-111.3	-93.5	-81.7	-128.7
Model composition					
Non-hydrogen atoms	6,767	7,068	13,062	6,898	6,404
Protein residues	764	814	1,625	817	793
Ligands	348	431	0	523	74
Ions	1 Zn^{2+}, 2 Ca^{2+}	1 Zn^{2+}, 2 Ca^{2+}	2 Zn^{2+}, 4 Ca^{2+}	1 Zn^{2+}, 2 Ca^{2+}	1 Zn^{2+}, 2 Ca^{2+}
B factors (Å^2)					
Protein	111.35	150.05	111.51	110.26	102.92
Waters	129.08	170.26		123.33	131.77
Ligand	112.21	142.75	116.66	112.80	96.51
R.m.s. deviations					
Bond lengths (Å)	0.004	0.004	0.003	0.003	0.002
Bond angles (°)	0.554	0.570	0.602	0.493	0.442
Validation					
MolProbity score	1.64	1.68	1.70	1.52	1.80
Clashscore	6.13	8.40	13.94	7.53	8.58
Poor rotamers (%)	2.50	2.00	1.00	1.40	2.60
Ramachandran plot					
Favoured (%)	98.95	98.83	97.78	98.89	97.91
Allowed (%)	1.05	1.17	2.02	1.11	2.09
Disallowed (%)	0.00	0.00	0.20	0.00	0.00

Supplementary Table 2. X-ray data collection and refinement statistics.

	$ColH^{WT}$ collagenase module (CM) *
**Data collection**	
Space group	$P2_12_12_1$
Cell dimensions	
a, b, c (Å)	44.1, 126.0, 131.5
α, β, γ (°)	90.0, 90.0, 90.0
Resolution (Å)	22.29 – 2.70 (2.83 – 2.70) **
R_{pim}	0.111(0.232)
CC1/2	0.962 (0.439)
I / σI	6.4 (2.7)
Completeness (%)	99.5 (99.8)
Redundancy	5.7 (6.1)
**Refinement**	
Resolution (Å)	22.29 – 2.70
No. reflections	20,756
R_{work} / R_{free} (5.1%)	0.231 / 0.271
No. of atoms	
Protein	5,531
Ligand/ion	1 Zn^{2+}, 1 Ca^{2+}
Water	243
B-factors	
Protein	44.8
Ligand/ion	40.3
Water	29.5
R.m.s. deviations	
Bond lengths (Å)	0.002
Bond angles (°)	0.490
Ramachandran Plot Statistics (%)	
Favoured regions	95.11
Allowed regions	4.89
Disallowed regions	0

*Diffraction data were obtained from one crystal.

**Values in parentheses are for the highest-resolution shell.

Supplementary Table 3. Mutagenic primers used.

Substitution	Primer sequence (5′–3′)	
Phe37Ala	Forward	GACCTTgcgCAGTATAGTTCAGATGCA
	Reverse	ATACTGcgcAAGGTCTGGTAAATTCTC
Tyr87Ala	Forward	GGATTTgcgTTAGGATTCCATAACAAG
	Reverse	TCCTAAcgcAAATCCAGCTCTTACAAC
Phe90Ala	Forward	TTAGGAgcgCATAACAAGGAATTGAAT
	Reverse	GTTATGcgcTCCTAAGTAAAATCCAGC
Phe176Ala	Forward	GCATTAgcgAATGTTTTAGCTGCACCT
	Reverse	AACATTcgcTAATGCTTTTGACTTTAA
Tyr184Ala	Forward	CCTACCgcgGATATAACTGAGTATTTA
	Reverse	TATATCcgcGGTAGGTGCAGCTAAAAC
Trp228Ala	Forward	AACTCTgcGATAATAGATAACGGTAT
	Reverse	TATTATCgcAGAGTTATTATCATTTAT
Tyr235Ala	Forward	ATAGATgcgGGTATATATCATATAGCA
	Reverse	TATACCcgcATCTATTATCCAAGAGTT
Glu416Gln	Forward	TAGACATcAATATACACATTATTTGC
	Reverse	GTATATTgATGTCTAAATAATTCTTC
Trp431Ala	Forward	GGACAAgcgGGAAGAACAAAACTTTA
	Reverse	TCTTCCcgcTTGTCCTGGAACTGCATA

Lowercase letters indicate mutated nucleotides.
