# Supplementary material for: Bacterial collagenase harnesses collagen geometry for processive cleavage
Source: Nat Commun. 2026 Apr 2;17:5485. doi: 10.1038/s41467-026-71099-3 (PMC13284217; doi:10.1038/s41467-026-71099-3)
Supplement: Supplementary file 2 — Description of Additional Supplementary Files [file 41467_2026_71099_MOESM2_ESM.pdf]

Description of Additional Supplementary Files

Supplementary Movie 1. 3D variability analysis (3DVA) of ColH^{WT} along the first principal component (close–open motion)

The video shows a closing-opening conformational motion of the ColH^{WT} collagenase module, as revealed by 3DVA (corresponding to Supplementary Fig. 5).

Supplementary Movie 2. 3D variability analysis (3DVA) of ColH^{WT} along the second principal component (twist motion)

The video shows a twisting conformational motion of the ColH^{WT} collagenase module, as revealed by 3DVA (corresponding to Supplementary Fig. 5).

Supplementary Movie 3. 3D variability analysis (3DVA) of the 2:1 ColH^{MT}–$(POG)_{10}$ complex

The video shows the structural heterogeneity of the 2:1 ColH^{MT}–$(POG)_{10}$ complex, as revealed by 3DVA, highlighting conformational heterogeneity of ColH^{MT}-N at the N-terminal binding site of $(POG)_{10}$ (corresponding to Supplementary Fig. 10).
