# Supplementary material for: Bacterial collagenase harnesses collagen geometry for processive cleavage
Source: Nat Commun. 2026 Apr 2;17:5485. doi: 10.1038/s41467-026-71099-3 (PMC13284217; doi:10.1038/s41467-026-71099-3)
Supplement: Supplementary file 6 — Reporting Summary [file 41467_2026_71099_MOESM6_ESM.pdf]

nature portfolio

Corresponding author(s): Osamu Matsushita, Joshua Sakon, Kazuki Kawahara

Last updated by author(s): Mar 7, 2026

Reporting Summary

Nature Portfolio wishes to improve the reproducibility of the work that we publish. This form provides structure for consistency and transparency in reporting. For further information on Nature Portfolio policies, see our Editorial Policies and the Editorial Policy Checklist.

Statistics

For all statistical analyses, confirm that the following items are present in the figure legend, table legend, main text, or Methods section.

n/a	Confirmed	
☐	☒	The exact sample size (*n*) for each experimental group/condition, given as a discrete number and unit of measurement
☐	☒	A statement on whether measurements were taken from distinct samples or whether the same sample was measured repeatedly
☐	☒	The statistical test(s) used AND whether they are one- or two-sided<br>*Only common tests should be described solely by name; describe more complex techniques in the Methods section.*
☒	☐	A description of all covariates tested
☒	☐	A description of any assumptions or corrections, such as tests of normality and adjustment for multiple comparisons
☐	☒	A full description of the statistical parameters including central tendency (e.g. means) or other basic estimates (e.g. regression coefficient) AND variation (e.g. standard deviation) or associated estimates of uncertainty (e.g. confidence intervals)
☐	☒	For null hypothesis testing, the test statistic (e.g. *F*, *t*, *r*) with confidence intervals, effect sizes, degrees of freedom and *P* value noted<br>*Give P values as exact values whenever suitable.*
☒	☐	For Bayesian analysis, information on the choice of priors and Markov chain Monte Carlo settings
☒	☐	For hierarchical and complex designs, identification of the appropriate level for tests and full reporting of outcomes
☒	☐	Estimates of effect sizes (e.g. Cohen's *d*, Pearson's *r*), indicating how they were calculated

Our web collection on statistics for biologists contains articles on many of the points above.

Software and code

Policy information about availability of computer code

Data collection	SerialEM (version 4.1.6) was used for automated cryo-EM data collection.<br>No custom or proprietary software was employed for data acquisition.
Data analysis	CryoSPARC (v4.4.1-4.6.0) was used for motion correction, particle picking, 2D/3D classification, refinement, and 3D variability analysis. PHENIX (v1.19.2-4158 or v1.20.1-4487), Coot (v0.8.9.2 or 0.9.8.93 EL), and CCP4 suite (v8.0.019, including REFMAC5) were used for model building and refinement of cryo-EM and crystallographic structures. Amino-acid sequences were aligned using Clustal Omega (v1.2.4). Secondary-structure elements of protein structures were assigned using DSSP (v4). Structural modelling was performed using MOE software (v2020). UCSF ChimeraX (v1.9-1.10.1), PyMOL 2.6, and PyMOL 3.0 were used for structural visualization and figure generation. Spectra Manager (version 1.55.00, JASCO) was used for acquiring and processing circular dichroism (CD) spectra.No custom code was used.

For manuscripts utilizing custom algorithms or software that are central to the research but not yet described in published literature, software must be made available to editors and reviewers. We strongly encourage code deposition in a community repository (e.g. GitHub). See the Nature Portfolio guidelines for submitting code & software for further information.

Data

Policy information about availability of data

All manuscripts must include a data availability statement. This statement should provide the following information, where applicable:

- Accession codes, unique identifiers, or web links for publicly available datasets
- A description of any restrictions on data availability
- For clinical datasets or third party data, please ensure that the statement adheres to our policy

All cryo-EM maps and atomic coordinates are publicly available under the following accession codes:EMDB: EMD-63331, EMD-63333, EMD-63332, EMD-63337, EMD-63297, EMD-63334, EMD-63336, EMD-63335, EMD-63339, EMD-65889, EMD-63509, EMD-63510, EMD-63508, EMD-63511.PDB: 9CME, 9LRK, 9LQJ, 9LRM, 9WDC, 9LYI.All data supporting the findings of this study are available within the paper and its supplementary information files.There are no restrictions on data availability.

Research involving human participants, their data, or biological material

Policy information about studies with human participants or human data. See also policy information about sex, gender (identity/presentation), and sexual orientation and race, ethnicity and racism.

Reporting on sex and gender	Not applicable. This study did not involve human participants, clinical data, or biological samples.
Reporting on race, ethnicity, or other socially relevant groupings	Not applicable. No human participants or human-derived materials were used.
Population characteristics	Not applicable.
Recruitment	Not applicable.
Ethics oversight	Not applicable. Ethical approval was not required as the study used recombinant bacterial proteins and synthetic peptides only.

Note that full information on the approval of the study protocol must also be provided in the manuscript.

Field-specific reporting

Please select the one below that is the best fit for your research. If you are not sure, read the appropriate sections before making your selection.

☒ Life sciences ☐ Behavioural & social sciences ☐ Ecological, evolutionary & environmental sciences

For a reference copy of the document with all sections, see nature.com/documents/nr-reporting-summary-flat.pdf

Life sciences study design

All studies must disclose on these points even when the disclosure is negative.

Sample size	Sample sizes were determined based on standard practices in biochemical and structural biology experiments. For enzymatic assays, each measurement was performed in biological triplicates with three technical replicates (n = 9), which was sufficient to ensure statistical significance. The digestion assay summarized in Supplementary Figure 11 is not intended to provide quantitative or statistical analysis, but rather to demonstrate a qualitative observation supporting the experimental rationale. No statistical inference or comparative analysis is derived from this dataset. Thus, the experiment was performed once (n = 1), and the result is presented as a representative qualitative outcome. For cryo-EM and crystallography, data quantity was determined by map resolution and completeness, not by pre-specified sample size. Circular dichroism (CD) spectra were collected once for each protein variant. Each spectrum represents the average of eight accumulations.
Data exclusions	For protein assay and Pz-peptidase assay, no data were excluded. Since insoluble collagen powder is used as the substrate for collagenase assay, outliers can occur due to the substrate heterogeneity. Hence, each assay run was carried out in quadruplicate, and one outlier was excluded from each data set. For all the structural studies, no data were excluded. All collected datasets that met quality control criteria were included in the analyses.
Replication	Enzymatic assays were repeated independently three times with consistent results. Cryo-EM and crystallographic data were each collected once for the final structures; reproducibility was confirmed by internal validation metrics (map resolution, model geometry, and cross-validation statistics). CD measurements were performed once per sample without biological or technical replicates.
Randomization	Not applicable. No randomization was required because this study did not involve living organisms or treatment groups.<br>All biochemical experiments were performed under defined, reproducible conditions.
Blinding	Not applicable. Investigators were not blinded during data collection or analysis, as blinding is not relevant for biochemical and structural experiments.

Reporting for specific materials, systems and methods

We require information from authors about some types of materials, experimental systems and methods used in many studies. Here, indicate whether each material, system or method listed is relevant to your study. If you are not sure if a list item applies to your research, read the appropriate section before selecting a response.

Materials & experimental systems

n/a	Involved in the study
☒	☐ Antibodies
☒	☐ Eukaryotic cell lines
☒	☐ Palaeontology and archaeology
☒	☐ Animals and other organisms
☒	☐ Clinical data
☒	☐ Dual use research of concern
☒	☐ Plants

Methods

n/a	Involved in the study
☒	☐ ChIP-seq
☒	☐ Flow cytometry
☒	☐ MRI-based neuroimaging

Plants

Seed stocks	Not applicable. No plant materials were used in this study.
Novel plant genotypes	Not applicable. This study did not involve any plant genetic modification or transgenic lines.
Authentication	Not applicable. No plant samples or genotypes were used or generated
