# Supplementary material for: Bacterial collagenase harnesses collagen geometry for processive cleavage
Source: Nat Commun. 2026 Apr 2;17:5485. doi: 10.1038/s41467-026-71099-3 (PMC13284217; doi:10.1038/s41467-026-71099-3)
Supplement: Supplementary file 7 — Transparent Peer Review file [file 41467_2026_71099_MOESM7_ESM.pdf]

nature portfolio

Peer Review File

Bacterial Collagenase Harnesses Collagen Geometry for Processive Cleavage

Corresponding Author: Dr Kazuki Kawahara

This file contains all reviewer reports in order by version, followed by all author rebuttals in order by version.

Version 0:

Reviewer comments:

Reviewer #1

(Remarks to the Author)
In this manuscript Oki H et al. describe cryo-EM and X-ray structures of the H. histolytica collagenase ColH, a processive carboxytripeptidase. The structures presented reveal distinct states of collagen triple helix engagement and unwinding, providing unprecedented insight into the mechanism of collagen engagement and cleavage. Overall, this is a very well performed study that sheds new light on an interesting bacterial collagenase with potential for future engineering or inhibitor development. For the most part the manuscript is very clearly written, and should be easily understood even by a non-expert in the field. There are extensive supplemental and extended data figures that are well prepared to deliver the overall story. Overall, I am highly supportive of publication of this manuscript, as I feel the studies have been rigorously performed with appropriate analysis. I have only minor comments for the authors to consider before final publication...

1. Line 98 - "Although low in amplitude, these motions generate an entrance of ~15Å, sufficient to capture a tropocollagen molecule in the central cavity". It may be nice to show a (supplemental/extended) figure with a tropocollagen molecule at scale next to the opening. It is hard to tell from the 3DVA alone just how large the opening would need to be in order for the "clamp" to open sufficiently to accommodate entry of tropocollagen.

2. While the resolutions reported in most of the cryo-EM experiments are indeed possibly suitable for placement of water molecules, I am skeptical of the placement and total number of water molecules included in the refined structures. In particular, in Figure 1B and 1D it is clear that the resolution in the Activator Domain is significantly lower due to inherent flexibility, yet water molecules are still modelled around the periphery of the activator domain. I am skeptical that these are truly water molecules, rather than simply "dust" generated by the automatic masking process in cryosparc. While well ordered water molecules in the protein interior with good stable hydrogen bonding are likely observed in the best resolved regions, the waters that are placed at the periphery of the protein (and especially in the Activator Domain) are skeptical at best. It may be worthwhile for the authors to carefully review the placement of water molecules.

3. In addition to the point above, can the authors clearly see water molecules near the zinc active site? Including a supplemental figure demonstrating well resolved water molecules around the zinc active site would be ideal. From the placement of these water molecules is it possible to delineate the precise positioning of the water that will attack the peptide carbbonyl? This information could be very useful to those studying zinc metalloproteases more generally.

3. Something seems weird in the methods section about box and pixel sizes. Line 386 - "with a box size of 256 pixels (1.86 Å/pix) ... particles were re-extracted with a box size of 256 pixels at 0.87 Å/pix". I think what the authors mean to say is that particles were originally extracted at a box size of ~550 pixels and downsampled to a box size of 256 pixels (1.87Å) before initial rounds of 2D classification? I don't see how the particles could be extracted with the same box size and different pixel sizes unless the micrographs themselves were binned at some point, or during extraction the particles were also downsampled. Please clarify in all sections throughout the methods.

4. Line 118 - "unwinding its terminal three triplets in an N-to-C direction". This statement confused me when I first read it. Couldn't one argue that the actual unwinding occurs from a C-to-N direction? Figure 5 makes this a little more clear, but at this point in the manuscript I was slightly confused by this wording and the diagram shown in Figure 2. Please see if revision makes sense, or this statement is accurate. As a reader I may be getting confused between "unwinding" and "translocation".

5. Line 149 - it may be good to include a reference for the role of Calcium coordination in catalysis.

Reviewer #2

(Remarks to the Author)
The collagenolytic mechanism of M9 collagenases has been extensively studied. The collagen triple helix is the minimal structural unit of collagen, and it is generally believed that this triple helical molecule must be locally unwound to release a single peptide chain into the catalytic cavity before hydrolysis (2012 NSMB; 2022 NC; 2024 PNAS). However, direct evidence showing that any M9 collagenase actively drives this unwinding process has been lacking. In this context, the work by Oki et al. provides valuable structural and biochemical insights. The authors cleverly utilized a collagen synthetic peptide that forms a stable triple helix in solution and resolved cryo-EM structures of M9B collagenase ColH in complex with POG10 in both its triple helical and unwound forms. Combined with biochemical assays, this work explores the unwinding mechanism of the collagen triple helix by ColH. Despite these important contributions, there are several concerns that need to be addressed before publication.

1. From a three-dimensional structural perspective, the W431 residue is far from the catalytic center of ColH. However, as shown in Fig. 2g, the W431 mutation to alanine not only significantly reduced the activity of collagen but also almost completely lost the activity of Pz peptide. There is no doubt that this phenomenon is counterintuitive. Although the author provided relevant explanations in L148-L149, the significance of the W431 residue of ColH in triple helix unwinding or catalysis deserves to be reconsidered. The solution to this question would benefit from circular dichroism spectroscopy analysis of mutant W431A and even other mutants to determine that the decreased collagen-binding ability of the mutants is caused by amino acid replacement rather than structural alteration. In addition, given the significance of the W431 residue in the triple helix unwinding process, the authors are encouraged to resolve the complex structure of W431A and POG10.

2. L146: What kind of interaction exists between W431 and P25 and P22? It is recommended a close-up view of the relative positions or interactions between relevant amino acids in Fig. 2e.

3. In the complex structure of ColH and POG12, why is the triple helix form of POG12 not visible? Can the author make a reasonable speculation and elaborate on it in the text? Is it related to its own properties of POG12 or did the enzyme molecule ColH mediate the complete unwinding of the triple helix molecule?

4. The conservation of key sites (W431, F90, Y235, etc.) related to triple helix unwinding throughout the M9 family collagenases (M9A and M9B) needs to be explored.

5. The proposed mechanism is mainly inferred from structural data, lacking direct kinetic or biochemical evidence. The discussion should explicitly acknowledge this limitation.

6. The feasibility of ColH fully wrapping around a single collagen chain within densely packed fibrils is questionable. The authors should further discuss how ColH might act on native collagen substrates, going beyond the simple statement that ColH targets only partially unwound or damaged regions of collagen fibrils. In addition, the potential auxiliary roles of PKD and CBD domains in substrate recognition, binding, and unwinding should be considered to strengthen the physiological relevance of the proposed mechanism.

Reviewer #3

(Remarks to the Author)
The manuscript by Oki and colleagues entitled "Bacterial Collagenase Harnesses Collagen Geometry for Processive Cleavage" reports on a study that investigates the mechanism of how bacterial collagenases unwind and cleave collagen. Collagen's triple-helical fold and high content of (hydroxy-)proline render the molecule extremely stable and resistant to enzymatic proteolysis within the context of the extracellular matrix. To overcome this difficulty, true collagenases, i.e. those enzymes that process natively folded triple-helical collagen, must unwind the molecule locally to make the cleavage sites accessible to the enzyme. Based on high-resolution cryo–electron microscopy findings the authors demonstrate that ColH encircles a single collagen triple helix via its AD and PD domains. Via the hydrophobic AD exosites the triple helix is eventually stripped of its stabilizing hydration shell by the enzymes, and the substrate's leading and middle strand are bound and sterically distorted by binding to the two AD exosites. The displaced trailing strand is threaded via "bight" formation mediated by Trp431 into the active-site cleft. Exploiting collagen's geometry for continuous degradation, the authors propose that small-amplitude AD-PD movements, i.e. cycling between "ratcheted" and "closed" stated, allow for triplet-by-triplet translocation and its stepwise processing.
The present work is of great importance to the field and beyond, and deserves publication in Nature Communications. Merely, the following minor issues should be dealt with in a revised version of the manuscript before prior to acceptance for publication.

The numbering of the references is not chronological. Please correct.

It would ease visual comparison with past publications in the field, if the authors would use the conventional presentation of the structure of the bacterial collagenases starting with AD on the left followed by the PD on the right etc., i.e. a horizontal

presentation of the structure instead of the chosen “vertical one”.
The horizontal presentation of the collagenase has the added benefit that it is conforms better with the standard orientation for (metallo-)peptidases (DOI: 10.1016/j.bbapap.2011.04.014).

Just a suggestion, but the established notation for indicating the sequence of collagen-like peptides is easier on the eye, i.e. (POG)10 not POG10.

Line 45: Please insert for better clarity “triple-helical” to yield “The triple-helical conformation is …”

Line 51: Please insert “which” as follows in “infections and [which] have been adapted”

Line 54: Please remove “and non-fibrillar”. Eckhard et al (2011) & Fields (2013) do not claim that bacterial collagenases can digest non-fibrillar collagen; the authors only refer to fibrillar collagens I, II and III.

Line 54: While Eckhard et al (2011) and Wang et al (2022) have provided first evidence regarding the essential role of the AD for collagen recognition and processing, Serwanja et al. (2024) demonstrated that the AD is responsible for collagen unwinding. The authors also identified residues required for collagen unwinding. Therefore, the phrase “PD-mediated cleavage may depend on prior AD-mediated unwinding” seems misleading regarding the processing of folded collagen. Please adapt.

Line 62: The authors should give credit to previous studies that established that bacterial collagenases and MMPs employ fundamentally different unwinding mechanisms.

Line 69: Alhayet et al (2022) did not provide structural information on the conformation of M9 collagenases. Therefore, please remove.

Line 71: Please replace “unlay” with “unwind” or “untwist”.

Line 80: The collagenase module (CM) is composed of the activator domain (AD) and the peptidase domain (PD) (Eckhard et al (2011)). According to Eckhard et al (2011), the part that in this manuscript is referred to as “helper domain” constitutes a PD subdomain. If the authors can provide evidence that the helper subdomain of ColH is able to fold independently of the residual PD, then it reasonable to label it as an independent domain. But otherwise please adhere to the established nomenclature and denote it as “helper subdomain”. Please correct the terminology in the manuscript/supplement/figures (either by differentiating within the PD between the “catalytic subdomain” and the “helper subdomain”, where appropriate in the text/figure; or by just referring to the “PD” (not PD-HD).

Line 85: The authors’ phrasing “The composite EM map of the AD and other domains” is suboptimal, as the term “other domains” at this point implies PD, PKD1, PKD2 and CBD. Therefore, change to “The composite EM map of the CM” instead. In particular, this misunderstanding can arise because the authors did not reveal any information regarding the map of the domains C-terminal of the CM up to this point. A suggestion would be to move the contents of this paragraph (line 100-105) after line 84, before continuing with the text in line 85.

Can the authors comment/speculate why the CBD domain was not visible in the EM map?

Line 85: For better local clarity please modify to “At the tip of the CM, three helices (α17 from the AD, and α18 and α21 from the PD) formed …”

Fig. 1d, Inserts: Please color the helix labels differently to make them easier to find.

Fig. 1f: The shift between the frames 0 and 9 is very difficult to discriminate, in particular on the left side of the panel.

Line 89 - line 92:
The authors’ conclusion regarding AD–PD interdomain flexibility is difficult to evaluate without comparing the RMSD values from the individual domain alignments to the RMSD from the overall (CM) alignment. Please clarify this point for better transparency and understandability.

Line 95: Please correct to “To assess CM flexibility,”

Line 99: How do these low-amplitude motions compare to the “open” conformations reported by Eckhard et al./Wang et al. & Ikeuchi et al.? Please elaborate.

Line 128-139:
While the authors emphasize the contribution of hydrophobic interactions to substrate recognition, the role of hydrogen-bonding interactions—also clearly present—is not discussed. The hydrophobic contacts may be of greater significance. But their significance regarding the function of the AD is not corroborated by mutational studies. The proposed binding mode remains incomplete without consideration of these additional hydrogen bonds.
Specifically, hydrogen-bonding interactions stabilize the main chain of the leading strand: Arg83:NH2 forms hydrogen bonds with Gly27 and Hyp26 (2.7 Å and 2.9 Å, respectively). In addition, the position of OD1 of Hyp29 is stabilized through hydrogen bonds with Glu80 (2.8 Å) and Arg83 (3.0 Å). Both Glu80 and Arg83 appear in Figure 2e, but are not mentioned in

the text. These residues are ideally positioned to stabilize the Hyp residue at the Y-position of the collagen-like sequence, whereas in ExoMiddle, the corresponding OD1 stabilization is mediated by the main-chain oxygen of Asp224.
In ExoMiddle, the CH–π interaction involving His236—observed in the $(PPG)_{10}$ and $(POG)_{10}$ model — is not described. Although the hydrogen-bonding network in this second exosite is less extensive than in ExoLead, interactions by Asp231, Arg232, and Asp224 are still present. Similarly to Glu80 and Arg83 in ExoLead, Asp231 can stabilize the hydroxyl group of Hyp26 in the Y-position. This stabilization is absent in 9LQJ, but evident in 9LYI.
Finally, it would strengthen the manuscript to mention the findings of Serwanja et al., which identified the corresponding residues to Phe37, Glu80, Arg83, Tyr87, Phe90, and Tyr184 in ExoLead in ColH as key substrate-binding residues in ColG.

Line 135: "Most Hyp residues remained solvent-exposed": While this is correct, it is, nonetheless, interesting that in ExoLead and ExoMIddle specific pockets have evolved to stabilize the hydroxyl group of Hyp residues in the Y-position. The involved residues seem to be highly conserved in the M9 family.

Line 142: "the active-site cleft, which contracted to hold the strand tightly". Please substantiate this observation (with a figure or quantify the extent of the contraction), which is – as a remark - in line with observations by Eckhard et al. (2011) NSMB and Eckhard et al. (2013) JBC.

Line 144: "with conserved Gly385 and Gyl386, known as the double-glycine S1' recognition site (Ref. 3, 17)"

Fig. 1: In Fig. 2-4, the authors provide an image illustrating the quality of the EM map by showing the EM map surrounding the collagen-like peptides. Also in Fig. 1, it would be of benefit to illustrate the quality of the high-resolution EM map. In case the visualization allows it, the residue sticks in the inserts, for example, could be overlaid with the EM map.

Fig. 2 g: The labeling of "Pz-peptidase" and "collagenase" could potentially confuse the reader. Different variants of ColH were tested against two different substrates, i.e. Pz-peptide and collagen. Therefore, I recommend using the substrate names in the legend on top of the bar graph. Please amend the figure caption stating that E416Q represents a negative control.

Line 147-148: The collagenase and peptidase activity of ColH were reduced by the Trp431Ala mutation. The reduction in turnover of both substrates is understandable given the important role of Trp431 in correctly positioning/orienting the substrate "bight" to enable binding of the residual C-terminal residues into the active-site cleft.

Line 148: "Because Trp431 lies on a calcium-binding loop, these results further support the essential role of Ca2+ coordination in ColH catalysis." This statement might not be appreciated by the audience, when they are not familiar with the significance of this calcium-binding site in bacterial collagenases. In Eckhard et al. (2013) (Ref. 17) it was proposed that "[d]ue to its location between the two catalytic helices, calcium binding braces the upper and lower half-domains of the peptidase and stabilizes thereby the zinc coordination" in the active site. Thus, calcium binding, on the one hand, stabilizes the zinc in the active site, and, at the same time, the calcium-binding loop also ensures the correct positioning of Trp431 for pre-orienting the substrate and "threading" it into the active-site cleft.

Line 150-153:
Quantitative comparisons would help to clarify the magnitude of the observed effects in the alanine scanning. From the results shown in Fig. 2g, it can be concluded that Trp431 plays a vital role in substrate turnover for unfolded and triple-helical substrates. In case of Tyr235Ala, the mutation had a noticeable effect (~30% decrease in collagenolytic activity), whereas when all three aromatic residues of ExoMiddle were mutated an overall decrease by ~65% was observed. In comparison, the Phe90Ala mutation in ExoLead alone reduced the activity by approx. 60%. Alanine mutation of the three residual aromatic residues of this exosite led to a further decrease by 30%. This seems to suggest that the presented "three pivots" differ in their functional significance. Please elaborate more on this point. While the significance of Trp431 and Phe90 become apparent from the presented data, it is difficult to ascertain whether Tyr235 is the most major determinant in ExoMiddle, when the single alanine mutations of the other residues of this exosite are not presented. I would recommend to add more mutational data regarding ExoMiddle.

Line 154: I would recommend to rephrase the second part of the sentence for better clarity [regarding "or favours partially unfolded ….. POG10"].

Line 164: "they may [have] facilitate[d] additional ColH recruitment"

Line 178:
The authors state that "ExoLead-driven unwinding of POG10 was restricted because ColHMT-C bound at the C-terminus blocks further unravelling by ColHMT-N." The observation that ColHMT-N was unable to unwind the bound triple-helical collagen is interesting. The authors conclude that ColHMT-N could not unwind the triple helix due to the presence of ColHMT-C, implying that the C-terminal unwinding event precludes N-terminal unwinding. Can the authors clarify whether this interpretation is consistent with the conclusions drawn for the complex with (POG)12? At first glance, the two conclusions/statements appear to be contradictory.
Staying with the statement "ColHMT-C bound at the C-terminus blocks further unravelling by ColHMT-N": The authors seem to suggest that C-terminal unwinding induced a strain in the triple-helix which precluded a second unwinding event in close proximity. Or do I misinterpret the author's statement? If yes, please provide an alternative explanation for the blockage? If no, is there any indication in the structural data to support the "strain hypothesis"?
There could be an alternative interpretation of the observed absence of unwinding in case of ColHMT-N, namely that due to

the absence of the middle and trailing strand at the recognition site of the leading strand (due to the mis-stagger) the local structural perturbation induced by ExoLead on the leading strand could not be further sterically propagated to the middle and trailing strand (which would then have been facilitated and stabilized by binding to ExoMiddle and the active-site cleft), thus preventing local unwinding and thus resulting in an unproductive binding event. Would this interpretation be consistent with the findings regarding the complex with (POG)12?

Lines 200-201: Concerning the observed complete unfolding of $(POG)_{12}$ in complex with ColHMT, the authors should clarify why this observation supports the conclusion of N-to-C directional movement along the triple helix. Additionally, please explain why a C-to-N directional movement would be inconsistent with the data, as this is not immediately clear from the text. Please also discuss why not a similar behavior (i.e. complete unwinding of the triple helix upstream of the C-terminally bound ColH) was observed regarding the complex with (POG)10.

Line 203: "shows [a] dynamic "closed" state]" Please correct.

Line 204/Line 206: The authors correctly note that their findings "support" (line 104) the conclusion that ColH can adopt a ratcheted state only when bound to a partially unwound substrate. In light of this, the subsequent statement that "[t]hese results demonstrate that ColH exploits …" should be moderated, as the data support the claim but do not entirely demonstrate it.

Line 207: Please replace "unlaying" with "unwinding".

Line 211: The manuscript mentions "previous studies"; please include the appropriate citations to clarify which studies are being referenced.

Line 212: Please change to plural, i.e. "how bacterial collagenases recognize the collagen triple helix and exploit its geometry for processive stand-specific cleavage.

@ Discussion:
Please elaborate on how the presented results fit with the observed "open" conformation of bacterial collagenases reported by Eckhard et al (Ref. 3), Wang et al. (Ref.10), Ikeuchi et al. (Ref.15), designated in this manuscript as "wide-open" conformation. It would be very interesting to see a figure in which the dimensions of the different opening or closing states, respectively, are compared.

Line 218: The ColHMT-N-POG10 complex represents an "unproductive" substrate binding mode (resulting probably from the mis-staggered triple helix at this location). While this is unfortunate, this complex nonetheless illustrates how ColH could utilize the captured, but unwound substrate as "rail tracks" for its one-dimensional diffusion, reminiscent of DNA-binding clamps. Please change the text to reflect this conclusion by the authors.

Line 235-236: I would recommend the use of [cleavage] instead of [release]: "This captured bight is critical for translocation and reloading of the substrate, enabling [cleavage] of a Gly-X-Y tripeptide".

Line 238: "underscoring [the] mechanistic divergence between bacterial and mammalian enzymes." Please correct.

Lines 240-248: Please clarify that the authors present and propose here a new refined model of collagen degradation by bacterial collagenases.

Line 242: The term "unloads" implies active intervention by the collagenase. Was this the intended meaning? It seems more likely that the cleaved products dissociate due to reduced affinity for the active-site cleft.

Line 246: I suggest including a reference to Watanabe-Nakayama et al. (2016), who demonstrated catalytically productive unidirectional movement of ColG on collagen at the fibril level from C-to-N terminus, as this would provide useful context for the reader.

Where is Reference 26 cited?

Line 250: "was added [to remove the GST tag]"

Line 361: Please provide buffer/elution details regarding the ion-exchange chromatography.

Line 368: Please provide buffer details for the reconstitution of the peptide solutions.

Line 672: It would be improve clarity if the authors elaborated why the (hypothesized) N-terminally bound ColH molecule could not be observed by cryo-EM, while the C-terminally bound ColH was.

Line 507: The method used to assess collagenolytic activity may not be perfectly able to distinguish between gelatinolytic and collagenolytic activity, as the assay was performed at 37°C, a temperature at which singly-accessible collagen molecules on the surface of the fibril will already start to denature (doi: 10.1073/pnas.032307099).

Regarding the reproducibility of the data, although being only a minor issue, I would like to remark that I could numerically

not reproduce the RMSD deviations for bond lengths and bond angles for all reported EM structures using PHENIX. Please check.

Regarding the reported crystal structure for ColH CM, the Rwork/Rfree ratio (0.224/0.283) indicates overfitting of the density. Please revise your model.

Line 709:"as described in Extended Data Fig. 6, and Supplementary Videos 1 and 2", these references appear to be mis-placed.

Line 710: Please correct to "activator domain" (not accessory domain).

Line 711: Remove full stop after ColHWT.

Line 741: "as described in Extended Data Fig. 5 and [illustrated] in Supplementary Video 3"

Line820: Please correct to "PPG10".

Extended Data Fig. 1 & Extended Data Fig.4: Please provide also the intersection with FSC = 0.143 (map-to-map) and the corresponding resolution.

Version 1:

Reviewer comments:

Reviewer #1

(Remarks to the Author)
I appreciate the authors efforts to address all critiques raised in the first round of review. From this reviewers perspective the manuscript and models have been significantly improved. I support publication of this manuscript.

Reviewer #2

(Remarks to the Author)
The authors have made good efforts to address the reviewer comments and the manuscript has significantly improved. Some minor suggestions for the revised manuscript are as follows:

1. The author did not include an introduction to the collagenases of the two subfamilies (M9A and M9B) of the M9 family in the Introduction, which made the analysis and discussion of M9A and M9B collagenases in the Results and Discussion sections appear somewhat abrupt. Therefore, I suggest adding an introduction to M9A and M9B collagenases between lines 55 and 63, such as domain composition, characteristics of cleavage sites, structural features, etc.

2. L70-L72: The author compares the differences in cleavage characteristics of collagen peptide chain between M9 bacterial collagenases and M10 mammalian matrix metalloproteinases here. However, merely citing the description of M9B collagenase is insufficient (ref 13), as M9A collagenases also exhibit cleavage site specificity for Y-Gly bonds in collagen peptide chains releasing collagen tripeptide Gly–X–Y (J Agric Food Chem. 2025 73:22079-22088; Nat Commun. 2022 13:566; Appl Environ Microbiol. 2022 88:e0167721). There is also a similar deficiency in lines 181 to 182.

3. I suggest adding orange and purple color blocks in the blank area of Fig. 2f to represent the substrates Pz-peptide and collagen, respectively, so that readers can quickly grasp the activity information of the wild-type protein and its mutants toward the two substrates. This does not seem to contradict the suggestion from Reviewer 3.

Reviewer #3

(Remarks to the Author)
The quality and clarity of the revised manuscript have been significantly enhanced. The concerns raised in my previous comments have been thoroughly addressed, and I appreciate the considerate and detailed responses to my comments.

Upon review of the revised manuscript, four minor issues have arisen that need to be addressed prior to publication:
- Line 185: Please correct: "This distortion is critical for Zn2+-mediated polarization of the scissile carbonyl bond" instead of "chelation".
The catalytic zinc ion functions as a Lewis acid in metallopeptidases.
- The authors state in their response to the comment on former Lines 200–201 that ColH can translocate bidirectionally along the triple helix. To ensure consistency with this clarification, it would be appropriate to modify the wording in Lines 315–316 to "as it slides along the triple helix" instead of "from the N- to C-terminus."
- Supplementary Fig.16. Figure caption: Please provide the used PDB entry codes.
- Supplementary Fig.18: Please rephrase the figure caption to reflect that the presented model structure is the result of

homology modelling using MOE with complex ColHMT-(POG)10 (PDB: 9LQJ) as initial template.

Open Access This Peer Review File is licensed under a Creative Commons Attribution 4.0 International License, which permits use, sharing, adaptation, distribution and reproduction in any medium or format, as long as you give appropriate credit to the original author(s) and the source, provide a link to the Creative Commons license, and indicate if changes were made.
In cases where reviewers are anonymous, credit should be given to 'Anonymous Referee' and the source.
The images or other third party material in this Peer Review File are included in the article's Creative Commons license, unless indicated otherwise in a credit line to the material. If material is not included in the article's Creative Commons license and your intended use is not permitted by statutory regulation or exceeds the permitted use, you will need to obtain permission directly from the copyright holder.
To view a copy of this license, visit https://creativecommons.org/licenses/by/4.0/

Point-by-point responses

Reviewer #1 (Remarks to the Author):

In this manuscript Oki H et al. describe cryo-EM and X-ray structures of the H. histolytica collagenase ColH, a processive carboxytripeptidase. The structures presented reveal distinct states of collagen triple helix engagement and unwinding, providing unprecedented insight into the mechanism of collagen engagement and cleavage. Overall, this is a very well performed study that sheds new light on an interesting bacterial collagenase with potential for future engineering or inhibitor development. For the most part the manuscript is very clearly written, and should be easily understood even by a non-expert in the field. There are extensive supplemental and extended data figures that are well prepared to deliver the overall story. Overall, I am highly supportive of publication of this manuscript, as I feel the studies have been rigorously performed with appropriate analysis. I have only minor comments for the authors to consider before final publication.

Thank you for the positive evaluation of our manuscript. We greatly appreciate your generous assessment of the quality, clarity, and significance of our work, as well as the recognition of our careful data analysis and collection. We carefully addressed the comments below and made the corresponding changes in the revised manuscript to further improve the clarity of our work.

1. Line 98 - "Although low in amplitude, these motions generate an entrance of ~15Å, sufficient to capture a tropocollagen molecule in the central cavity". It may be nice to show a (supplemental/extended) figure with a tropocollagen molecule at scale next to the opening. It is hard to tell from the 3DVA alone just how large the opening would need to be in order for the "clamp" to open sufficiently to accommodate entry of tropocollagen.

Response: As suggested, we added Supplementary Fig. 6 showing a tropocollagen molecule [we used $(POG)_{10}$ as a model], viewed next to the 'open' conformation of the $ColH^{WT}$ structure (frame 9 of 3DVA component 1).

Comparison with the 'closed' conformation (frame 0 of 3DVA component 1) in the figure demonstrates that the partial opening is sufficient to allow entry of the collagen triple helix into the ColH CM ring.

Although cryo-EM 3DVA reveals a cleft opening that only marginally accommodates substrate, this motion likely represents the most populated trajectory under the imaged conditions. 3DVA captures dominant ensemble variability and does not preclude rarer, transient excursions that exceed the observed amplitude. Such low-occupancy openings may be functionally critical for substrate entry, distortion, or product release but remain under-represented in the cryo-EM ensemble.
We therefore interpret the 3DVA-derived opening as a lower bound on accessible cleft expansion rather than a strict physical limit.

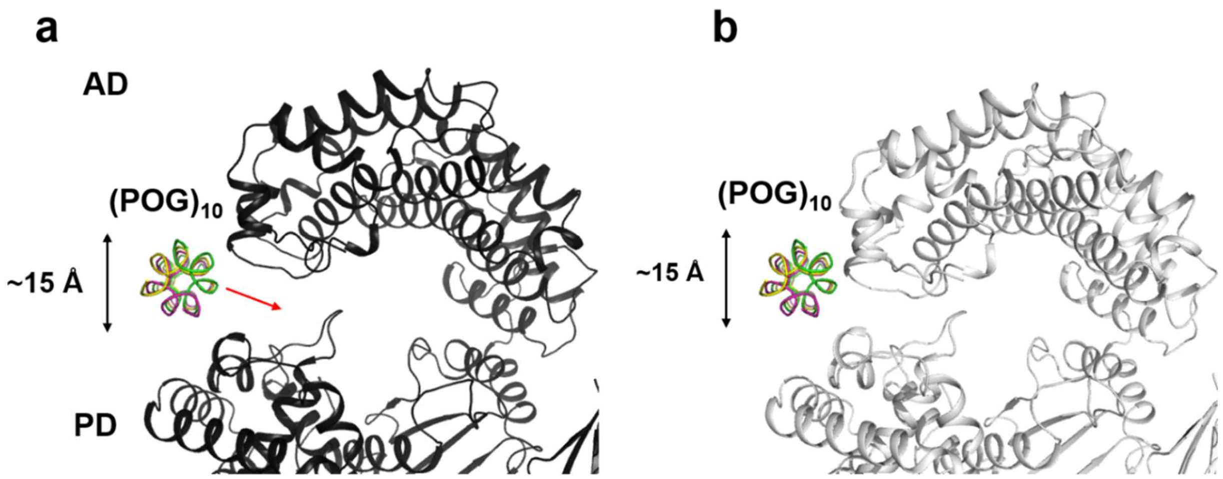

Supplementary Fig. 6. Partial opening of ColH^{WT} permits triple helix encirclement. **a** Enlarged view of gate region in the 'open' ColH^{WT} structure (frame 9 of 3DVA component 1). **b** Enlarged view of gate region in the 'closed' ColH^{WT} structure (frame 0 of 3DVA component 1). In both cases, a triple-helical collagen model peptide, $(POG)_{10}$, is positioned next to the gate region to represent tropocollagen.

2. While the resolutions reported in most of the cryo-EM experiments are indeed possibly suitable for placement of water molecules, I am skeptical of the placement and total number of water molecules included in the refined structures. In particular, in Figure 1B and 1D it is clear that the resolution in the Activator Domain is significantly lower due to inherent flexibility, yet water molecules are still modelled around the periphery of the activator domain. I am skeptical that these are truly water molecules, rather than simply "dust" generated by the automatic masking process in cryosparc. While well ordered water molecules in the protein interior with good stable hydrogen bonding are likely observed in the best resolved regions, the waters that are placed at the periphery of the protein (and especially in the Activator Domain) are skeptical at best. It may be worthwhile for the authors to carefully review the placement of water molecules.

Response: Thank you for this constructive comment. As noted, the resolution of the AD region in the consensus map is lower than that of other regions. This is due to the rigid-body motions of the domains mediated by the hinge region. Local refinement of the AD region substantially improved the EM map quality, yielding resolutions of 2.0–2.5 Å for most of the region, sufficient to visualise water molecules, with only terminal regions less clearly resolved.

Initially, we identified water molecules solely using the sharpened cryo-EM map. To avoid potential oversampling, we carefully re-evaluated water placement using both sharpened and unsharpened maps, considering hydrogen-bonding interactions, and focusing on regions with local resolutions better than 2.5 Å. Based on this reassessment, the coordinates (PDB codes: 9LRK, 9LQJ, 9WDC, 9LYI) were revised and updated in the PDB. Corresponding texts, tables, and figures were updated accordingly. Note that these revisions to water molecule placement do not affect the conclusions of the manuscript.

3. In addition to the point above, can the authors clearly see water molecules near the zinc active site? Including a supplemental figure demonstrating well resolved water molecules around the zinc active site would be ideal. From the placement of these water molecules is it possible to delineate the precise positioning of the water that will attack the peptide carbbonyl? This information could be very useful to those studying zinc metalloproteases more generally.

Response: We are grateful for the opportunity to clarify the structural features of the catalytic centre including the two coordinated water molecules. Accordingly, we added the relevant explanatory text (Lines 115–118 and 223–229) to the manuscript and Supplementary Fig. 7.

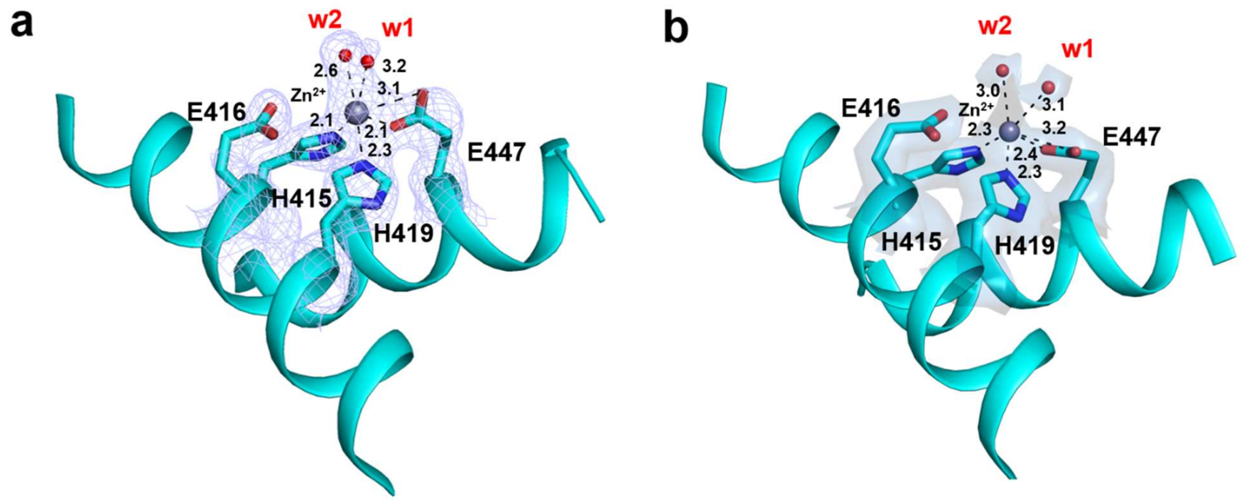

Supplementary Fig. 7. Comparison of catalytic centre of ColH^{WT} CM in the crystal and cryo-EM structures. **a** Amino acid residues (His415, His419, and Glu447) and water molecules (w1 and w2) coordinating with the zinc ion in the catalytic centre of ColH^{WT} CM in the crystal structure. 2Fo-Fc electron density maps (contoured at 1.0 σ) coloured light blue. **b** Amino acid residues (His415, His419, and Glu447) and water molecules (w1 and w2) coordinating with the zinc ion in the catalytic centre of ColH^{WT} CM in the cryo-EM structure. The cryo-EM map is contoured at 5.0 σ, and coloured light blue. The catalytic base Glu416 is also illustrated. All distances are shown in Å.

Our structural data from cryo-EM (9LRK) and crystallography (9CME), obtained at sufficient resolution to identify water molecules in the active site, consistently showed two water molecules chelated to the catalytic Zn^{2+} ion. The ion was coordinated by the conserved residues His415, His419, and the bidentate Glu447, as well as the two water molecules. Comparison with substrate-bound structures in which the trailing strand coordinates

the zinc (9LQJ, 9LRM, 9WDC, 9LYI) suggested that one of these two Zn^{2+}-bound water molecules in the $ColH^{WT}$ structures is displaced by the peptide carbonyl oxygen upon substrate engagement. The second water molecule likely serves as the catalytic water that, once deprotonated by Glu416, attacks the carbonyl carbon of the peptide bond while the zinc ion maintains a hexacoordinate geometry throughout the reaction; however, this could not be observed in the present study, likely due to mutation of Glu416 to Gln.

The following paragraph was added between Lines 115–118.
"At the catalytic centre, a Zn^{2+} ion was coordinated by the conserved residues, His415, His419, and the bidentate Glu447, as well as two water molecules (w1 and w2), as observed consistently in both the cryo-EM and X-ray crystal structures, adopting hexacoordinate geometry (Supplementary Fig. 7). Neither water molecule was positioned to be deprotonated by Glu416."

The following paragraph was added between Lines 224–230.
"At the catalytic centre, the scissile peptide carbonyl oxygen coordinated the Zn^{2+} ion, and no water molecules were observed near either the scissile bond or residue Gln416. The oxygen was located too close (3.2 Å) to the amide oxygen of Gln416, resulting in a non-productive pentacoordinate geometry and total loss of hydrolytic activities (Fig. 2f). Since the corresponding distance in a wild-type structure of *Grimontia* collagenase Ghcol in complex with $(GPO)_2$ is 4.6 Å[22], enough to evoke a substrate water molecule to the space, Glu416 in $ColH^{WT}$ may deprotonate a water molecule to initiate a nucleophilic attack."

4. Something seems weird in the methods section about box and pixel sizes. Line 386 - "with a box size of 256 pixels (1.86 Å/pix) ... particles were re-extracted with a box size of 256 pixels at 0.87 Å/pix". I think what the authors mean to say is that particles were originally extracted at a box size of ~550 pixels and downsampled to a box size of 256 pixels (1.87Å) before initial rounds of 2D classification? I don't see how the particles could be extracted with the same box size and different pixel sizes unless the micrographs themselves were binned at some point, or during extraction the particles were also downsampled. Please clarify in all sections throughout the methods.

Response: We appreciate your concern regarding the clarity of the particle-extraction workflow. In the $ColH^{WT}$ dataset, particles were initially extracted after Fourier cropping (down-sampling) to 128 pixels at 1.86 Å/pixel. These down-sampled particles were used for initial 2D classification, ab initio reconstruction, and heterogeneous refinement to remove junk particles and retain only high-quality candidates. The selected particles were then re-extracted at the original box size (256 pixels) and pixel size (0.87 Å/pixel); and used for subsequent homogeneous refinement, non-uniform refinement, and other subsequent analyses.
We have clarified in the Methods section that this same general approach—down-sampling for the initial extraction and classification, followed by re-extraction at the original box and pixel sizes—was employed for other datasets as well. Accordingly, we revised the corresponding figures (Supplementary Figs. 1, 8, 9, 14, and 20).

5. Line 118 - "unwinding its terminal three triplets in an N-to-C direction". This statement confused me when I first read it. Couldn't one argue that the actual unwinding occurs from a C-to-N direction? Figure 5 makes this a little more clear, but at this point in the manuscript I was slightly confused by this wording and the diagram shown in Figure 2. Please see if revision makes sense, or this statement is accurate. As a reader I may be getting confused between "unwinding" and "translocation".

Response: Thank you for this insightful comment and acknowledge the potential confusion caused between 'unwinding' and 'translocation' in the original wording. The directionality of the unwinding process itself cannot be conclusively determined at this stage. Thus, we should have stated that $ColH^{MT}$ may translocate from the N- to the C-terminus and unwind the three C-terminal triplets of $(POG)_{10}$.

Although $ColH^{MT}$ adopts a ring-shaped architecture and is capable of topologically entrapping $(POG)_{10}$ at any position, we did not observe it engaging in the opposite orientation or stabilising an unwound N-terminal region. These results suggest that once $ColH^{MT}$ recognises a region that is more readily unwound [in the present case, the C-terminus of $(POG)_{10}$], it adopts a stable conformation at that site, becomes arrested, and does not move in the reverse direction thereafter.

To address these points, we have revised the text to distinguish between substrate translocation and unwinding. We believe these changes resolve the ambiguity.

Revised (Lines 132–139):
"Recognition was unidirectional, with N-to-C binding of the unwound region from the front to the back face of $ColH^{MT}$ (Fig. 2b, c). Neither reverse-direction recognition nor recognition involving unwinding of the central region or N-terminal side of $(POG)_{10}$ was detected in the cryo-EM analysis. Assuming that $ColH^{MT}$ can topologically entrap any site of $(POG)_{10}$ within its CM ring, this was not initially predicted. A previous study indicated that unwinding by M9B collagenase tends to be a local process[8]; therefore, $ColH^{MT}$ likely recognises a readily or sufficiently unwound region during N-to-C translocation, adopts a stable conformation through unidirectional binding at that site, and subsequently becomes arrested."

6. Line 149 - it may be good to include a reference for the role of Calcium coordination in catalysis.

Response: Thank you for this suggestion. We have added a relevant reference (ref. 16; Eckhard et al. *J. Biol. Chem.*, 2013) describing the role of calcium coordination in catalysis (Line 200).

Reviewer #2 (Remarks to the Author):

The collagenolytic mechanism of M9 collagenases has been extensively studied. The collagen triple helix is the minimal structural unit of collagen, and it is generally believed that this triple helical molecule must be locally unwound to release a single peptide chain into the catalytic cavity before hydrolysis (2012 NSMB; 2022 NC; 2024 PNAS). However, direct evidence showing that any M9 collagenase actively drives this unwinding process has been lacking. In this context, the work by Oki et al. provides valuable structural and biochemical insights. The authors cleverly utilized a collagen synthetic peptide that forms a stable triple helix in solution and resolved cryo-EM structures of M9B collagenase ColH in complex with POG10 in both its triple helical and unwound forms. Combined with biochemical assays, this work explores the unwinding mechanism of the collagen triple helix by ColH. Despite these important contributions, there are several concerns that need to be addressed before publication.

Thank you for your thoughtful summary of our work and for recognising the significance of our structural and biochemical analyses of M9 collagenase ColH. While previous studies have reported complexes primarily with small-molecule inhibitors/peptides, to our knowledge, this is the first report of a complex with a triple-helical collagen substrate. We appreciate your positive evaluation of our experimental strategy, particularly the use of stable triple-helical collagen peptides; and the acknowledgment of the new mechanistic insights our study provides into how ColH unwinds and processes the collagen triple helix.
We carefully addressed the concerns raised below and have made the corresponding changes in the revised manuscript to further clarify and strengthen our study.

1. From a three-dimensional structural perspective, the W431 residue is far from the catalytic center of ColH. However, as shown in Fig. 2g, the W431 mutation to alanine not only significantly reduced the activity of collagen but also almost completely lost the activity of Pz peptide. There is no doubt that this phenomenon is counterintuitive. Although the author provided relevant explanations in L148-L149, the significance of the W431 residue of ColH in triple helix unwinding or catalysis deserves to be reconsidered. The solution to this question would benefit from circular dichroism spectroscopy analysis of mutant W431A and even other mutants to determine that the decreased collagen-binding ability of the mutants is caused by amino acid replacement rather than structural alteration.
In addition, given the significance of the W431 residue in the triple helix unwinding process, the authors are encouraged to resolve the complex structure of W431A and POG10.

Response: As suggested, we performed circular dichroism spectroscopy of all mutants, including W431A (Supplementary Fig. 17). The obtained spectra were consistent with that of the wild-type ColH, indicating that the mutations introduced in this study do not cause any detectable structural alterations.

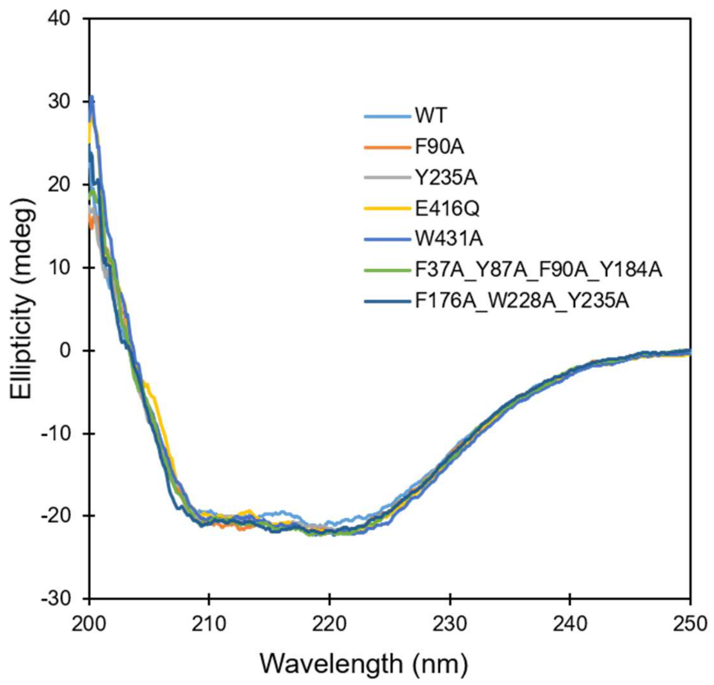

Supplementary Fig. 17. Circular dichroism (CD) spectra of ColH^{WT} and its variants. Far-UV CD spectra of the wild-type protein and the indicated variants were measured using a Jasco J-720 spectropolarimeter at 200–250 nm, and ellipticity is shown in millidegrees (mdeg).

Trp431 is located close to the catalytic centre (within 10 Å from zinc to the indole ring) via the calcium-binding loop. Our cryo-EM analysis of the ColH^{MT}–(POG)$_{10}$ complex clearly demonstrated that the indole ring of Trp431 plays a crucial role in stabilising Pro25 (P2) via a CH–π interaction (Fig. 2e, g, h). We constructed a structural model of ColH with Pz-peptide based on the ColH^{MT}–(POG)$_{10}$ complex, which indicated that this interaction also contributes to the loss of Pz-peptide activity, as the Pro1 residue of the Pz-peptide is similarly positioned in the S2 subsite and interacts with Trp431 (Supplementary Fig. 18). These results from structural and mutational analyses further substantiate the critical role of Trp431 in ensuring stable substrate binding and correct orientation within the active-site cleft for efficient hydrolysis.
To explain these results, we included additional text and figures (Supplementary Figs. 17 and 18).

Revised (Lines 211–223):
"Strikingly, the substitution of Trp431 with alanine resulted in a dramatic decrease (~95%) in collagenolytic activity and almost complete loss of enzymatic activity toward Pz-peptide, comparable to that observed for the catalytically inactive E416Q mutant (Fig. 2f). Circular dichroism (CD) measurements confirmed comparable structures between the mutants and ColH^{WT} (Supplementary Fig. 17). Trp431 is located close to the catalytic centre (within 10 Å from zinc to the indole ring) via the calcium-binding loop. Cryo-EM analysis of the ColH^{MT}–(POG)$_{10}$ complex clearly demonstrated that the indole ring of Trp431 plays a crucial role in stabilising Pro25 (P2) via a CH–π interaction (Fig. 2g). A structural model of ColH with Pz-peptide based on the ColH^{MT}–(POG)$_{10}$

complex indicated that this interaction is also responsible for the loss of Pz-peptidase activity, as the Pro1 residue of Pz-peptide is predicted to be in the S2 subsite and interacts with Trp431 (Supplementary Fig. 18). These results further substantiate the critical role of Trp431 in ensuring stable substrate binding and correct orientation within the active-site cleft for efficient hydrolysis."

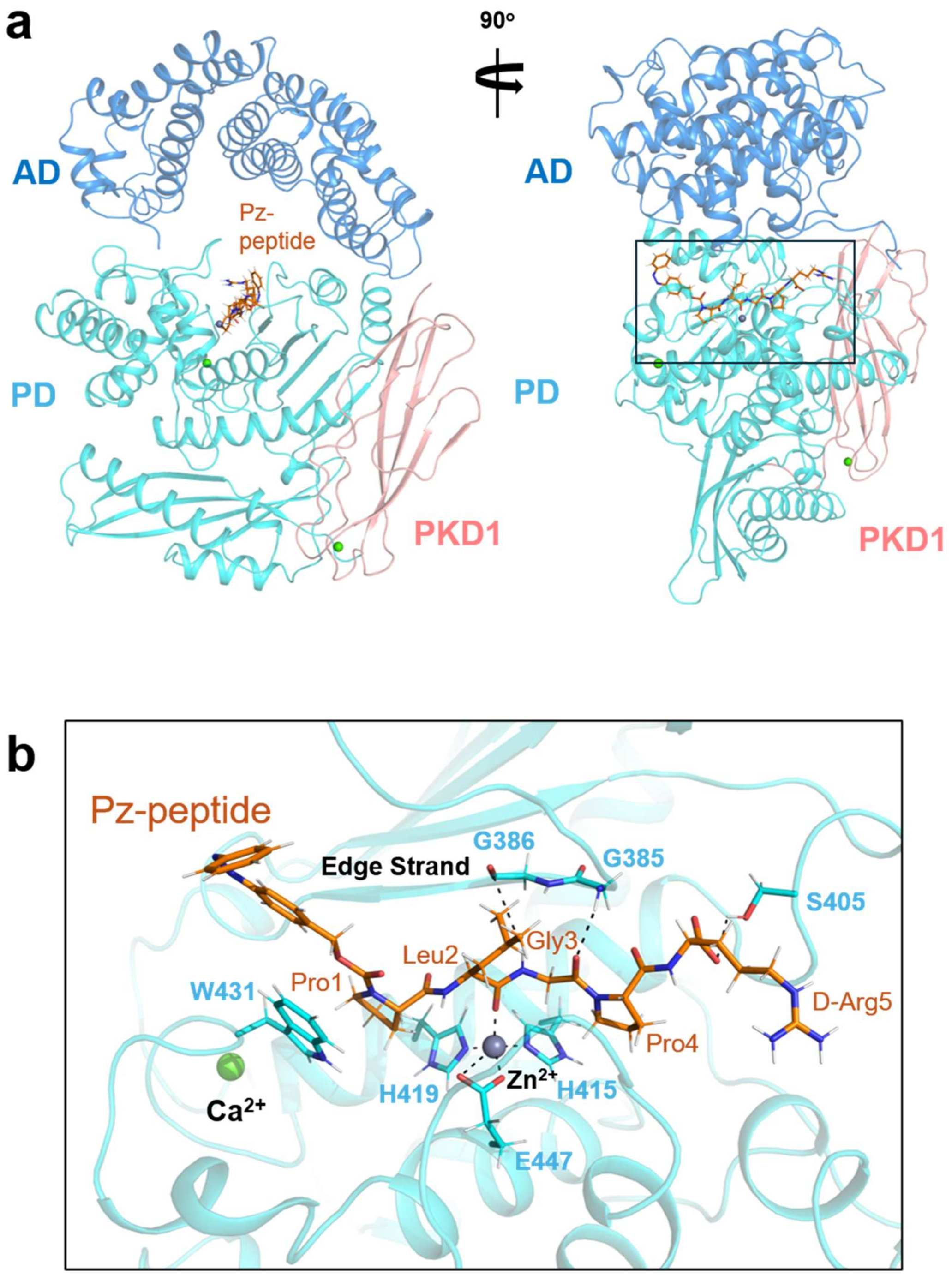

Supplementary Fig. 18. Model structure of ColH^{WT}–Pz-peptide complex. **a** Overall structure of ColH^{WT} and Pz-peptide complex. **b** Enlarged view of active site in the complex with Pz-peptide. Pz-peptide bound to PD coordinates the zinc ion via the carbonyl oxygen of Leu2 (P1), and the adjacent Pro1 (P2) forms the CH–π and hydrophobic interactions with Trp431. The orange columns represent the carbon atoms of Pz-peptide.

We performed cryo-EM analysis of the complexes between ColH containing the W431A substitution and $(POG)_{10}$ under the same experimental condition as the $ColH^{MT}$–$(POG)_{10}$ complex. The 2D class images (Fig. R1) and reconstruction at 3.0 Å resolution yielded a structure reminiscent of ligand-free $ColH^{WT}$ (left panel in Fig. R2). In contrast to $ColH^{MT}$-$(POG)_{10}$ complexes, in which a clear EM density of the trailing strand is observed at the active-site cleft (right panel in Fig. R2), no residual density corresponding to the peptide substrate was detected in the active-site cleft of the W431A mutant (left panel in Fig. R2). Similarly, no such residual density map was observed at any of the exosites of the AD. This is likely due to the W431A mutation, which appears to have markedly reduced binding affinity.

While further optimisation of conditions might provide additional insights into complex structure, this is beyond the scope of the present study.

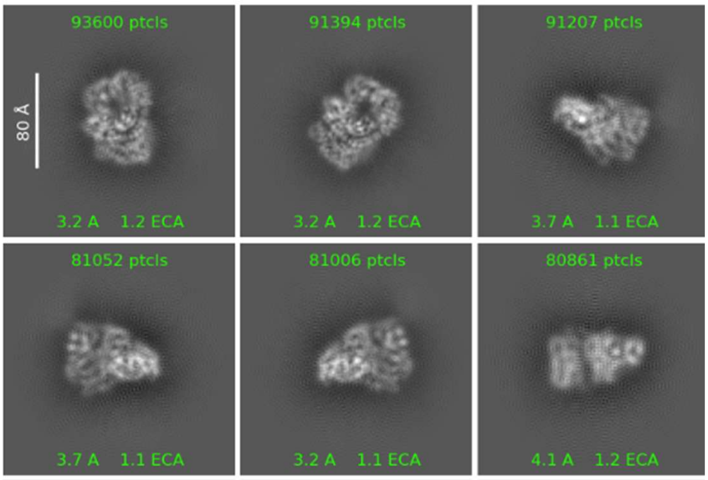

Figure R1. 2D class images of ColH_W431A-$(POG)_{10}$ complex.

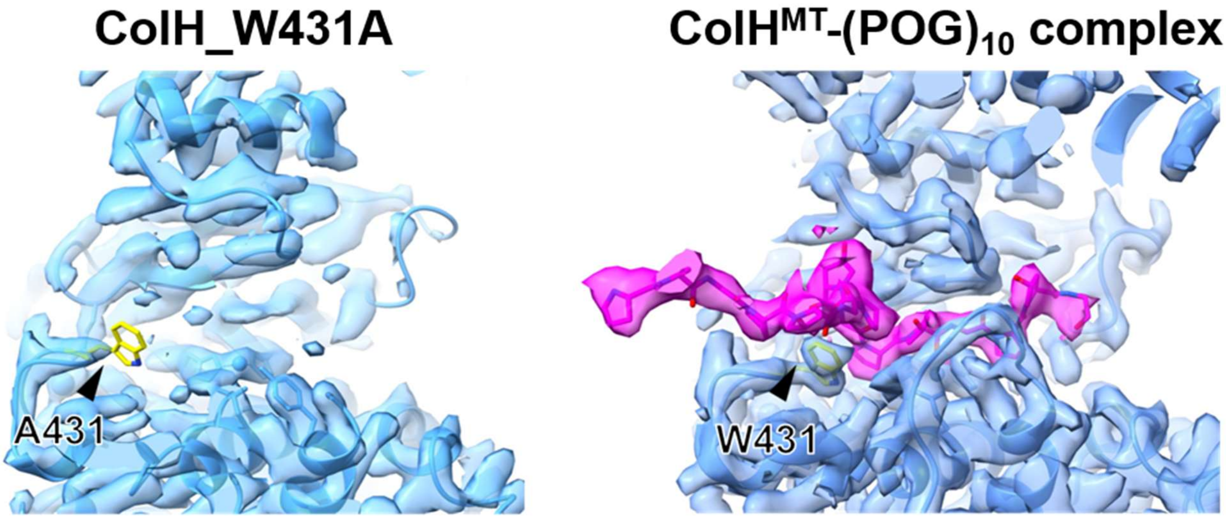

Figure R2. Active-site clefts of the PD catalytic subdomains of ColH_W431A (left panel) and the $ColH^{MT}$–$(POG)_{10}$ complex (right panel). In the $ColH^{MT}$–$(POG)_{10}$ complex, the EM density corresponding to the trailing strand of $(POG)_{10}$ is coloured magenta.

2. L146: What kind of interaction exists between W431 and P25 and P22? It is recommended a close-up view of the relative positions or interactions between relevant amino acids in Fig. 2e.

Response: As suggested, we added a description of the interactions to the text (Lines 192–195), along with a figure (Fig. 2g) providing a close-up view of W431 and Pro25 (P2) and Pro22 (P5).

Revised (Lines 192–195):

"On the opposite side, Trp431 interacted with Pro25 (P2) and the Cα–H of Gly24 (P3) through CH–π interactions and, together with Tyr491, interacts with Pro22 (P5) via nonpolar contacts and shape complementarity, thereby stabilising the three-residue bight (Fig. 2g)."

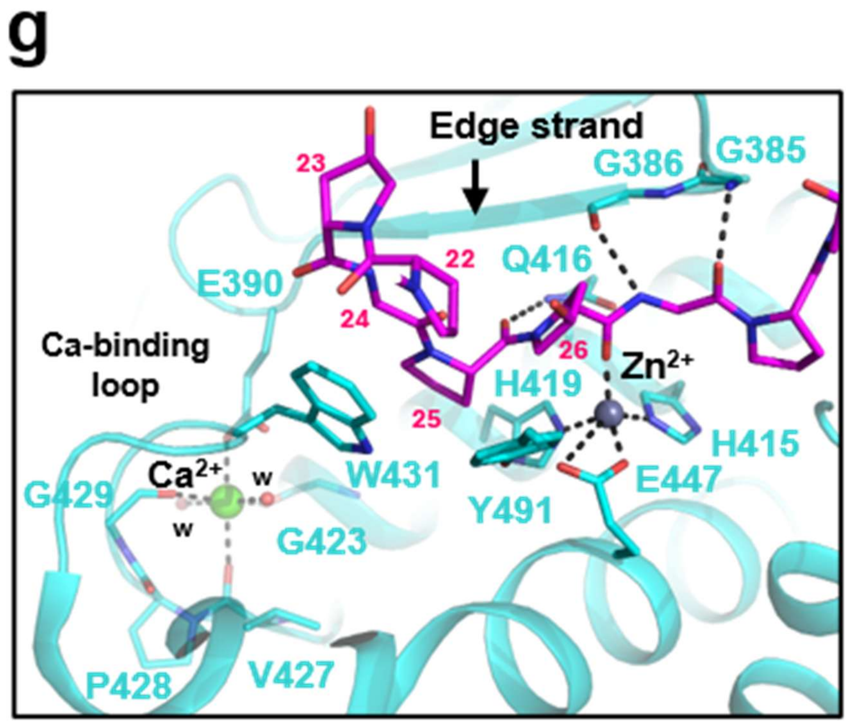

Fig. 2. ColH binds to unwound C-terminus of $(POG)_{10}$. g A magnified view of the active-site cleft around Trp431 (shown as a stick model), located on a Ca-binding loop.

3. In the complex structure of ColH and POG12, why is the triple helix form of POG12 not visible? Can the author make a reasonable speculation and elaborate on it in the text? Is it related to its own properties of POG12 or did the enzyme molecule ColH mediate the complete unwinding of the triple helix molecule?

Response: Thank you for this important question. The triple-helical form of $(POG)_{12}$ was unexpectedly not observed in the $ColH^{MT}$–$(POG)_{12}$ complex. Since the triple helix of $(POG)_{12}$ is more stable than that of $(POG)_{10}$, this phenomenon is unlikely to result from the intrinsic properties of $(POG)_{12}$. Instead, as shown in Fig. 4, we propose that two $ColH^{MT}$ molecules (i.e. $ColH^{MT}$-N and $ColH^{MT}$-C) cooperatively unwound the helix completely. For $(POG)_{10}$, the peptide was too short for the second $ColH^{MT}$ molecule ($ColH^{MT}$-N) to bind stably (Supplementary Fig. 19). In contrast, for $(POG)_{12}$, stable binding of the second $ColH^{MT}$ molecule allowed unwinding to proceed in the N-to-C direction (although C-to-N translocation may also be possible), thereby promoting overall unwinding.

Once fully unwound, the two molecules are difficult to analyse collectively owing to their orientational flexibility or the possible dissociation of one molecule from the unwounded peptides. Since $ColH^{MT}$-N and $ColH^{MT}$-C are expected to recognise the unwounded peptide segments similarly, only an averaged structure is obtainable. We have revised the text (Lines 283–285) accordingly to incorporate this point.

Revised (Lines 283–285):
"Once fully unwound, the two molecules are difficult to analyse collectively owing to their orientational flexibility or possible dissociation of one molecule from the unwound peptides."

4. The conservation of key sites (W431, F90, Y235, etc.) related to triple helix unwinding throughout the M9 family collagenases (M9A and M9B) needs to be explored.

Response: We appreciate the suggestion. We examined the conservation of key residues at the Exo^{Lead} and Exo^{Middle} sites across the M9 family collagenases (both M9A and M9B subfamilies). Interestingly, sequence alignment and structural comparison revealed that the residues at Exo^{Lead} are highly conserved among M9 family members, suggesting a common mechanistic role in collagen triple-helix recognition and unwinding at this site. In contrast, the residues at Exo^{Middle} are not as well conserved, even within the M9B subfamily.

We added a description of these results in the revised manuscript (Lines 147–164 in the main text and Lines 585–591 in the methods section), and also added figures (Supplementary Figs. 12 and 13).

Revised (Lines 147–164):
"Partial unfolding at the C-terminus allows the leading and middle strands of $(POG)_{10}$ to bind to two AD exosites (Exo^{Lead} and Exo^{Middle}; Fig. 2b, e). Both sites engage two contiguous triplets (Pro25–…–Gly30) via hydrophobic clefts. Proline residues at X positions (Pro25 and Pro28) form contacts with aromatic residues—Phe37, Tyr87, Phe90, and Tyr184 at Exo^{Lead}; Phe176, Trp228, and Tyr235 at Exo^{Middle}—through hydrophobic and CH–π interactions (Fig. 2e). Alanine substitution of these residues significantly reduced type I collagen hydrolysis but not 4-phenylazobenzyloxycarbonyl-Pro-Leu-Gly-Pro-D-Arg (Pz-peptide) hydrolysis, highlighting their role in triple-helix recognition and unwinding (Fig. 2f). Mutations in the Exo^{Lead} residues caused a substantially greater

reduction in collagenolytic activity than those in Exo^{Middle} (~90% versus ~60%), suggesting that Exo^{Lead} plays a more critical role in collagen recognition and unwinding. Sequence and structural comparisons with M9A and M9B collagenases showed that Exo^{Lead} residues are strictly or highly conserved across these enzymes (Supplementary Figs. 12 and 13). Consistently, their importance for collagenolytic activity in both subfamilies has been confirmed in previous mutational studies[7, 8]. In contrast, the aromatic residues comprising Exo^{Middle} in ColH are intriguingly not as well conserved within the M9 family. In the M9B subfamily, although Y235 and W228 are conserved, F176 is not, whereas in M9A, only the residue equivalent to W228 is conserved. This relatively low level of conservation may underlie the variability in substrate binding at this exosite."

Revised (Lines 585–591, Methods section):
"Amino-acid sequences from three previously determined structures and one newly determined structure —two M9A collagenases (Ghcol[15] and VhaC[7]) and two M9B collagenases (ColG[5] and ColH)—were aligned using Clustal Omega[42]. Structural alignment of their AD and catalytic subdomain was performed in Coot 0.9.8.93 EL[43] to identify structurally equivalent residues and refine the sequence alignment. Secondary-structure elements for ColH were assigned using DSSP[44]."

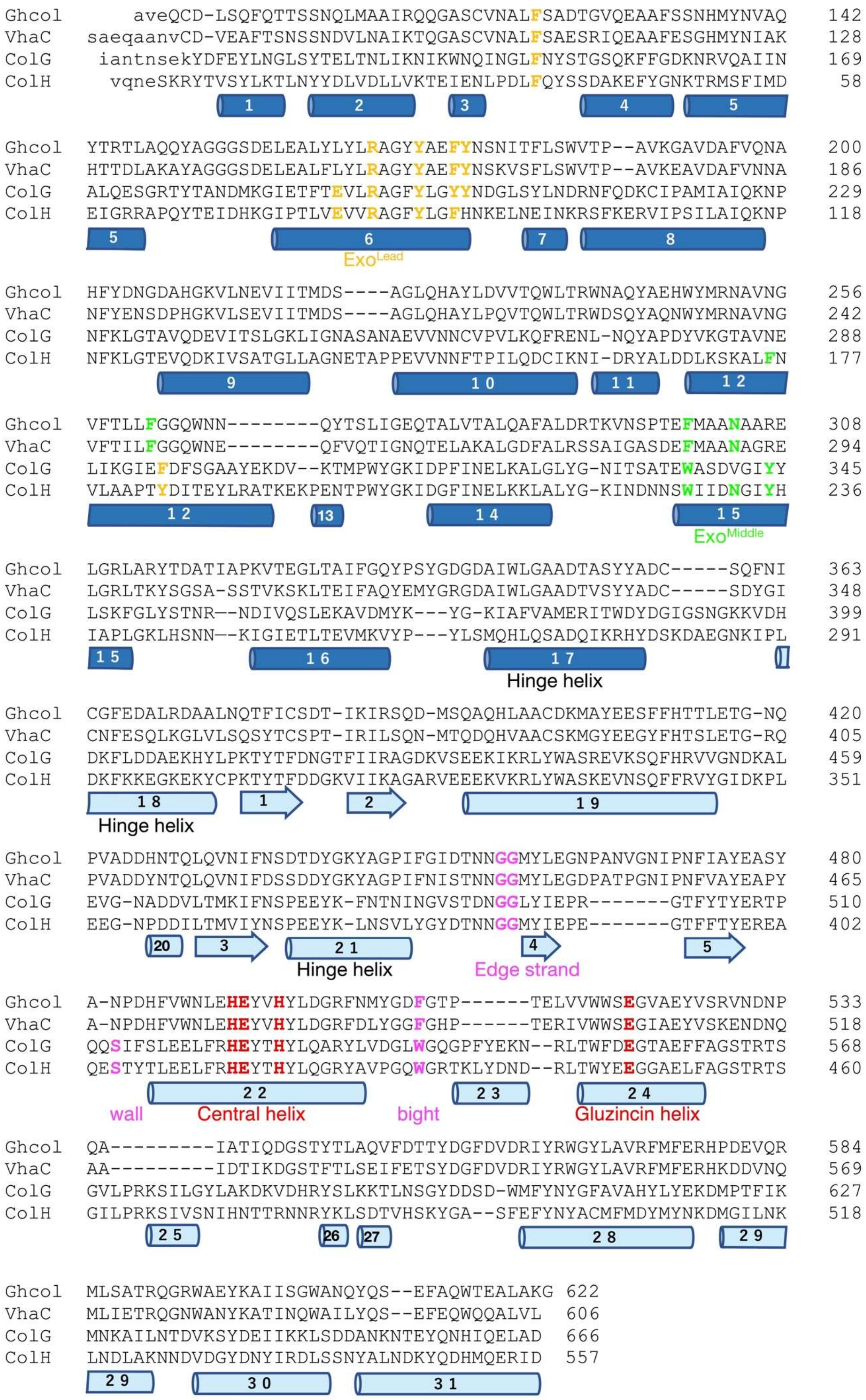
Ghcol aveQCD-LSQFQTTSSNQLMAAIRQQGASCVNALFSADTGVQEAAFSSNHMYNVAQ 142
VhaC saeqaanvCD-VEAFTSNSSNDVLNAIKTQGASCVNALFSAESRIQEAAFESGHMYNIAK 128
ColG iantnsekYDFEYLNGLSYTELTNLIKNIKWNQINGLFNYSTGSQKFFGDKNRVQAIIN 169
ColH vqneSKRYTVSYLKTLNYYDLVDLLVKTEIENLPDLFQYSSDAKEFYGNKTRMSFIMD 58
1 2 3 4 5
Ghcol YTRTLAQQYAGGGSDELEALYLYLRAGYYAEFYNSNITFLSWVTP--AVKGAVDAFVQNA 200
VhaC HTTDLAKAYAGGGSDELEALFLYLRAGYYAEFYNSKVSFLSWVTP--AVKEAVDAFVNNA 186
ColG ALQESGRTYTANDMKGIETFTEVLRAGFYLGYYNDGLSYLNDRNFQDKCIPAMIAIQKNP 229
ColH EIGRRAPQYTEIDHKGIPTLVEVVRAGFYLGFHNKELNEINKRSFKERVIPSILAIQKNP 118
5 6 7 8
ExoLead
Ghcol HFYDNGDAHGKVLNEVIITMDS----AGLQHAYLDVVTQWLTRWNAQYAEHWYMRNAVNG 256
VhaC NFYENSDPHGKVLSEVIITMDS----AGLQHAYLPQVTQWLTRWDSQYAQNWYMRNAVNG 242
ColG NFKLGTAVQDEVITSLGKLIGNASANAEVVNNCVPVLKQFRENL-NQYAPDYVKGTAVNE 288
ColH NFKLGTEVQDKIVSATGLLAGNETAPPEVVNNFTPILQDCIKNI-DRYALDDLKSKALFN 177
9 10 11 12
Ghcol VFTLLFGGQWNN--------QYTSLIGEQTALVTALQAFALDRTKVNSPTEFMAANAARE 308
VhaC VFTILFGGQWNE--------QFVQTIGNQTELAKALGDFALRSSAIGASDEFMAANAGRE 294
ColG LIKGIEFDFSGAAYEKDV--KTMPWYGKIDPFINELKALGLYG-NITSATEWASDVGIYY 345
ColH VLAAPTYDITEYLRATKEKPENTPWYGKIDGFINELKKLALYG-KINDNNSWIIDNGIYH 236
12 13 14 15
ExoMiddle
Ghcol LGRLARYTDATIAPKVTEGLTAIFGQYPSYGDGDAIWLGAADTASYYADC-----SQFNI 363
VhaC LGRLTKYSGSA-SSTVKSKLTEIFAQYEMYGRGDAIWLGAADTVSYYADC-----SDYGI 348
ColG LSKFGLYSTNR--NDIVQSLEKAVDMYK---YG-KIAFVAMERITWDYDGIGSNGKKVDH 399
ColH IAPLGKLHSNN--KIGIETLTEVMKVYP---YLSMQHLQSADQIKRHYDSKDAEGNKIPL 291
15 16 17
Hinge helix
Ghcol CGFEDALRDAALNQTFICSDT-IKIRSQD-MSQAQHLAACDKMAYEESFFHTTLETG-NQ 420
VhaC CNFESQLKGLVLSQSYTCSPT-IRILSQN-MTQDQHVAACSKMGYEEGYFHTSLETG-RQ 405
ColG DKFLDDAEKHYLPKTYTFDNGTFIIRAGDKVSEEKIKRLYWASREVKSQFHRVVGNDKAL 459
ColH DKFKKEGKEKYCPKTYTFDDGKVIIKAGARVEEEKVKRLYWASKEVNSQFFRVYGIDKPL 351
18 1 2 19
Hinge helix
Ghcol PVADDHNTQLQVNIFNSDTDYGKYAGPIFGIDTNNGGMYLEGNPANVGNIPNFIAYEASY 480
VhaC PVADDYNTQLQVNIFDSSDDYGKYAGPIFNISTNNGGMYLEGDPATPGNIPNFVAYEAPY 465
ColG EVG-NADDVLTMKIFNSPEEYK-FNTNINGVSTDNGGLYIEPR-------GTFYTYERTP 510
ColH EEG-NPDDILTMVIYNSPEEYK-LNSVLYGYDTNNGGMYIEPE-------GTFFTYEREA 402
20 3 21 4 5
Hinge helix
Edge strand
Ghcol A-NPDHFVWNLEHEYVHYLDGRFNMYGDFGTP------TELVVWWSEGVAEYVSRVNDNP 533
VhaC A-NPDHFVWNLEHEYVHYLDGRFDLYGGFGHP------TERIVWWSEGIAEYVSKENDNQ 518
ColG QQSIFSLEELFRHEYTHYLQARYLVDGLWGQGPFYEKN--RLTWFDEGTAEFFAGSTRTS 568
ColH QESTYTLEELFRHEYTHYLQGRYAVPGQWGRTKLYDND--RLTWYEEGGAELFAGSTRTS 460
22 23 24
wall
Central helix
bight
Gluzincin helix
Ghcol QA---------IATIQDGSTYTLAQVFDTTYDGFDVDRIYRWGYLAVRFMFERHPDEVQR 584
VhaC AA---------IDTIKDGSTFTLSEIFETSYDGFDVDRIYRWGYLAVRFMFERHKDDVNQ 569
ColG GVLPRKSILGYLAKDKVDHRYSLKKTLNSGYDDSD-WMFYNYGFAVAHYLYEKDMPTFIK 627
ColH GILPRKSIVSNIHNTTRNNRYKLSDTVHSKYGA--SFEFYNYACMFMDYMYNKDMGILNK 518
25 26 27 28 29
Ghcol MLSATRQGRWAEYKAIISGWANQYQS--EFAQWTEALAKG 622
VhaC MLIETRQGNWANYKATINQWAILYQS--EFEQWQQALVL 606
ColG MNKAILNTDVKSYDEIIKKLSDDANKNTEYQNHIQELAD 666
ColH LNDLAKNNDVDGYDNYIRDLSSNYALNDKYQDHMQERID 557
29 30 31

Supplementary Fig. 12. Amino acid sequence alignment among mature bacterial collagenases. Sequences of AD and PD (or catalytic subdomain) from *Grimontia hollisae* (M9A, Ghcol, GenBank BAK39964), *Vibrio harveyi* (M9A, VhaC, GenBank WP_047516938), and *Hathewaya histolytica* (M9B, ColG, GenBank BAA77453; and M9B, ColH, GenBank BAA06251) were aligned, and the gaps ('-') were manually adjusted by structural comparison. N-terminal residues in lower case were not observed. Substrate-binding residues for the leading, middle, and trailing strand are coloured yellow, green, and magenta, respectively. Residues at the catalytic centre are coloured red. Secondary structures of ColH are illustrated by cylinders and arrows for helices and sheets, respectively. The colouring is the same as the structural illustration in Fig. 1d.

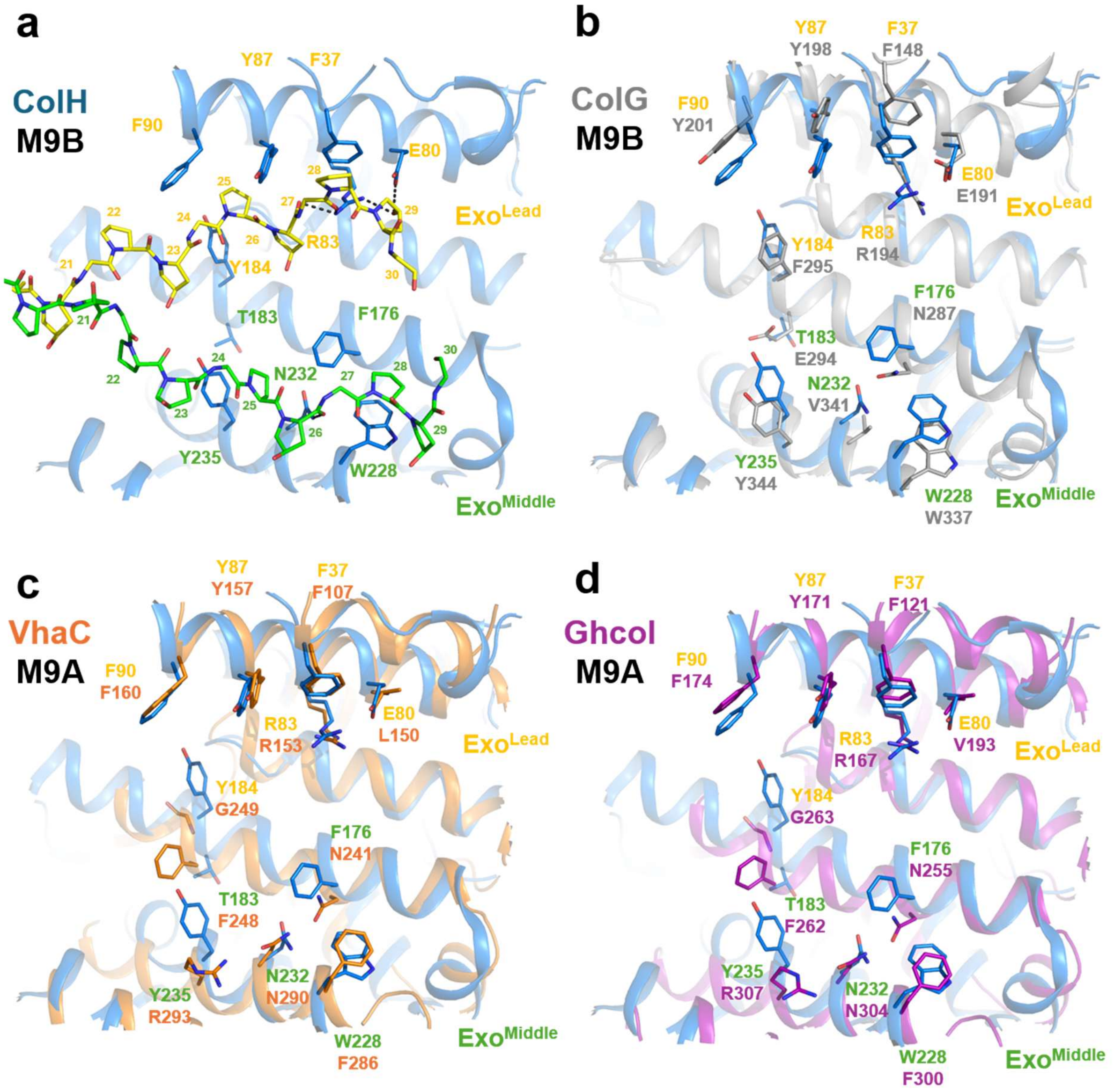

Supplementary Fig. 13. Structural comparison of the AD exosites of M9 family collagenases. **a** Magnified view of Exo^{Lead} and Exo^{Middle} of ColH^{MT}–(POG)$_{10}$ complex from *H. histolytica* (this study). **b** Magnified view of Exo^{Lead} and Exo^{Middle} of ColG from *H. histolytica* (PDB ID: 2Y3U). **c** Magnified view of Exo^{Lead} and Exo^{Middle} of VhaC from *V. harveyi* (PDB ID: 7ESI). **d** Magnified view of Exo^{Lead} and Exo^{Middle} of Ghcol from *G. hollisae* (PDB ID: 8JT1).

5. The proposed mechanism is mainly inferred from structural data, lacking direct kinetic or biochemical evidence. The discussion should explicitly acknowledge this limitation.

Response: We appreciate the suggestion. We added the following research limitation in the Discussion section.

Discussion section (Lines 343–344):
"Nonetheless, this model is mainly inferred from structural data, lacking direct kinetic or biochemical evidence; it must therefore be interpreted with caution and validated in future experimental studies."

6. The feasibility of ColH fully wrapping around a single collagen chain within densely packed fibrils is questionable. The authors should further discuss how ColH might act on native collagen substrates, going beyond the simple statement that ColH targets only partially unwound or damaged regions of collagen fibrils. In addition, the potential auxiliary roles of PKD and CBD domains in substrate recognition, binding, and unwinding should be considered to strengthen the physiological relevance of the proposed mechanism.

Response: We greatly appreciate the suggestion. Full-length ColH was used for cryo-EM visualisation; however, the PKD2–CBD region was not resolved in the final reconstruction. In contrast, SAXS analysis revealed that this collagen-binding segment extends from the catalytic module (Ohbayashi et al. *Biophys. J.* **104**, 1538–1545, 2013), indicating substantial conformational flexibility in solution and supporting its role as a flexible collagen-binding extension. Functionally, the PKD2–CBD region binds collagen fibrils with high affinity (Kd ≈ 0.34 μM) (Matsushita et al. *J Biol Chem.* **273**, 3643–3648, 1998), and enhances the degradation of native collagen fibrils by the ColH CM (Matsushita et al. *J. Biol. Chem.* **273**, 3643–3648, 1998). Previous studies have shown that the CBD binds unidirectionally to under-twisted regions of the triple helix (Philominathan et al. *J. Biol. Chem.* **284**, 10868–10876, 2009; Philominathan et al. *Protein Sci.* **21**, 1554–1565, 2012), whereas the PKD domain promotes fibril expansion and swelling without disrupting the integrity of the triple helix (Wang et al. *J. Biol. Chem.* **285**, 14285–14291, 2010).

These findings suggest that, within densely packed fibrils where steric constraints prevent the catalytic module from fully wrapping around a single tropocollagen molecule, the PKD2–CBD segment may function as an auxiliary tether that recognises locally distorted or partially unbundled regions of collagen. By transiently anchoring ColH to such hypersensitive sites, the PKD2–CBD region may guide the triple helix into the catalytic cleft, facilitating localised unwinding and cleavage. This auxiliary targeting mechanism is analogous to how transcription factors position RNA polymerase on DNA and likely enhances catalytic efficiency by enabling surface diffusion and selective engagement of vulnerable sites, rather than relying solely on random encounters with an insoluble substrate. In addition, consistent with a previous study (Wang et al. *Nat. Commun.* **13**, 566, 2022), PKD2–CBD may bind to regions that promote PD-mediated telopeptide cleavage, thereby exposing the tropocollagen for subsequent enzymatic processing.

We added a description of this valuable topic in the revised manuscript (Lines 344–353 in the discussion section).

Revised (Lines 346–354):

"Additionally, the present study leaves the unresolved question of how ColH overcomes steric constraints within densely packed collagen fibrils. The PKD2–CBD region of the collagenase may act as an auxiliary tether that recognises locally distorted or partially unbundled collagen regions[27, 28]. By transiently anchoring ColH to these hypersensitive sites, the PKD2–CBD region may help position the triple helix into the catalytic cleft, thereby facilitating localised unwinding and cleavage. This auxiliary targeting strategy may enhance catalytic efficiency by enabling surface diffusion and selective engagement of vulnerable sites, rather than relying solely on random encounters with an insoluble substrate. Nevertheless, the precise mechanism by which ColH achieves efficient fibril hydrolysis remains unclear, which will require further investigation."

Reviewer #3 (Remarks to the Author):

The manuscript by Oki and colleagues entitled "Bacterial Collagenase Harnesses Collagen Geometry for Processive Cleavage" reports on a study that investigates the mechanism of how bacterial collagenases unwind and cleave collagen. Collagen's triple-helical fold and high content of (hydroxy-)proline render the molecule extremely stable and resistant to enzymatic proteolysis within the context of the extracellular matrix. To overcome this difficulty, true collagenases, i.e. those enzymes that process natively folded triple-helical collagen, must unwind the molecule locally to make the cleavage sites accessible to the enzyme. Based on high-resolution cryo–electron microscopy findings the authors demonstrate that ColH encircles a single collagen triple helix via its AD and PD domains. Via the hydrophobic AD exosites the triple helix is eventually stripped of its stabilizing hydration shell by the enzymes, and the substrate's leading and middle strand are bound and sterically distorted by binding to the two AD exosites. The displaced trailing strand is threaded via "bight" formation mediated by Trp431 into the active-site cleft. Exploiting collagen's geometry for continuous degradation, the authors propose that small-amplitude AD-PD movements, i.e. cycling between "ratcheted" and "closed" stated, allow for triplet-by-triplet translocation and its stepwise processing.
The present work is of great importance to the field and beyond, and deserves publication in Nature Communications.
Merely, the following minor issues should be dealt with in a revised version of the manuscript before prior to acceptance for publication.

Thank you for the careful and detailed review of our work and for recognising the significance of our findings, that is, the mechanism of collagen triple-helix unwinding and processive cleavage by ColH. We are grateful for the positive evaluation of our study and its contribution to the field. We carefully addressed the issues raised below and made the corresponding changes in the revised manuscript to further clarify and strengthen our work.

The numbering of the references is not chronological. Please correct.

Response: Thank you for pointing this out. The references have been renumbered and arranged in chronological order in the revised manuscript.

It would ease visual comparison with past publications in the field, if the authors would use the conventional presentation of the structure of the bacterial collagenases starting with AD on the left followed by the PD on the right etc., i.e. a horizontal presentation of the structure instead of the chosen "vertical one".
The horizontal presentation of the collagenase has the added benefit that it is conforms better with the standard orientation for (metallo-)peptidases (DOI: 10.1016/j.bbapap.2011.04.014).

Response: In preparing the draft, we carefully considered the consistency of the figure orientation with that of previous publications. However, it was not possible to clearly represent the enzyme motions in the conventional

orientation. Therefore, we deliberately chose ‘vertical views’ for this manuscript.

Just a suggestion, but the established notation for indicating the sequence of collagen-like peptides is easier on the eye, i.e. (POG)10 not POG10.

Response: We changed the notation of PPG10 and POG10 to $(PPG)_{10}$ and $(POG)_{10}$, respectively, in the revised manuscript.

Line 45: Please insert for better clarity “triple-helical” to yield “The triple-helical conformation is …”

Response: We apologise for the lack of clarity. The manuscript has been revised to address this, and ‘triple-helical’ has been inserted so that the sentence now reads “The triple-helical conformation is …” (Line 52).

Line 51: Please insert “which” as follows in “infections and [which] have been adapted”

Response: The manuscript has been revised accordingly, and ‘which’ has been inserted into the corresponding sentence (Line 58).

Line 54: Please remove “and non-fibrillar”. Eckhard et al (2011) & Fields (2013) do not claim that bacterial collagenases can digest non-fibrillar collagen; the authors only refer to fibrillar collagens I, II and III.

Response: Thank you for pointing this out. We removed ‘and non-fibrillar’ from the sentence.

Line 54: While Eckhard et al (2011) and Wang et al (2022) have provided first evidence regarding the essential role of the AD for collagen recognition and processing, Serwanja et al. (2024) demonstrated that the AD is responsible for collagen unwinding. The authors also identified residues required for collagen unwinding. Therefore, the phrase “PD-mediated cleavage may depend on prior AD-mediated unwinding” seems misleading regarding the processing of folded collagen. Please adapt.

Response: Thank you for the careful review and constructive feedback. To better reflect the current understanding in the literature, we revised the sentence as follows:

Line 61:
“PD-mediated cleavage depends on prior AD-mediated unwinding”

Line 62: The authors should give credit to previous studies that established that bacterial collagenases and MMPs employ fundamentally different unwinding mechanisms.

Response: Thank you for pointing this out. We added citations (refs. 5, 7, 8, 9, 10) in the revised manuscript (Line 69) to acknowledge that bacterial collagenases and MMPs employ fundamentally different unwinding mechanisms, as evidenced in previous studies.

Line 69: Alhayet et al (2022) did not provide structural information on the conformation of M9 collagenases. Therefore, please remove.

Response: Thank you for pointing this out. The manuscript has been revised accordingly.

Line 71: Please replace "unlay" with "unwind" or "untwist".

Response: The word 'unlay' was chosen because the three strands are unwound and fixed in three different spatial positions.

Line 80: The collagenase module (CM) is composed of the activator domain (AD) and the peptidase domain (PD) (Eckhard et al (2011)). According to Eckhard et al (2011), the part that in this manuscript is referred to as "helper domain" constitutes a PD subdomain. If the authors can provide evidence that the helper subdomain of ColH is able to fold independently of the residual PD, then it reasonable to label it as an independent domain. But otherwise please adhere to the established nomenclature and denote it as "helper subdomain". Please correct the terminology in the manuscript/supplement/figures (either by differentiating within the PD between the "catalytic subdomain" and the "helper subdomain", where appropriate in the text/figure; or by just referring to the "PD" (not PD-HD).

Response: Thank you for the detailed comment and noting the issue regarding the 'helper domain' nomenclature. We agree that, according to Eckhard et al. (2011), this region is formally considered a subdomain of the PD.
In the revised manuscript, we have adopted the established terminology and now refer to this region as the 'helper subdomain' of the PD throughout the text, figures, and supplementary information. Where relevant, we have differentiated the 'catalytic subdomain' within the PD to improve clarity.
This important suggestion has helped us ensure consistent and accurate use of structural nomenclature, thank you.

Line 85: The authors' phrasing "The composite EM map of the AD and other domains" is suboptimal, as the term "other domains" at this point implies PD, PKD1, PKD2 and CBD. Therefore, change to "The composite EM map of the CM" instead.
In particular, this misunderstanding can arise because the authors did not reveal any information regarding the map of the domains C-terminal of the CM up to this point. A suggestion would be to move the contents of this paragraph (line 100-105) after line 84, before continuing with the text in line 85.

Response: Thank you for pointing this out. We moved the contents of the paragraph and revised the sentences as shown below (Lines 89–101).

Revised (Lines 89–101):

"Focused refinement improved AD map quality, revealing a rigid structure with orientational flexibility relative to the other domains (Supplementary Fig. 1). Our cryo-EM analysis revealed a clear density for polycystic kidney disease-like domain 1 (PKD1) but not PKD2 or the collagen-binding domain (CBD), likely owing to linker flexibility that averages out their densities in the EM reconstruction.

The composite EM map of the AD and PD-PKD1 regions revealed a 'closed' CM conformation (Fig. 1d and Supplementary Figs. 1 and 2). At the junction between AD and PD, three helices (α17, α18, and α21) formed a hinge via hydrophobic interactions, whereas gate-region interactions on the opposite side reinforced the ring (Fig. 1d). PKD1 interacts with the PD in a calcium-dependent manner (Fig. 1d). A previous small-angle X-ray scattering experiment showed that calcium depletion produces an elongated apo ColH envelope[17]. Thus, the rigid PKD1 conformation may stabilise the overall structure and restrict PKD2-CBD orientation."

Can the authors comment/speculate why the CBD domain was not visible in the EM map?

Response: Thank you for this question. The CBD, as well as the PKD2 domain, was not resolved in the current EM map, likely due to their inherent flexibility relative to the CM-PKD1. Such mobility would result in weak or averaged-out density in the EM reconstruction. We added a statement (Lines 91–94) in the revised manuscript to address this point.

Revised (Line 91–94):

"Our cryo-EM analysis revealed a clear density for polycystic kidney disease-like domain 1 (PKD1) but not PKD2 or the collagen-binding domain (CBD), likely owing to linker flexibility that averages out their densities in the EM reconstruction."

Line 85: For better local clarity please modify to "At the tip of the CM, three helices (α17 from the AD, and α18 and α21 from the PD) formed …"

Response: Thank you for the suggestion. We have modified the text accordingly to read "At the junction between AD and PD, three helices (α17 from the AD, and α18 and α21 from the PD) formed …" in the revised manuscript (Lines 96–98). The hinge region is located at the junction between AD and PD; therefore, we changed the term 'tip' to 'junction.'

Fig. 1d, Inserts: Please color the helix labels differently to make them easier to find.

Response: Thank you for this suggestion. In the revised manuscript, we have coloured the helix labels in the inserts of Fig. 1d in the same way as the domain structure to improve their visibility.

Fig. 1f: The shift between the frames 0 and 9 is very difficult to discriminate, in particular on the left side of the panel.

Response: We apologise for the lack of clarity. To address this issue, we prepared an enlarged view of the gate region (Fig. 1f). This magnified representation allows the structural features and relative differences to be more clearly visualised. The corresponding figure and caption have been updated accordingly.

Line 89 - line 92:
The authors' conclusion regarding AD–PD interdomain flexibility is difficult to evaluate without comparing the RMSD values from the individual domain alignments to the RMSD from the overall (CM) alignment. Please clarify this point for better transparency and understandability.

Response: In the revised manuscript, we discuss the interdomain flexibility between the AD and PD based on the B-factors obtained from crystallographic analysis, rather than using RMSD. As shown in Supplementary Fig. 4, the B-factors gradually increase from the C- to N-terminus of the AD, suggesting domain movements mediated by the hinge region. These observations are consistent with the 3DVA of cryo-EM data.
We added a description of these results in the revised manuscript (Lines 101–106 in the main text, and in the methods section) and also added the figures (Supplementary Fig. 4).

Revised (Lines 101–106):
"We also performed X-ray crystallography of $ColH^{WT}$ CM at room temperature, which yielded a 2.70 Å structure essentially identical to the cryo-EM model, with a root-mean-square deviation of 1.9 Å for Cα atoms (Supplementary Fig. 3 and Supplementary Table 2). Notably, crystallographic B-factors increase progressively from the C-terminus to the N-terminus of the AD, suggesting hinge-mediated flexibility between the AD and PD (Supplementary Fig. 4)."

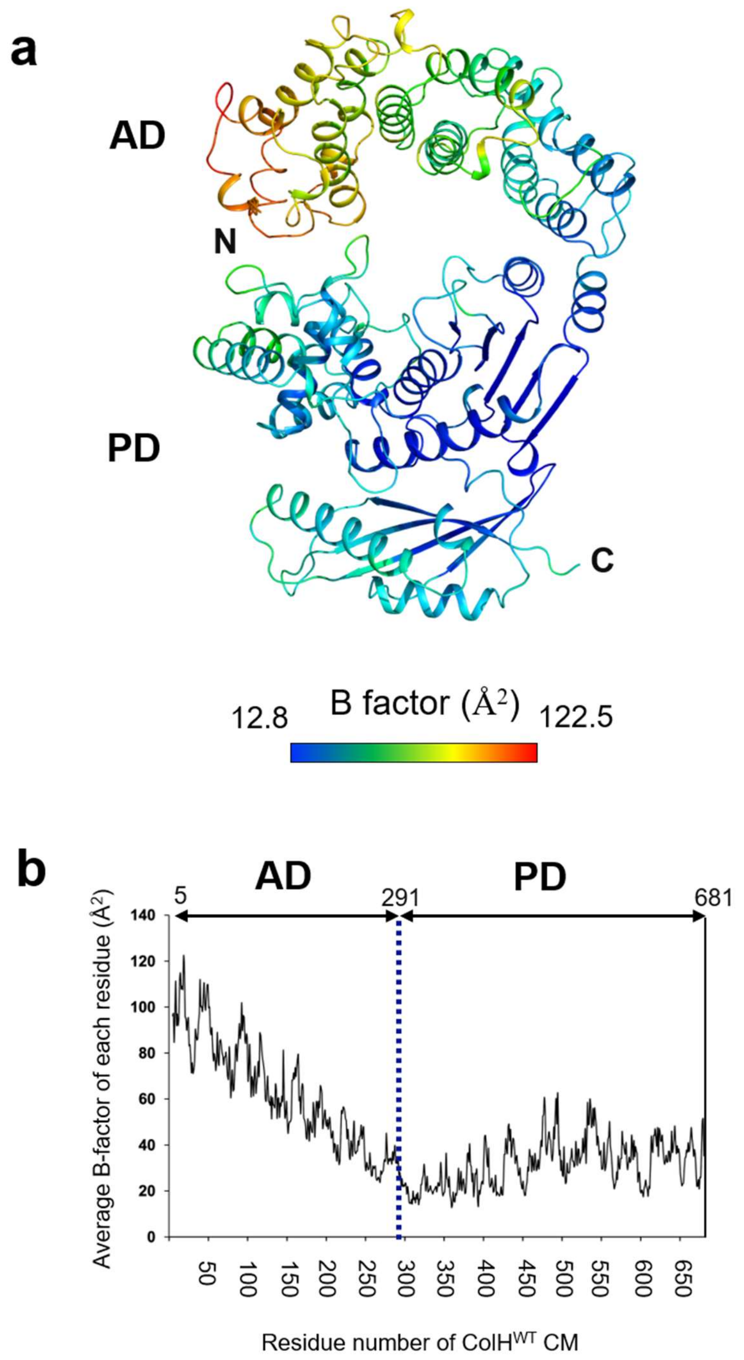

Supplementary Fig. 4. Graphical representations of ColH^{WT} CM flexibility. a Colour mapping of B-facotrs on the crystal structure of ColH^{WT} CM. The data use a colour scale from 12.75 Å^2 (blue) to 122.46 Å^2 (red). **b** Plotting of B-factors along the residue number. In the AD, B-factors increased linearly toward the N-terminus.

Line 95: Please correct to "To assess CM flexibility,"

Response: Thank you for this comment. The text has been corrected as suggested to read "To assess CM flexibility," in the revised manuscript (Line 109).

Line 99: How do these low-amplitude motions compare to the "open" conformations reported by Eckhard et al./Wang et al. & Ikeuchi et al.? Please elaborate.

Response: Thank you for this valuable comment. Our 3DVA revealed, somewhat unexpectedly, that ColH is stabilised in a closed conformation and does not adopt the wide-open structures observed in ColG and other M9 family collagenases. Although a closed conformation of ColG has not yet been reported, these structural differences may contribute to the distinct substrate specificities of these enzymes. We added sentences (Lines 315–320) explicitly addressing this point in the revised manuscript.

Revised (Lines 315–320):
"These residues may also help ColH recognise vulnerable sites in tropocollagen as it slides from the N- to C-terminus (Fig. 5c and d). Notably, the dynamic 'closed' conformation of ColH is in stark contrast to that of homologous enzymes such as ColG and some members of the M9A subfamily, which adopt more widely opened AD–PD arrangements (Supplementary Fig. 21)[5, 7, 15]. The closed-biased architecture of ColH may enhance its ability to capture rare, thermally populated local fluctuations of the collagen triple helix, thereby promoting active unwinding."

Line 128-139:
While the authors emphasize the contribution of hydrophobic interactions to substrate recognition, the role of hydrogen-bonding interactions—also clearly present—is not discussed. The hydrophobic contacts may be of greater significance. But their significance regarding the function of the AD is not corroborated by mutational studies. The proposed binding mode remains incomplete without consideration of these additional hydrogen bonds.
Specifically, hydrogen-bonding interactions stabilize the main chain of the leading strand: Arg83:NH2 forms hydrogen bonds with Gly27 and Hyp26 (2.7 Å and 2.9 Å, respectively). In addition, the position of OD1 of Hyp29 is stabilized through hydrogen bonds with Glu80 (2.8 Å) and Arg83 (3.0 Å). Both Glu80 and Arg83 appear in Figure 2e, but are not mentioned in the text. These residues are ideally positioned to stabilize the Hyp residue at the Y-position of the collagen-like sequence, whereas in ExoMiddle, the corresponding OD1 stabilization is mediated by the main-chain oxygen of Asp224.
In ExoMiddle, the CH–π interaction involving His236—observed in the $(PPG)_{10}$ and $(POG)_{10}$ model — is not described. Although the hydrogen-bonding network in this second exosite is less extensive than in ExoLead, interactions by Asp231, Arg232, and Asp224 are still present. Similarly to Glu80 and Arg83 in ExoLead, Asp231 can stabilize the hydroxyl group of Hyp26 in the Y-position. This stabilization is absent in 9LQJ, but evident in 9LYI.
Finally, it would strengthen the manuscript to mention the findings of Serwanja et al., which identified the corresponding residues to Phe37, Glu80, Arg83, Tyr87, Phe90, and Tyr184 in ExoLead in ColH as key substrate-binding residues in ColG.

Response: Thank you for this constructive comment. We agree that hydrogen-bonding interactions are also present at the binding interface. As suggested, we added descriptions of these hydrogen bonds and their potential role in substrate recognition in the revised manuscript.

Our sequence alignment and structural comparison (Supplementary Figs. 12 and 13) revealed that, although most of the residues involved in these hydrogen-bonding or CH-π interactions (such as His236 and Asn232) are not conserved across the M9 collagenase family, Arg83, which stabilises the main chain of the leading strand, is strictly conserved in both the M9A and M9B subfamilies. In addition to the conserved aromatic residues, the critical role of this arginine in collagenolytic activity has been confirmed in previous mutational studies (Serwanja et al. *Proc. Natl. Acad. Sci. U.S.A*. **121**, e2321002121, 2024; Wang et al. *Nat. Commun*. **13**, 566, 2022).

We added a discussion (Lines 165–175 in the Results and Lines 308–311 in the Discussion section) of these points in the revised manuscript and cited the relevant references (refs. 7 and 8).

Revised (Lines 165–175):

"Besides the aromatic interactions, several hydrogen-bonding interactions were observed (Fig. 2e). At Exo^{Lead}, such interactions stabilise the conformation of the leading strand: the main-chain carbonyls of Hyp26 and Gly27 form hydrogen bonds with Arg83, while the OD1 atom of Hyp29 forms hydrogen bonds with Glu80 and Arg83. Although hydrogen-bonding networks at $\text{Exo}^{\text{Middle}}$ are less extensive, an interaction between the carbonyl group of Hyp26 and Asn232 as well as OD1 of Hyp29 and carbonyl group of Asp224 stabilises the middle strand. While these interactions further strengthen peptide binding, the residues are not strictly conserved within the M9 family, with the exception of Arg83 (Supplementary Figs. 12 and 13). The importance of arginine at this position for substrate recognition in M9A and M9B collagenases has been demonstrated in mutational studies[7, 8], consistent with our structural observation that Arg83 engages and stabilises the main chain of the leading strand."

Revised (Lines 308–311):

"Recognition relies on two AD exosites, primarily Exo^{Lead}, which orient consecutive Gly–Pro–Yaa triplets via hydrophobic and CH–π interactions. Although less extensive, several hydrogen-bonding interactions, particularly those involving the conserved Arg83 at $\text{Exo}^{\text{Lead,}}$ contribute to peptide strand positioning."

Line 135: "Most Hyp residues remained solvent-exposed": While this is correct, it is, nonetheless, interesting that in ExoLead and ExoMIddle specific pockets have evolved to stabilize the hydroxyl group of Hyp residues in the Y-position. The involved residues seem to be highly conserved in the M9 family.

Response: One of our key findings is that the AD of ColH recognises the Gly–Pro–Yaa–Gly–Pro–Yaa motif. In the CoH^{MT}–$(\text{POG})_{10}$ complex structure, most Hyp residues are oriented toward the solvent or face large pockets, and some form hydrogen bonds with several residues (such as Glu80, Arg83, Asp231); however, most of these interacting residues, except Arg83, are not strictly conserved (Supplementary Figs. 12 and 13). Therefore, these

pockets, while capable of accommodating Hyp, are also likely to recognise other residues. These observations are consistent with previous proteomics data showing that clostridial collagenases exhibit selectivity for Gly–Pro–Yaa triplets (Eckhard et al., *J. Proteomics*, 2013).
In light of these considerations, we revised the relevant sections of the manuscript (Lines 176–182).

Revised (Lines 176–182):
"Most Hyp residues remained solvent-exposed and contributed little to binding, consistent with the cryo-EM data of ColH^{MT} bound to $[(Pro–Pro–Gly)_{10}]_3$ [or $(PPG)_{10}$] (Supplementary Fig. 14 and Supplementary Table 1), which showed a binding mode similar to that of the ColH^{MT}–$(POG)_{10}$ complex (Supplementary Fig. 15). These results suggest that ColH has evolved to recognise Gly–Pro–Yaa–Gly–Pro–Yaa motifs in native collagen via its hydrophobic exosites, as evidenced in previous proteomics analyses demonstrating the substrate specificity of clostridial collagenases for Gly–Pro–Yaa triplets[20]."

Line 142: "the active-site cleft, which contracted to hold the strand tightly". Please substantiate this observation (with a figure or quantify the extent of the contraction), which is – as a remark - in line with observations by Eckhard et al. (2011) NSMB and Eckhard et al. (2013) JBC.

Response: Thank you for this helpful comment. To substantiate the contraction of the active-site cleft, we added a figure illustrating the extent of cleft contraction upon substrate binding (Supplementary Fig. 16). For comparison, we also presented the structures of ColG and ColT, for which both inhibitor-bound and inhibitor-free forms have been reported. We further note that this observation is consistent with previous reports (Eckhard et al. *Nat. Struct. Mol. Biol.* **18**, 1109–1114, 2011; Eckhard et al. *J. Biol. Chem.* **288**, 20184–20194, 2013), and have cited these studies accordingly in the revised text (Lines 182–188).

Revised (Lines 183–189):
"Although the leading and middle strands followed a triple-helical axis with minor deviation, the trailing strand veered sharply, threading the three triplets at its C-terminus (Gly21–…–Gly30; P6 to P4′) into the active-site cleft (Fig. 2c, e). This distortion is critical for Zn^{2+}-mediated chelation of the scissile carbonyl. As observed in the inhibitor-bound structures of other M9B collagenases[5, 16], the active-site cleft embraced by the N- and C-terminal half-domains of the PD catalytic subdomain was contracted as much as 3 Å, holding the trailing strand tightly (Fig. 2d and Supplementary Fig. 16)."

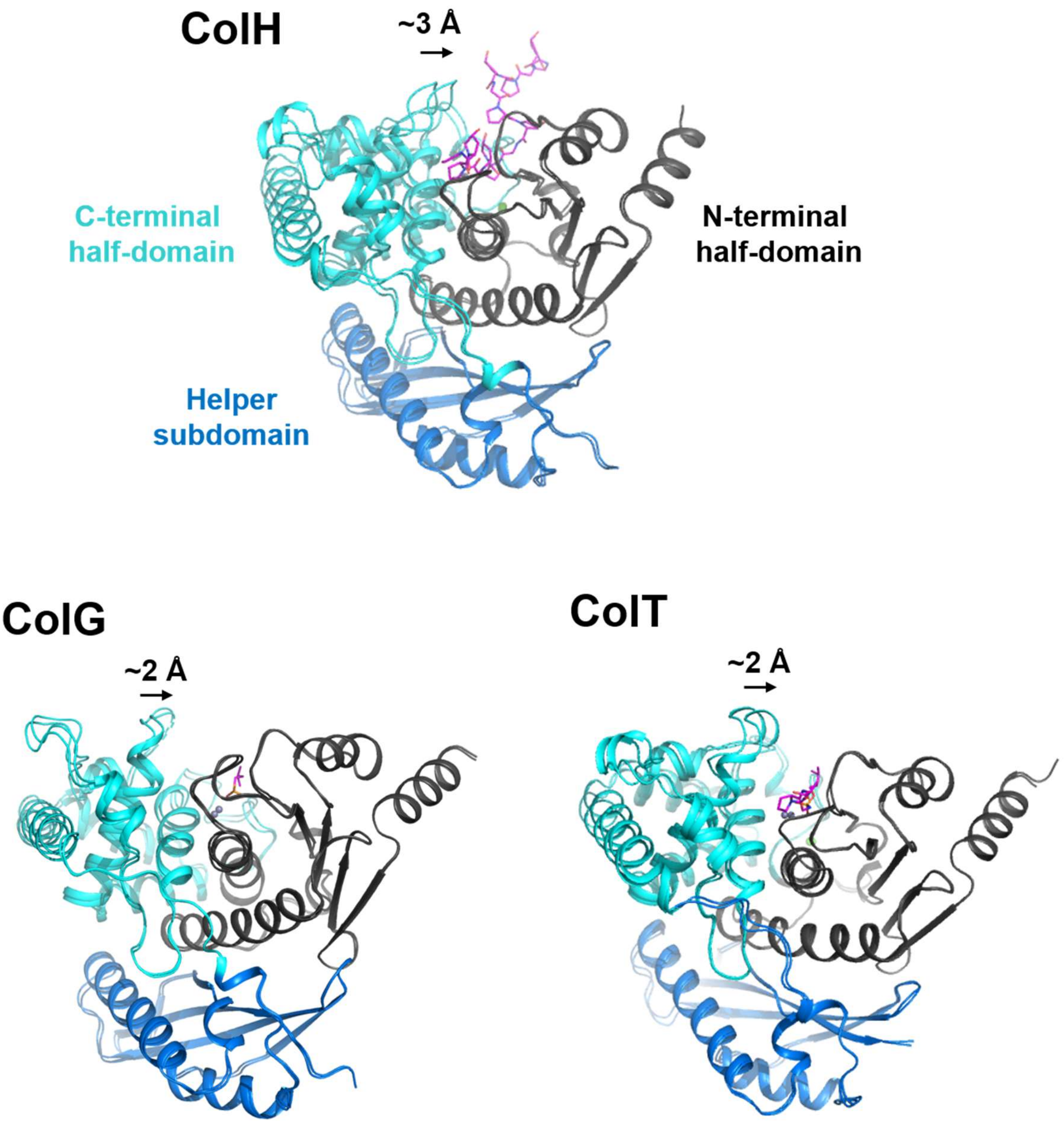

Supplementary Fig. 16. Ligand-induced contraction of PD catalytic subdomain in M9B collagenases. Superpositions of ligand-bound and ligand-free PD structures for ColH (top), ColG (bottom left), and ColT from *Clostirdium tetani* (bottom right). In each case, the superposition was performed using the N-terminal half-domain, and contraction of the C-terminal half-domain relative to the well-overlapped N-terminal half-domain is indicated by arrows. The N-terminal and C-terminal half domains are coloured grey and cyan, respectively. The helper subdomain is coloured blue.

Line 144: "with conserved Gly385 and Gly386, known as the double-glycine S1' recognition site (Ref. 3, 17)"

Response: Thank you for the suggestion. We have modified the text to read "with conserved Gly385 and Gly386 on the β4 edge strand, known as the double-glycine S1' recognition site[5, 16]" in the revised manuscript (Lines 189–190).

Fig. 1: In Fig. 2-4, the authors provide an image illustrating the quality of the EM map by showing the EM map surrounding the collagen-like peptides. Also in Fig. 1, it would be of benefit to illustrate the quality of the high-resolution EM map. In case the visualization allows it, the residue sticks in the inserts, for example, could be overlaid with the EM map.

Response: We appreciate the helpful comment. In this study, $ColH^{WT}$ shown in Fig. 1 was analysed at near-atomic resolution (2.3 Å), allowing us to clearly visualise even the water molecules around the active site. Therefore, as also suggested by another reviewer, we prepared and included an EM map focused on the active site (Supplementary Fig. 7). We believe that this more clearly demonstrates the quality of our analysis.

Fig. 2 g: The labeling of "Pz-peptidase" and "collagenase" could potentially confuse the reader. Different variants of ColH were tested against two different substrates, i.e. Pz-peptide and collagen. Therefore, I recommend using the substrate names in the legend on top of the bar graph. Please amend the figure caption stating that E416Q represents a negative control.

Response: Thank you for noting our mistakes in preparing Fig. 2g (Fig. 2f in the revised manuscript). We removed the explanations of Pz-peptidase and collagenase assays from the graph and described them in the legend. We used reaction buffers as the negative controls for the assays.

Line 147-148: The collagenase and peptidase activity of ColH were reduced by the Trp431Ala mutation. The reduction in turnover of both substrates is understandable given the important role of Trp431 in correctly positioning/orienting the substrate "bight" to enable binding of the residual C-terminal residues into the active-site cleft.

Response: We agree with the interpretation and have rephrased the relevant sentence in the revised manuscript to highlight the crucial role of Trp431 in correctly positioning and orienting the substrate 'bight,' thereby promoting proper binding of the residual C-terminal residues within the active-site cleft (Lines 221–223).

Revised (Lines 221–223):
"These results further substantiate the critical role of Trp431 in ensuring stable substrate binding and correct orientation within the active-site cleft for efficient hydrolysis."

Line 148: "Because Trp431 lies on a calcium-binding loop, these results further support the essential role of Ca2+ coordination in ColH catalysis." This statement might not be appreciated by the audience, when they are not familiar with the significance of this calcium-binding site in bacterial collagenases. In Eckhard et al. (2013) (Ref. 17) it was proposed that "[d]ue to its location between the two catalytic helices, calcium binding braces the upper and lower half-domains of the peptidase and stabilizes thereby the zinc coordination" in the active site. Thus, calcium binding, on the one hand, stabilizes the zinc in the active site, and, at the same time, the

calcium-binding loop also ensures the correct positioning of Trp431 for pre-orienting the substrate and "threading" it into the active-site cleft.

Response: Thank you for this constructive comment. In the revised manuscript, we note that, as proposed by Eckhard et al. (2013, ref. 16), calcium binding braces the upper and lower half-domains of the peptidase, thereby stabilising zinc coordination in the active site. In addition, we highlight that the calcium-binding loop also positions Trp431, facilitating pre-orientation of the substrate and its proper threading into the active-site cleft (Lines 195–200).

Revised (Line 195–200):
"Notably, Trp431 lies on a calcium-binding loop located between the N- and C-terminal half-domains, where a conserved glutamate side-chain (Glu390 in ColH) anchors the edge strand and calcium, thereby bracing the two half-domains (Fig. 2g). Consequently, calcium binding not only stabilises zinc coordination in the active site but also ensures the proper positioning of Trp431. These observations support the essential role of Ca^{2+} coordination in ColH catalysis[16]."

Line 150-153:
Quantitative comparisons would help to clarify the magnitude of the observed effects in the alanine scanning. From the results shown in Fig. 2g, it can be concluded that Trp431 plays a vital role in substrate turnover for unfolded and triple-helical substrates. In case of Tyr235Ala, the mutation had a noticeable effect (~30% decrease in collagenolytic activity), whereas when all three aromatic residues of ExoMiddle were mutated an overall decrease by ~65% was observed. In comparison, the Phe90Ala mutation in ExoLead alone reduced the activity by approx. 60%. Alanine mutation of the three residual aromatic residues of this exosite led to a further decrease by 30%. This seems to suggest that the presented "three pivots" differ in their functional significance. Please elaborate more on this point. While the significance of Trp431 and Phe90 become apparent from the presented data, it is difficult to ascertain whether Tyr235 is the most major determinant in ExoMiddle, when the single alanine mutations of the other residues of this exosite are not presented. I would recommend to add more mutational data regarding ExoMiddle.

Response: Thank you for this important comment. We agree that quantitative comparisons provide insight into the relative functional contributions of the aromatic residues in the Exo^{Lead} and Exo^{Middle} sites.

As noted, the qualitative comparison indicated differences in the contributions of the three aromatic residues, with Tyr235 in Exo^{Middle} having a weaker effect than Phe90 in Exo^{Lead}. This trend was also observed in the four-residue Exo^{Lead} mutant (F37A/Y87A/F90A/Y184A) and three-residue Exo^{Middle} mutant (F176A/W228A/Y235A), suggesting that the overall contribution of Exo^{Middle} is smaller than that of Exo^{Lead}. This is also consistent with our sequence and structural comparisons showing that the residues in Exo^{Middle} are not conserved, reflecting the variation in substrate recognition at this exosite (Supplementary Figs. 12 and 13).

Given the relatively minor contribution of Exo^{Middle} residues to collagenase activity, we did not perform further mutational experiments; however, the manuscript has been revised to address these points (Lines 147–164 and 200–209).

Revised (Lines 147–164):

"Partial unfolding at the C-terminus allows the leading and middle strands of (POG)$_{10}$ to bind to two AD exosites (Exo^{Lead} and Exo^{Middle}; Fig. 2b, e). Both sites engage two contiguous triplets (Pro25–…–Gly30) via hydrophobic clefts. Proline residues at X positions (Pro25 and Pro28) form contacts with aromatic residues—Phe37, Tyr87, Phe90, and Tyr184 at Exo^{Lead}; Phe176, Trp228, and Tyr235 at Exo^{Middle}—through hydrophobic and CH–π interactions (Fig. 2e). Alanine substitution of these residues significantly reduced type I collagen hydrolysis but not 4-phenylazobenzyloxycarbonyl-Pro-Leu-Gly-Pro-D-Arg (Pz-peptide) hydrolysis, highlighting their role in triple-helix recognition and unwinding (Fig. 2f). Mutations in the Exo^{Lead} residues caused a substantially greater reduction in collagenolytic activity than those in Exo^{Middle} (~90% versus ~60%), suggesting that Exo^{Lead} plays a more critical role in collagen recognition and unwinding. Sequence and structural comparisons with M9A and M9B collagenases showed that Exo^{Lead} residues are strictly or highly conserved across these enzymes (Supplementary Figs. 12 and 13). Consistently, their importance for collagenolytic activity in both subfamilies has been confirmed in previous mutational studies[7, 8]. In contrast, the aromatic residues comprising Exo^{Middle} in ColH are intriguingly not conserved within the M9 family. In the M9B subfamily, although Y235 and W228 are conserved, F176 is not, whereas in M9A, only the residue equivalent to W228 is conserved. This relatively low level of conservation may underlie the variability in substrate binding at this exosite."

Revised (Lines 201–210):

"When viewed from the front face of ColH^{MT}, three aromatic residues, Phe90, Tyr235, and Trp431, engaged the interacting triplets as aromatic pivots marking the transition from a helical to unwound structure (Fig. 2h). These residues may enhance local bending to promote strand unwinding or favours partially unfolded triple-helical structures, facilitating recognition of the frayed C-terminal region of (POG)$_{10}$[21]. Substitution of Phe90 greatly reduced collagenolytic activity, whereas Tyr235 mutation led to a more modest decrease (~60% versus ~20%; Fig. 2f). Although Tyr235 is not conserved in the M9A family (Supplementary Fig. 12), a phenylalanine residue that may serve a similar function is positioned nearby (Supplementary Fig. 13). This suggests that an aromatic residue at this position supports the binding and stabilisation of the unwound collagen triple helix."

Line 154: I would recommend to rephrase the second part of the sentence for better clarity [regarding "or favours partially unfolded. POG10"].

Response: Thank you for this note. The corresponding section has been rephrased as follows:

Revised (Lines 203–205):

"These residues may enhance local bending to promote strand unwinding or favours partially unfolded triple-

helical structures, facilitating recognition of the frayed C-terminal region of $(POG)_{10}$[21]."

Line 164: "they may [have] facilitate[d] additional ColH recruitment"

Response: Thank you for the suggestion. We have made the corrections in the revised manuscript (Line 239).

Line 178:

The authors state that "ExoLead-driven unwinding of POG10 was restricted because ColHMT-C bound at the C-terminus blocks further unravelling by ColHMT-N." The observation that ColHMT-N was unable to unwind the bound triple-helical collagen is interesting. The authors conclude that ColHMT-N could not unwind the triple helix due to the presence of ColHMT-C, implying that the C-terminal unwinding event precludes N-terminal unwinding. Can the authors clarify whether this interpretation is consistent with the conclusions drawn for the complex with (POG)12? At first glance, the two conclusions/statements appear to be contradictory.

Staying with the statement "ColHMT-C bound at the C-terminus blocks further unravelling by ColHMT-N": The authors seem to suggest that C-terminal unwinding induced a strain in the triple-helix which precluded a second unwinding event in close proximity. Or do I misinterpret the author's statement? If yes, please provide an alternative explanation for the blockage? If no, is there any indication in the structural data to support the "strain hypothesis"?
There could be an alternative interpretation of the observed absence of unwinding in case of ColHMT-N, namely that due to the absence of the middle and trailing strand at the recognition site of the leading strand (due to the mis-stagger) the local structural perturbation induced by ExoLead on the leading strand could not be further sterically propagated to the middle and trailing strand (which would then have been facilitated and stabilized by binding to ExoMiddle and the active-site cleft), thus preventing local unwinding and thus resulting in an unproductive binding event. Would this interpretation be consistent with the findings regarding the complex with (POG)12?

Response: We apologise for the lack of clarity. We propose that this inhibitory effect is not due to the unwinding of $ColH^{MT}$-C, but rather that $ColH^{MT}$-C acts as a steric impediment in the 2:1 $ColH^{MT}$–$(POG)_{10}$ complex, thereby hindering further unwinding by N-terminally bound ColH-N.

To clarify this point, we added Supplementary Fig. 19 illustrating the apparent steric hindrance between the PKD1 domain of $ColH^{MT}$-N and the CM of $ColH^{MT}$-C in the 2:1 $ColH^{MT}$-$(POG)_{10}$ complex.

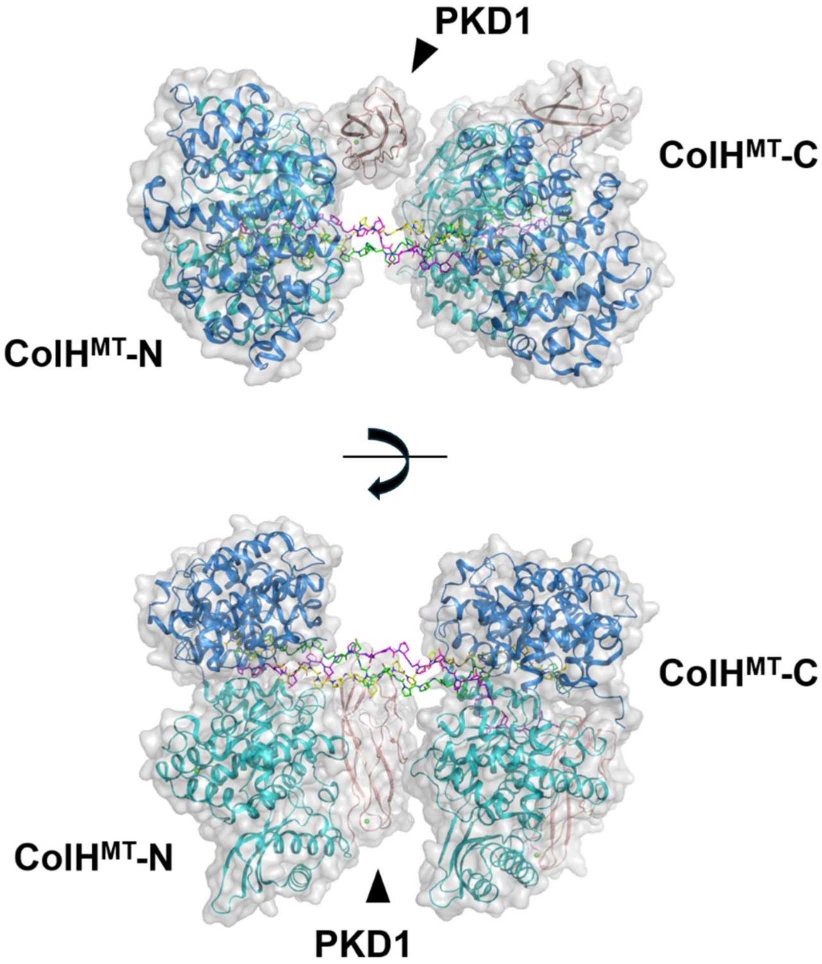

Supplementary Fig. 19. Steric hindrance between PKD1 of ColH^{MT}-N and CM of ColH^{MT}-C in the 2:1 ColH^{MT}–(POG)$_{10}$ complex. In the complex, ColH^{MT} molecules are depicted as surface representations. The PKD1 of ColH^{MT}-N, which is likely involved in the steric hindrance, is indicated by an arrowhead.

As noted, we observed that ColH^{MT}-N binds at the N-terminus and induces local structural changes (Fig. 2), particularly in the mis-staggered leading strand via Exo^{Lead}; however, these changes cannot propagate further, as they are insufficient to allow the middle and trailing strands to engage stably with Exo^{Middle} and the active site, respectively, due to the aforementioned steric hindrance. We have included a sentence (Lines 253–258) in the revised manuscript to clarify this situation. Note that this steric hindrance would not occur for longer substrates such as (POG)$_{12}$.

Revised (Lines 253–258):

"Nevertheless, Exo^{Lead}-driven unwinding of (POG)$_{10}$ in the complex may be restricted by steric hindrance between the PKD1 of ColH^{MT}-N and CM of ColH^{MT}-C already bound at the C-terminus (Fig. 3a and Supplementary Fig. 19). This steric hindrance could impede N-terminal progression of ColH^{MT}-N and thereby limit further N-terminal unravelling of (POG)$_{10}$, potentially reducing stable engagement of the partially unwound middle and trailing strands by Exo^{Middle} and the active site, respectively."

Lines 200-201: Concerning the observed complete unfolding of $(POG)_{12}$ in complex with ColHMT, the authors should clarify why this observation supports the conclusion of N-to-C directional movement along the triple helix. Additionally, please explain why a C-to-N directional movement would be inconsistent with the data, as this is not immediately clear from the text. Please also discuss why not a similar behavior (i.e. complete unwinding of the triple helix upstream of the C-terminally bound ColH) was observed regarding the complex with (POG)10.

Response: We appreciate the comment regarding the $ColH^{MT}$–$(POG)_{12}$ complex. Assuming that regions that are easier to unwind are preferentially recognised by ColH, we initially hypothesised that the second $ColH^{MT}$ molecule would unwind and stabilise the three triplets at the N-terminus of $(POG)_{12}$, resulting in a 2:1 $ColH^{MT}$–$(POG)_{12}$ complex such as a 2:1 $ColH^{MT}$-$(POG)_{10}$ complex. However, our experimental observations did not support this scenario.

As described in the manuscript, even if $ColH^{MT}$ were to bind to both termini of $(POG)_{12}$ and unwind three triplets at each end, the central region (about six triplets) would still retain the triple-helical structure. Therefore, a pentameric dumbbell-like structure would be expected (Fig. 4f, middle). However, such a structure was not observed. This can be reasonably explained by $ColH^{MT}$ bound at the N-terminus migrating to the C-terminus while progressively unwinding the triple helix (Fig. 4f, right).

Since $ColH^{MT}$ can bind to any site on $(POG)_{12}$ and translocate bidirectionally along the triple helix, as noted by the reviewer, we cannot exclude an alternative possibility in which a second $ColH^{MT}$ molecule binds immediately adjacent to $ColH^{MT}$-C and induces global unwinding of $(POG)_{12}$ by migrating from the C- to N-terminus. To address this issue, we have revised the Results section to explicitly clarify these possibilities (Lines 282–283).

Revised (Lines 282–283):
"Here, we cannot exclude that a second $ColH^{MT}$ binds adjacent to $ColH^{MT}$-C and subsequently migrates toward the N-terminus, inducing global unwinding of $(POG)_{12}$."

Line 203: "shows [a] dynamic "closed" state]" Please correct.

Response: Thank you for this note. The text has been corrected to read "shows a dynamic 'closed' state" in the revised manuscript.

Line 204/Line 206: The authors correctly note that their findings "support" (line 104) the conclusion that ColH can adopt a ratcheted state only when bound to a partially unwound substrate. In light of this, the subsequent statement that "[t]hese results demonstrate that ColH exploits …" should be moderated, as the data support the claim but do not entirely demonstrate it.

Response: Thank you for raising this point. Accordingly, we have revised the wording in Lines 204–206 of the original manuscript by replacing 'demonstrate' with 'suggest,' and have revised the sentence to more accurately reflect the scope of our findings.

Line 207: Please replace "unlaying" with "unwinding".

Response: The word 'unlaying' was chosen because the three strands are unwound and fixed in three different spatial positions.

Line 211: The manuscript mentions "previous studies"; please include the appropriate citations to clarify which studies are being referenced.

Response: We acknowledge the lack of clarity. We have now added the appropriate citations to identify the studies being referenced.

Line 212: Please change to plural, i.e. "how bacterial collagenases recognize the collagen triple helix and exploit its geometry for processive stand-specific cleavage.

Response: We appreciate the comment. We have revised the text to use the appropriate form.

Discussion: Please elaborate on how the presented results fit with the observed "open" conformation of bacterial collagenases reported by Eckhard et al (Ref. 3), Wang et al. (Ref.10), Ikeuchi et al. (Ref.15), designated in this manuscript as "wide-open" conformation. It would be very interesting to see a figure in which the dimensions of the different opening or closing states, respectively, are compared.

Response: Thank you for this valuable comment. In the revised manuscript, we included Supplementary Fig. 21 and text comparing the dimensions of the different opening and closing states, showing the closed conformation of ColH relative to the previously reported 'wide-open' conformations of other bacterial collagenases (refs. 5, 7, 15 in the revised manuscript). This comparison highlights how the distinct open–closed dynamics may relate to differences in substrate specificity.

Revised (Lines 315–320):
"These residues may also help ColH recognise vulnerable sites in tropocollagen as it slides from the N- to C-terminus (Fig. 5c and d). Notably, the dynamic 'closed' conformation of ColH is in stark contrast to that of homologous enzymes such as ColG and some members of the M9A subfamily, which adopt more widely opened AD–PD arrangements (Supplementary Fig. 21)[5, 7, 15]. The closed-biased architecture of ColH may enhance its ability to capture rare, thermally populated local fluctuations of the collagen triple helix, thereby promoting active unwinding."

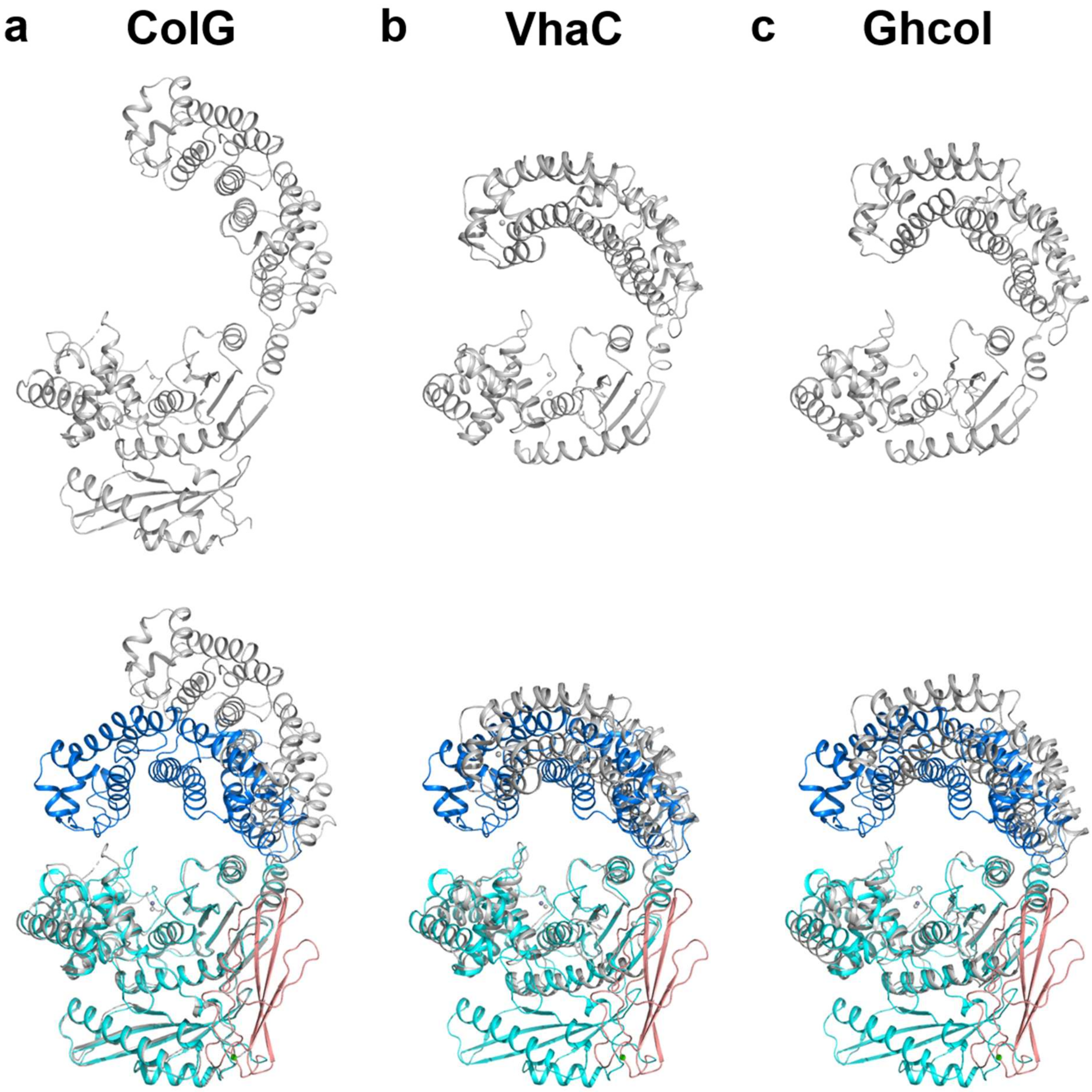

Supplementary Fig. 21. Structural comparison of the CM of M9 family collagenases. a Overall structure of M9B collagenase ColG from *H. histolytica* (PDB ID: 2Y50). **b** Overall structure of M9A collagenase VhaC from *V. harveyi* (PDB ID: 7ESI). **c** Overall structure of M9A collagenase Ghcol from *G. hollisae* (PDB ID: 7WSS). In each case, the superposition with ColH^{WT} from *H. histolytica* (this study) was performed using the PD catalytic subdomain, shown in the bottom panel.

Line 218: The ColHMT-N-POG10 complex represents an "unproductive" substrate binding mode (resulting probably from the mis-staggered triple helix at this location). While this is unfortunate, this complex nonetheless illustrates how ColH could utilize the captured, but unwound substrate as "rail tracks" for its one-dimensional diffusion, reminiscent of DNA-binding clamps. Please change the text to reflect this conclusion by the authors.

Response: Thank you for this valuable comment. We have revised the text to clarify that, although the $ColH^{MT}$-N-$(POG)_{10}$ complex represents an 'unproductive' substrate-binding mode (likely due to the mis-staggered triple helix at this location and/or the steric hindrance by $ColH^{MT}$-C), it nonetheless illustrates how ColH could use the captured, but unwound, substrate as 'rail tracks' for one-dimensional diffusion, analogous to DNA-binding clamps.

Revised (Lines 301–306):
"As shown in the $ColH^{MT}$–N–$(POG)_{10}$ complex, which represents an unproductive binding mode likely arising from a mis-staggered triple helix and/or steric hindrance by $ColH^{MT}$-C, the CM appears to use the captured substrate as 'rail tracks' for one-dimensional diffusion. This directional movement may enable the enzyme to search for vulnerable regions, reminiscent of the behaviour of DNA-binding clamps and endonucleases (Figs. 3 and 5c, d)[25, 26]."

Line 235-236: I would recommend the use of [cleavage] instead of [release]: "This captured bight is critical for translocation and reloading of the substrate, enabling [cleavage] of a Gly-X-Y tripeptide".

Response: Thank you for this helpful suggestion. We have replaced 'release' with 'next-step cleavage.'

Line 238: "underscoring [the] mechanistic divergence between bacterial and mammalian enzymes." Please correct.

Response: Thank you for this note. We have corrected the sentence to read "underscoring the mechanistic divergence between bacterial and mammalian enzymes."

Lines 240-248: Please clarify that the authors present and propose here a new refined model of collagen degradation by bacterial collagenases.

Response: Thank you for this constructive comment. We have rewritten the text to explicitly present the refined model of collagen degradation by bacterial collagenases.

Revised (Lines 332–343):
"The cleavage mode also differs between bacterial collagenases and MMPs. MMPs act exclusively as endopeptidases, whereas ColH advances toward the N-terminus by releasing trailing-strand tripeptides through carboxytripeptidase activity[13]. Here, we present a refined model in which, after each cleavage event, the three-residue bight passively dissociates from the active-site cleft due to reduced post-cleavage affinity. The next triplet then moves into the S1′–S3′ subsites as the CM domains realign (Fig. 5g, h). This requires transient loosening of AD–PD interactions, allowing the AD to twist back by ~10 Å (one triplet) (Fig. 2d). A conserved 'wall motif' in the distal S3′ pocket, unique to M9B collagenases, enforces this stepwise C-to-N progression.

Intermittent AD engagement with the leading and middle strands further ensures triplet-spaced translocation. Through cycles of ‘closed’ and ‘ratcheted’ states, ColH trims one trailing-strand triplet at a time, consistent with the C-to-N directionality previously observed for ColG at the fibril level[14].”

Line 242: The term “unloads” implies active intervention by the collagenase. Was this the intended meaning? It seems more likely that the cleaved products dissociate due to reduced affinity for the active-site cleft.

Response: We appreciate the helpful comment. We revised the text to indicate that the cleaved products likely dissociate due to reduced affinity for the active-site cleft, rather than being actively ‘unloaded’ by the enzyme.

Revised (Lines 334–337):
“Here, we present a refined model in which, after each cleavage event, the three-residue bight passively dissociates from the active-site cleft due to reduced post-cleavage affinity. The next triplet then moves into the S1′–S3′ subsites as the CM domains realign (Fig. 5g, h).”

Line 246: I suggest including a reference to Watanabe-Nakayama et al. (2016), who demonstrated catalytically productive unidirectional movement of ColG on collagen at the fibril level from C-to-N terminus, as this would provide useful context for the reader.

Response: Thank you for this suggestion. We have added a reference for Watanabe-Nakayama et al. (2016) in Line 343 to provide context regarding the catalytically productive unidirectional movement of ColG on collagen from the C- to N-terminus at the fibril level.

Where is Reference 26 cited?

Response: Thank you for noting the missing citation. We have removed ref. 26 in the revised manuscript.

Line 250 (typo->350): “was added [to remove the GST tag]”

Response: We revised the manuscript ss suggested.

Line 361: Please provide buffer/elution details regarding the ion-exchange chromatography.

Response: Following your guidance, we made the following revisions:

Revised (Lines 404–408):
“After removal of the GST tag by affinity chromatography, proteins were purified by ion-exchange chromatography with Q-Sepharose Fast Flow beads (Cytiva) equilibrated with 50 mM Tris-HCl (pH 7.5)

containing 1 mM $CaCl_2$. The flow-through fraction containing $ColH^{WT}$ CM was further purified by size-exclusion chromatography as described above."

Line 368: Please provide buffer details for the reconstitution of the peptide solutions.

Response: Thank you for pointing this out. The peptides were reconstituted in the same cryo-EM buffer used throughout this study (50 mM Tris-HCl, pH 7.5; 100 mM NaCl; 1 mM $CaCl_2$). This information has been added to the Methods section (Lines 415-416) to improve clarity.

Line 672: It would be improve clarity if the authors elaborated why the (hypothesized) N-terminally bound ColH molecule could not be observed by cryo-EM, while the C-terminally bound ColH was.

Response: As shown in Fig. 4, in the case of $(POG)_{12}$, stable binding of the second $ColH^{MT}$ molecule ($ColH^{MT}$-N) allows unwinding to proceed in the N-to-C direction (although C-to-N movement may also be possible), thereby promoting overall unwinding. Once fully unwound, the two molecules ($ColH^{MT}$-N and $ColH^{MT}$-C) are difficult to analyse collectively owing to their orientational flexibility or the possible dissociation of one molecule from the unwound peptides. Since $ColH^{MT}$-N and $ColH^{MT}$-C are expected to recognise the unwound peptide segments similarly, only an averaged structure is obtainable.

We have revised the text (Lines 283–285) to incorporate this point.

Revised (Lines 283–285):
"Once fully unwound, the two molecules are difficult to analyse collectively owing to their orientational flexibility or possible dissociation of one molecule from the unwound peptides."

Line 507: The method used to assess collagenolytic activity may not be perfectly able to distinguish between gelatinolytic and collagenolytic activity, as the assay was performed at 37°C, a temperature at which singly-accessible collagen molecules on the surface of the fibril will already start to denature (doi: 10.1073/pnas.032307099).

Response: Thank you for this valuable comment. It is well known that triple-helical tropocollagen is hydrolysed by true collagenases, but that denatured collagen (gelatin) is hydrolysed by a wide variety of proteases. Wang et al. (*Nat. Commun*. **13**, 566, 2022) reported that trypsin (negative control) showed negligible activity against insoluble type I collagen fibres, suggesting that the amount of gelatin on the surface of collagen fibres does not significantly affect the collagenase assay. Apart from this, the amount of soluble product in the buffer control was ~1.5% of that in the positive control ($ColH^{WT}$), showing minimal spontaneous release of the water-soluble substrate.

Regarding the reproducibility of the data, although being only a minor issue, I would like to remark that I could numerically not reproduce the RMSD deviations for bond lengths and bond angles for all reported EM structures using PHENIX. Please check.

Response: Thank you for carefully checking the stereochemical parameters. We think that the small discrepancies in bond-length and bond-angle RMSD values likely arise from differences in PHENIX versions and refinement settings. In our study, model refinement was performed using PHENIX version 1.20.1-4487 with default geometry restraints optimised for cryo-EM structures.

Details of the software versions used in the structural analysis are described in the Nature Portfolio Reporting Summary and are also available on the PDB website as part of the registration information for each X-ray or cryo-EM structure.

Regarding the reported crystal structure for ColH CM, the Rwork/Rfree ratio (0.224/0.283) indicates overfitting of the density. Please revise your model.

Response: As noted, the R_{free} value differed by ~6% from the R_{work} value in the original model, raising concerns about overfitting. We therefore re-refined the structure, with particular attention to the placement of water molecules and the AD domain, which exhibited elevated B factors. Consequently, we obtained an improved model with R_{work} = 0.231 and R_{free} = 0.271, which has been deposited in the Protein Data Bank under accession code 9CME. Accordingly, we have revised Supplementary Table 2.

Line 709:"as described in Extended Data Fig. 6, and Supplementary Videos 1 and 2", these references appear to be mis-placed.

Response: Thank you for this note. We have corrected the legend for Supplementary Fig. 1.

Line 710: Please correct to "activator domain" (not accessory domain).

Response: Thank you for noting this mistake. We have corrected 'accessory domain' to 'activator domain' in the legend for Supplementary Fig. 1.

Line 711: Remove full stop after ColHWT.

Response: Thank you for this note. We have removed the full stop after 'ColH^{WT}' in the legend for Supplementary Fig. 1.

Line 741: "as described in Extended Data Fig. 5 and [illustrated] in Supplementary Video 3"

Response: Thank you for your careful review. This part of the sentence has been revised to read "as described in Supplementary Fig. 5 and illustrated in Supplementary Video 3."

Line820: Please correct to "PPG10".

Response: Thank you for pointing this out. '$(PPG)_{10}$' has been used throughout the revised manuscript.

Extended Data Fig. 1 & Extended Data Fig.4: Please provide also the intersection with FSC = 0.143 (map-to-map) and the corresponding resolution.

Response: To address this comment, the composite map and its associated composite half maps generated by *phenix.combine_focused_maps* were imported into cryoSPARC, and map-to-map FSC curves were computed using the Validation (FSC) job.

In the revised manuscript, the resulting curves and corresponding resolution values conforming with the FSC = 0.143 criterion have been added to Supplementary Figs 1, 8, 9, and 20.

Point-by-point responses for reviewer's comments

Reviewer #1 (Remarks to the Author)

I appreciate the authors efforts to address all critiques raised in the first round of review. From this reviewers perspective the manuscript and models have been significantly improved. I support publication of this manuscript.

We are grateful for your careful assessment of our manuscript and for the substantial time and effort you have invested in the review process. We greatly appreciate your acknowledgment of the improvements made in the revised manuscript, and are encouraged by your support for publication.

Reviewer #2 (Remarks to the Author)

The authors have made good efforts to address the reviewer comments and the manuscript has significantly improved. Some minor suggestions for the revised manuscript are as follows:

We thank the reviewer for the positive and encouraging assessment of our revised manuscript and are also grateful for the additional minor suggestions, which have helped us further refine the quality of the manuscript. Our detailed responses to each comment are provided below.

1. The author did not include an introduction to the collagenases of the two subfamilies (M9A and M9B) of the M9 family in the Introduction, which made the analysis and discussion of M9A and M9B collagenases in the Results and Discussion sections appear somewhat abrupt. Therefore, I suggest adding an introduction to M9A and M9B collagenases between lines 55 and 63, such as domain composition, characteristics of cleavage sites, structural features, etc.

Response: We thank the reviewer for this helpful suggestion. In response, we have revised the Introduction (lines 55–69) to include a brief contextual overview of the M9 family and its subdivision into the M9A and M9B subfamilies. This addition clarifies their placement within the broader protease classification framework and provides smoother continuity with the analysis presented in the Results and Discussion. Detailed descriptions of domain composition, cleavage characteristics, and structural features remain available in the cited literature.

Revised (Lines 55–69):
"To degrade collagen-based structures, bacteria and metazoa produce collagenases, i.e. zinc-dependent metallopeptidases belonging to clan MA in the MEROPS database[4]. Bacterial collagenases belong to the M9 family (gluzincins) based on the sequence similarity[5, 6]. M9A collagenases are produced mainly by marine bacteria such as species of *Vibrio* and *Grimontia*, whereas M9B collagenases are produced by terrestrial bacteria such as species of

Clostridium and *Bacillus*. *Hathewaya histolytica* (formerly *Clostridium histolyticum*) produces two classes of M9B collagenases (ColG and ColH) [7-9] that act as virulence factors in soft-tissue infections, which have also been adapted for clinical applications, including wound debridement, fibrosis treatment, and islet isolation[10]. Bacterial collagenases contain multiple domains, including an activator domain (AD) and thermolysin-like peptidase domain (PD), which cooperate to unwind and cleave fibrillar collagens[11]. PD-mediated cleavage depends on prior AD-mediated unwinding[12, 13]. However, how the enzyme unwinds the triple helix and maintains the unwound state while engaging all three strands remains unclear.

Mammalian matrix metalloproteinases [MMPs, M10 family (metzincins)] have been widely studied and play a tightly regulated role in ECM remodelling."

Added references:

[4]Rawlings, N. D. et al. The MEROPS database of proteolytic enzymes, their substrates and inhibitors in 2017 and a comparison with peptidases in the PANTHER database. *Nucleic Acids Res.* **46**, D624-D632 (2018).

[5]Zhang, Y. Z., Ran, L. Y., Li, C. Y. & Chen, X.L. Diversity, structures, and collagen-degrading mechanisms of bacterial collagenolytic proteases. Appl. Environ. Microbiol. **81**, 6098-6107 (2015).

[6]Duarte, A. S., Correia, A. & Esteves, A. C. Bacterial collagenases - A review. *Crit. Rev. Microbiol.* **42**, 106-126 (2016).

[7]Mookhtiar, K. A. & Van Wart, H. E. *Clostridium histolyticum* collagenases: a new look at some old enzymes. *Matrix Suppl.* **1**, 116-126 (1992).

[8]Yoshihara, K., Matsushita, O., Minami, J. & Okabe, A. Cloning and nucleotide sequence analysis of the *colH* gene from *Clostridium histolyticum* encoding a collagenase and a gelatinase. *J. Bacteriol.* **176**, 6489-6496 (1994).

[9]Matsushita, O. et al. Gene duplication and multiplicity of collagenases in *Clostridium histolyticum*. *J. Bacteriol.* **181**, 923-933 (1999).

2. L70-L72: The author compares the differences in cleavage characteristics of collagen peptide chain between M9 bacterial collagenases and M10 mammalian matrix metalloproteinases here. However, merely citing the description of M9B collagenase is insufficient (ref 13), as M9A collagenases also exhibit cleavage site specificity for Y-Gly bonds in collagen peptide chains releasing collagen tripeptide Gly–X–Y (J Agric Food Chem. 2025 73:22079-22088; Nat Commun. 2022 13:566; Appl Environ Microbiol. 2022 88:e0167721). There is also a similar deficiency in lines 181 to 182.

Response: We thank the reviewer for this helpful comment. For M9A (Vibrio) collagenases, carboxytripeptidase activity has been inferred from collagen fibril hydrolysis experiments showing early accumulation of tripeptide products (Ikeuchi et al., *J. Biol. Chem.* 2022). Furthermore, the Yaa–Gly bond specificity has been shown by structural and high-resolution LC–MS studies (Wang et al., *Nat. Commun.*, 2022; Zhao et al., *J. Agric. Food Chem.* 2025; Wang et al., *Appl. Environ. Microbiol.*, 2022). We thank the reviewer for drawing our attention to these important studies. We have revised the manuscript accordingly and have added citations to the above-mentioned references (Lines 75–

80).

Revised (Lines 75-80):

"Unlike MMPs, which act exclusively as endopeptidases, bacterial collagenases exhibit both endopeptidase and carboxytripeptidase activities, preferentially cleaving Y–Gly bonds[12, 19-22]. The carboxytripeptidase processively trims Gly–X–Y triplets from the initial site of engagement towards the N-terminus. High-speed atomic force microscopy has demonstrated catalysis-coupled movement of a clostridial collagenase in the N-terminal direction[23], yet how strand engagement supports this processive advancement remains unclear."

Added references:

[20]Wang, Y. et al. Mechanistic insight into the fragmentation of type I collagen fibers into peptides and amino acids by a *Vibrio* collagenase. *Appl. Environ. Microbiol.* **88**, e0167721 (2022).

[21]Ikeuchi, T. et al. Crystal structure of *Grimontia hollisae* collagenase provides insights into its novel substrate specificity toward collagen. *J. Biol. Chem.* **298**, 102109 (2022).

[22]Zhao, W. X. et al. Mechanistic insight into the cleavage site specificity of *Vibrio* collagenase VhaC to the Y-G bonds in collagen. *J. Agric. Food Chem.* **73**, 22079-22088 (2025).

3. I suggest adding orange and purple color blocks in the blank area of Fig. 2f to represent the substrates Pz-peptide and collagen, respectively, so that readers can quickly grasp the activity information of the wild-type protein and its mutants toward the two substrates. This does not seem to contradict the suggestion from Reviewer 3.

Response: We thank the reviewer for his/her helpful suggestion. We have placed orange and purple color blocks at the top of blank space of Fig. 2f, while leaving the relevant caption unchanged as previously advised by Reviewer 3. Additionally, in response to the editor's requests, we have added blue dots representing the mean values of each triplicate to the figure and have included an explanation in the figure caption.

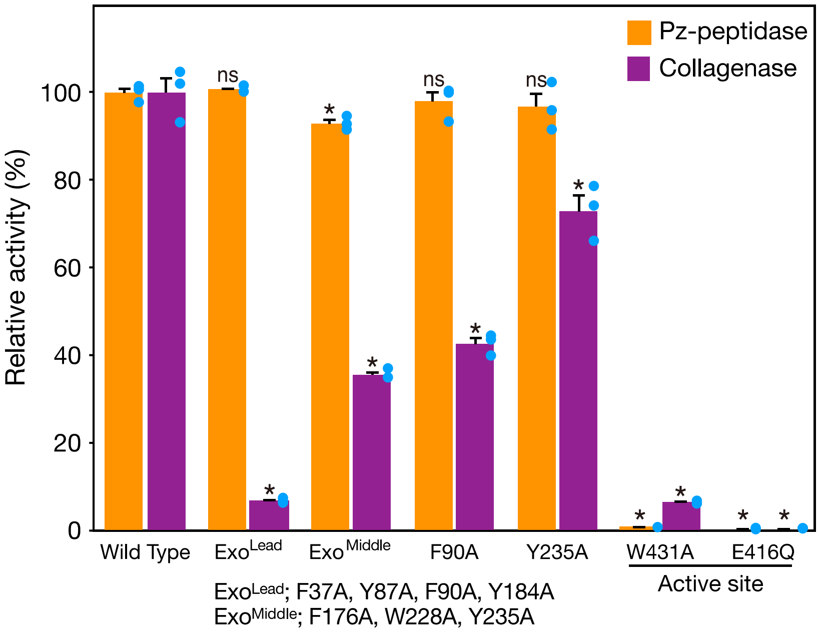

Fig. 2 ColH binds to unwound C-terminus of $(POG)_{10}$. f Relative Pz-peptidase (orange) and collagenase (purple) activities of wild-type and mutant ColH. All assays were performed in triplicate across three independent experiments. Data are presented as mean values ± SEM. Blue dots represent the mean values of each triplicate. The statistical differences between wild-type and mutant ColH were examined by a two-tailed *t*-test. The exact *p* values for Pz-peptidase and collagenase activities were 0.574 and 1.28×10^{-3} for Exo^{Lead}, 9.26×10^{-3} and 2.05×10^{-3} for Exo^{Middle}, 0.488 and 1.07×10^{-5} for F90A, 0.408 and 9.34×10^{-3} for Y235A, 4.83×10^{-5} and 1.15×10^{-6} for W431A, and 1.09×10^{-4} and 1.19×10^{-3} for E416Q, respectively; *, $p < 0.01$; ns, non-significant ($p \geq 0.01$). Source data are provided.

Reviewer #3 (Remarks to the Author)

The quality and clarity of the revised manuscript have been significantly enhanced. The concerns raised in my previous comments have been thoroughly addressed, and I appreciate the considerate and detailed responses to my comments.

Upon review of the revised manuscript, four minor issues have arisen that need to be addressed prior to publication:

We are grateful for your thorough assessment of our revised manuscript and for the considerable time and effort you have devoted to evaluating our work. Your considerate and detailed comments have substantially contributed to enhancing the rigor and presentation of the manuscript. We have carefully considered the four remaining minor issues and provide our detailed responses below.

- Line 185: Please correct: "This distortion is critical for Zn2+-mediated polarization of the scissile carbonyl bond" instead of "chelation". The catalytic zinc ion functions as a Lewis acid in metallopeptidases.

Response: We appreciate the reviewer for giving us the opportunity to reconsider the wording 'chelation.' As its etymology suggests, 'chelation' (or 'chelate') refers to a structural arrangement in which a ligand binds a metal ion through multiple coordination points, resembling a crab grasping an object with its claws. Therefore, this term is not appropriate for describing the interaction between Zn^{2+} and a single scissile carbonyl oxygen.

Instead, we use the term 'coordination' to describe the structural arrangement in which the distorted scissile peptide bond is properly positioned relative to the Zn^{2+} ion. This coordination ensures precise alignment of the carbonyl oxygen with the catalytic metal centre.

Following correct coordination of the scissile carbonyl oxygen, the Zn^{2+} ion functions as a Lewis acid, accepting electron density from the oxygen atom and thereby enhancing polarisation of the carbonyl group. This polarisation increases the partial positive charge on the carbonyl carbon, which is essential for initiating nucleophilic attack by the activated water molecule (OH^-). We have added a brief description of this polarisation step in a separate paragraph within the catalytic centre section.

Revised (Lines 191-192):
"This distortion is critical for Zn^{2+} coordination of the scissile carbonyl oxygen."

Revised (Lines 230-232:
"At the catalytic centre, the scissile peptide carbonyl oxygen coordinated the Zn^{2+} ion, increasing polarisation of the carbonyl bond and priming it for nucleophilic attack. No ordered water molecules were observed near either the scissile bond or residue Gln416."

- The authors state in their response to the comment on former Lines 200–201 that ColH can translocate bidirectionally along the triple helix. To ensure consistency with this clarification, it would be appropriate to modify the wording in Lines 315–316 to "as it slides along the triple helix" instead of "from the N- to C-terminus."

Response: We thank the reviewer for this valuable suggestion. We have revised Lines 315–316 as recommended to ensure consistency in the description.

Revised (Lines 321-322):
"These residues may also help ColH recognise vulnerable sites in tropocollagen as it slides along the triple helix (Fig. 5c and d)."

- Supplementary Fig.16. Figure caption: Please provide the used PDB entry codes.

Response: We thank the reviewer for this valuable comment. The PDB entry codes have now been incorporated into the caption of Supplementary Fig. 16. The revised caption reads as follows:

Revised:

Supplementary Fig. 16 Ligand-induced contraction of the PD catalytic subdomain in M9B collagenases. Superposition of PD structures from $(POG)_{10}$-bound $ColH^{MT}$ (PDB ID: 9LQJ, this study) and substrate-free $ColH^{WT}$ (PDB ID: 9LRK, this study) (top); isoamylphosphonyl-Gly-Pro-Ala-bound (PDB ID: 2Y6I) and inhibitor-free (PDB ID: 2Y50) ColG (bottom left); and isoamylphosphonyl-Gly-Pro-Ala-bound (PDB ID: 4AR8) and inhibitor-free (PDB ID: 4AR9) ColT from *Clostridium tetani* (bottom right). In each comparison, superposition was performed using the N-terminal half-domain. Contraction of the C-terminal half-domain relative to the well-aligned N-terminal half-domain is indicated by arrows. The N-terminal and C-terminal half-domains are coloured grey and cyan, respectively, and the helper subdomain is coloured blue.

- Supplementary Fig.18: Please rephrase the figure caption to reflect that the presented model structure is the result of homology modelling using MOE with complex ColHMT-(POG)10 (PDB: 9LQJ) as initial template.

Response: We have revised the caption of Supplementary Fig. 18 to clearly state that the presented model structure was generated *in silico* based on PDB entry 9LQJ. We modified the figure caption as follows.

Revised:

Supplementary Fig. 18. Model structure of $ColH^{WT}$–Pz-peptide complex. The structural model of $ColH^{WT}$–Pz-peptide complex was generated *in silico* by reverting the single mutated residue to wild type and replacing the bound $(POG)_{10}$ peptide with the Pz-peptide in the $ColH^{MT}$–$(POG)_{10}$ structure (PDB ID: 9LQJ), followed by energy minimisation in Molecular Operating Environment. **a** Overall structure. **b** Enlarged view of active site in the complex. Pz-peptide bound to PD coordinates the zinc ion via the carbonyl oxygen of Leu2 (P1), and the adjacent Pro1 (P2) forms the CH–π and hydrophobic interactions with Trp431. The orange columns represent the carbon atoms of Pz-peptide.
